# Concordant Mode Approach (CMA): Vibrational Analysis of New and Upgraded Intermolecular Benchmarks for Noncovalent Bonding

**DOI:** 10.1021/acs.jpca.6c00689

**Published:** 2026-04-13

**Authors:** Laura N. Olive Dornshuld, Mitchell E. Lahm, Nathaniel L. Kitzmiller, Wesley D. Allen, Henry F. Schaefer

**Affiliations:** † Center for Computational Quantum Chemistry, 1355 University of Georgia, Athens, Georgia 30602, United States; ‡ Allen Heritage Foundation, Dickson, Tennessee 37055, United States; § Indiana Wesleyan University, Marion, Indiana 46953, United States

## Abstract

The Concordant Mode Approach (CMA) is a novel method that offers tremendous potential for increasing the system size and the level of theory attainable in quantum chemical computations of molecular vibrational frequencies. To investigate the extension of CMA to intermolecular vibrations, computations with coupled cluster singles and doubles with perturbative triples theory [CCSD­(T)] using two augmented correlation-consistent polarized-valence triple-ζ basis sets (aug-cc-pVTZ or h-aug-cc-pVTZ) were performed on 17 prototypical loosely bound complexes of hydrogen-bonded, dispersion, and mixed character. These Level A results provide new and upgraded benchmarks for noncovalent bonding and a severe test for CMA vibrational analyses. The Level A target frequencies were recovered remarkably well using second-order Møller–Plesset perturbation theory (MP2) with h-aug-cc-pVTZ for generating the underlying (Level B) normal modes of the CMA scheme. Employing this Level B within the lowest-rung CMA-0A method reproduces the 435 benchmark frequencies with a mean absolute error (MAE) of 0.23 cm^–1^ and a corresponding standard deviation (σ) of 0.84 cm^–1^; strikingly, the corresponding subset of 106 interfragment frequencies exhibits MAE = 0.34 cm^–1^ and σ = 0.90 cm^–1^. Subsequent application of the higher-rung CMA-2A scheme eliminates all outliers and reduces the overall MAE to a minuscule 0.08 cm^–1^ with the inclusion of only 3.0% of the off-diagonal couplings not accounted for by CMA-0A. Accordingly, the highly efficient CMA methodology proves to be robust even for vibrations on flat potential energy surfaces.

## Graphical abstract

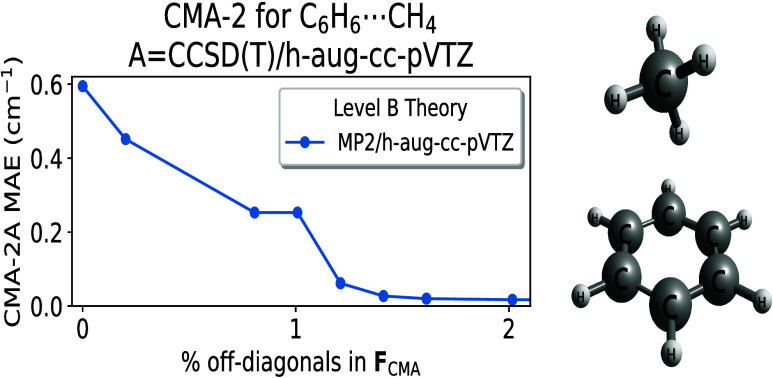

## Introduction

The precise physical description of noncovalent interactions in chemical systems using robust electronic structure methods has constituted a significant challenge to theory. This challenge permeates out to properties such as vibrational frequencies, which are fundamentally important for spectroscopic assignment and thermodynamic analysis. While quantitative accuracy can be obtained by methods with a high-level treatment of electron correlation such as coupled-cluster theory with singles, doubles, and perturbative triples, CCSD­(T),^1,2^ the steep *O*(*N* ^7^) computational demands of CCSD­(T) significantly limit the possible number of atoms and basis set size. Considerable progress has been made in the literature of noncovalent systems,^3^ and the interaction energies of systems with up to 24 atoms have been estimated at the CCSD­(T) level using complete basis set (CBS) extrapolation.^4–7^ Rigorous electronic structure computations on small model systems provide the first step toward definitively describing noncovalent complexes.

Numerous benchmark interaction energy databases for noncovalent dimer complexes (hydrogen-bonded, dispersion bound, and mixed complexes) have been compiled utilizing high-quality quantum mechanical computations.^8–14^ These databases are useful for determining appropriate methods for larger systems, because they document the performance of lower scaling *ab initio* methods and density functional theory approximations. Despite the plethora of data for the benchmark energetics of dimer systems, vibrational frequencies computed with highly accurate *ab initio* methods are far less common. Therefore, it is possible that the global minimum-energy conformations of these systems have not been determined due to the prohibitive cost of calculating the vibrational frequencies at a high level of theory. Obtaining highly accurate interfragment vibrational frequency benchmarks is a challenging task that nevertheless holds the promise of elucidating the noncovalent effects in larger complexes.

A recent, novel method coined the Concordant Mode Approach (CMA)^15,16^ holds great potential for the accurate computation of vibrational frequencies, because it reduces the scaling with respect to system size of necessary single-point energy computations at a target level of theory from quadratic to linear, speeding up computations by about an order of magnitude on the systems studied thus far. The CMA-0A protocol, which centers on computing only diagonal force constants at the higher level of theory A in a normal mode basis generated by a lower level of theory B, has recently been applied to over 120 molecules (1581 targeted CCSD­(T)/cc-pVTZ benchmark vibrational frequencies) from the G2 test set.^17^ Remarkably, CMA-0A reduces all except three and seven frequency residuals to less than 2.5 cm^–1^ when Level B is chosen as B3LYP/6-31G­(2*df*,*p*) and CCSD­(T)/cc-pVDZ, respectively. These remaining outliers were reduced below about 1 cm^–1^ by the inclusion of just a single off-diagonal force constant per outlier pair. This method of manually selecting off-diagonal force constant elements to compute was labeled CMA-1A.

Further research has created a new method (CMA-2A) that rapidly and systematically eliminates all outliers and converges to the exact Level A frequencies.^16^ The CMA-2A protocol automatically selects which off-diagonal force constants to explicitly evaluate at Level A based on dimensionless ξ parameters that can be evaluated at an auxiliary Level C requiring little to no further computational cost. When Level B = MP2/cc-pVTZ and Level C = HF/cc-pVTZ, a cutoff of ξ = 0.02 reduced the MAE and maximum residual of the entire database to less than 0.05 and 1.8 cm^–1^, respectively, while only incurring an additional 33% increase in computational cost from CMA-0A. Nevertheless, hand-selection of couplings in the CMA-1A scheme reveals that gains in the efficiency of automated procedures beyond CMA-2A are still possible, as all residuals can be reduced below 1.1 cm^–1^ by including only a single off-diagonal force constant per outlier pair. The present research applies the CMA-0A, CMA-1A, and CMA-2A protocols to nonbonded chemical interactions for the first time by studying the 17 complexes shown in Figures 1, 2, 3. Most of these systems are taken from the S22 data set,^8^ and they cover all three subgroups therein: hydrogen-bonded complexes [(H_2_O)_2_, (NH_3_)_2_, H_2_O···NH_3_, (HCOOH)_2_, CH_3_NO···NH_3_, (HCONH_2_)_2_, C_2_H_2_···H_2_O, CH_3_COOH···H_2_O, (H_2_O)_3_, (HF)_2_], complexes with predominant dispersion stabilization [(C_2_H_4_)_2_, (CH_4_)_2_, C_6_H_6_···CH_4_], and mixed complexes in which electrostatic and dispersion contributions are similar in magnitude [C_6_H_6_···HCN, C_6_H_6_···H_2_O, C_6_H_6_···NH_3_, C_2_H_4_···C_2_H_2_]. Because intermolecular vibrations occur on flat potential energy surfaces, these species provide a severe test for the CMA-*N* hierarchy. Nevertheless, the work presented here shows that this test is passed with flying colors.

**Figure 1:**
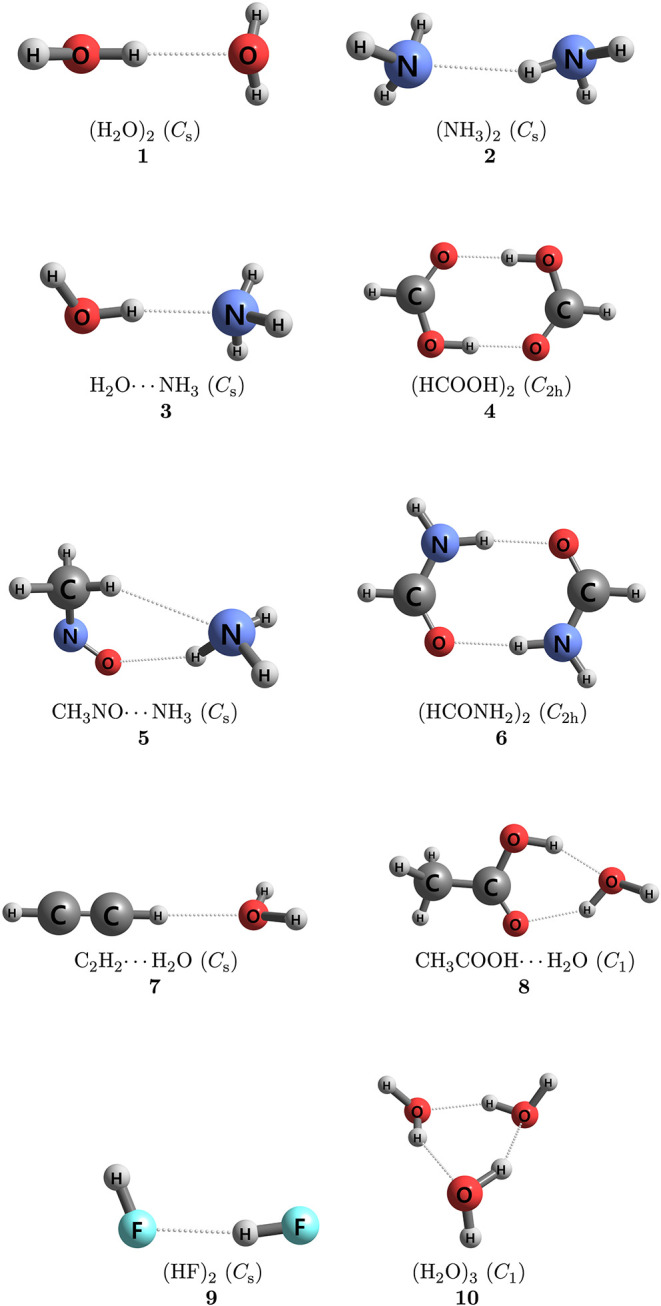
Hydrogen-bonded complexes.

**Figure 2:**
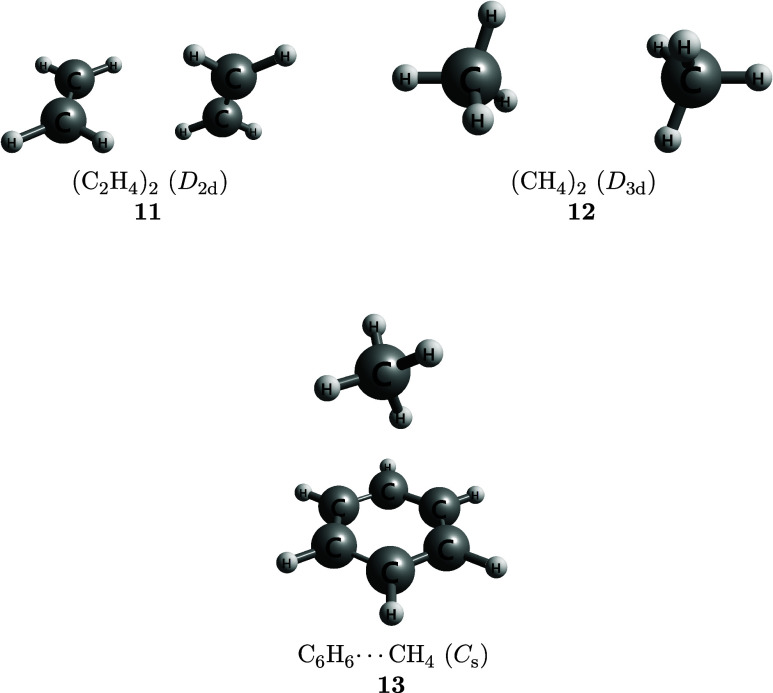
Dispersion-bound complexes.

**Figure 3:**
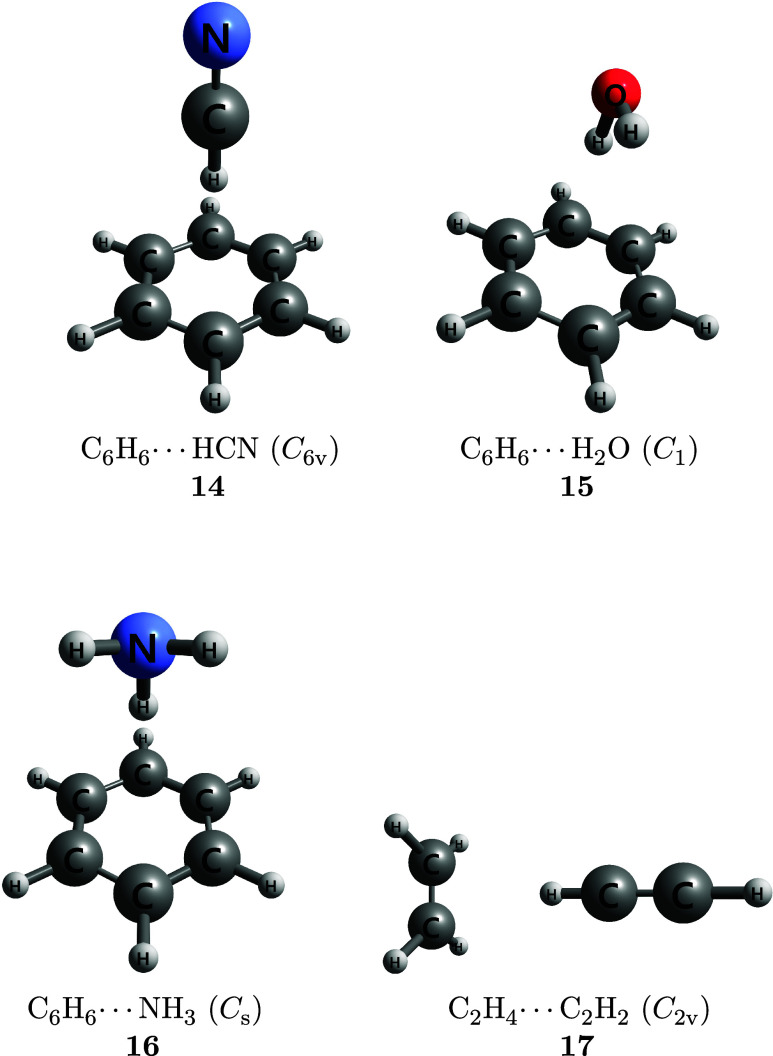
Mixed complexes in which dispersion and electrostatic contributions to the binding energy are similar in magnitude.

## Computational Methods

Full geometry optimizations were performed for Level A using the rigorous CCSD­(T)^18^ method and in most cases Dunning’s correlation consistent triple-ζ basis set augmented with diffuse functions, aug-cc-pVTZ.^19^ However, as discussed below, for the benzene-containing complexes, the CCSD­(T) method with the h-aug-cc-pVTZ basis set^19,20^ was used for geometry optimizations. Quadratic force fields and harmonic vibrational frequencies for Level A and Level B were computed with the Hartree–Fock (HF), second-order Møller–Plesset perturbation (MP2),^21^ and CCSD­(T) methods in conjunction with the following correlation-consistent basis sets of double-, and triple-ζ quality: cc-pV*X*Z,^20^ h-aug-cc-pV*X*Z, and aug-cc-pV*X*Z,^19^ where *X* = {D, T} is the cardinality. These basis sets are denoted here as *X*Z, ha*X*Z, and a*X*Z, respectively.

The detailed protocols outlined previously for CMA-0A,^15^ CMA-1A,^15^ and CMA-2A^16^ were followed for this study. The Level B quadratic force constants and gradients were computed in Cartesian coordinates at the Level A reference geometries via finite-difference formulas with fourth-order accuracy and a displacement size of 0.01 bohr. An exact, second-order scheme^22^ was utilized to transform the Cartesian force constants and gradient into a proper natural internal coordinate (NIC) basis.^23,24^ The corresponding vibrational normal modes were computed with the GF-matrix method.^25,26^ The reference force constants of Level A were transformed from the Cartesian basis to the calculated normal mode basis of Level B to obtain the matrix **F**_CMA_.^15,16^

In order to completely eliminate numerical errors in the current benchmark research, the CMA frequencies were obtained by zeroing out off-diagonal elements of the reference **F**_CMA_ according to the employed CMA method. Furthermore, symmetry was rigorously applied to relevant systems by zeroing out numerical residuals of **F**_CMA_ force constants between coordinates belonging to differing irreducible representations. Non-Abelian symmetry was further imposed by zeroing out **F**_CMA_ interactions between degenerate blocks and then averaging to eliminate any small numerical errors. The HF, MP2, and CCSD­(T) energies were computed with the Molpro program,^27^ and the SCF and CC energy residuals were reduced to 10^–12^ Hartrees upon wave function convergence. Core electrons were frozen in all coupled cluster and MP2 correlation treatments. The optimized geometries and vibrational frequencies at the target level of theory, as well as the NICs employed, can be found for all species in the voluminous Supporting Information (SI).

### Benzene Conundrum

The benzene molecule is well-known for spectacular problems in the application of atomic orbital basis sets.^28^ A new predicament within this legacy surfaced in our CMA research, requiring computations with the partially augmented, h-aug-cc-pVTZ basis set. The h-aug-cc-pVTZ basis set incorporates diffuse functions only on the non-hydrogen (“heavy”) atoms, while adopting cc-pVTZ for H. This choice is necessitated by insidious near linear dependencies in the aug-cc-pVTZ basis set for benzene. When harmonic frequencies are computed for benzene at the CCSD­(T)/aug-cc-pVTZ level of theory atop a *D*_6h_ structure optimized at the same level, the ω_8_(*b*_2g_) ring puckering (chair) mode exhibits a spurious frequency of 495*i* cm^–1^ and thus gives rise to a nonplanar minimum! Samala and Jordan^29^ found similar anomalies at the MP2/aug-cc-pVTZ level and performed a preliminary analysis of the problem. Table S1 of the SI demonstrates that our CCSD­(T)/h-aug-cc-pVTZ benzene frequencies show fine agreement with the empirically derived harmonic frequencies of Handy and co-workers,^30^ except for a remaining 72 cm^–1^ discrepancy for ω_8_. However, it is quite possible that the empirical ω_8_ is substantially in error, because it was derived using a suspect anharmonicity computed at the RHF/DZP level of theory.

Recent developments germane to this benzene conundrum are reported in a 2023 paper^31^ that includes harmonic and anharmonic vibrational frequencies computed from an all-electron CCSD­(T)/cc-pCVTZ quartic force field. According to the reported computations, ω_8_ = 667 cm^–1^, which is raised by a very peculiar anharmonicity to ν_8_ = 781 cm^–1^. In stark contrast, the empirical frequencies^30^ are (ω_8_, ν_8_) = (718, 707) cm^–1^. This quandary for a paradigmatic small molecule is surprising, given the advanced state of both computational quantum chemistry and vibrational spectroscopy. While meticulous future research is clearly needed, the (ω_8_, ν_8_) problem has little bearing on the goal of the current paper to investigate the ability of CMA methods to reproduce full vibrational frequency computations at a given level of theory.

## Interfragment Internal Coordinates

For the application of CMA to intermolecular motions of dimer systems, some special internal coordinates were utilized, defined as follows and fully elaborated upon in the SI. For a dimer containing *N* total atoms, where the monomers are denoted by A and B, select three noncollinear points within each monomer as functions of the nuclear positions only within A or B: **d**_*K*_^A^(**r**_1_^A^, **r**_2_^A^, ..., **r**_*m*_^A^) and **d**_*K*_^B^(**r**_1_^B^, **r**_2_^B^, ..., **r**_*n*_^B^) (*K* = 1, 2, 3), where the Cartesian coordinates are denoted as **r**_*i*_^A^ (*i* = 1, 2, ..., *m*) and **r**_*j*_^B^ (*j* = 1, 2, ..., *n*), and *m* + *n* = *N*. These monomer points are used to define a set of chemically motivated internal coordinates involving intermolecular distances and Euler angles. If one monomer is linear, an interfragment rotational degree of freedom is absent; only two **b**_*K*_ points are needed, but dummy atoms may be employed to specify intrafragment linear bending coordinates. Atomic centroids may be utilized for any or all of the three monomer points. Unit vectors connecting two points, **d**_*K*_ and **d**_*L*_, are defined as

1 $$e_{\mathrm{KL}}^{P}=\frac{\left(d_{L}^{P}-d_{K}^{P}\right)}{\left|d_{L}^{P}-d_{K}^{P}\right|}$$

2 $$e_{R}^{P}=\frac{\left(d_{1}^{Q}-d_{1}^{P}\right)}{\left|d_{1}^{Q}-d_{1}^{P}\right|}$$

The first interfragment coordinate (*R*), which appears as a label rather than an index in eq 2, is the monomer-monomer distance

3 $$R=\left|d_{1}^{A}-d_{1}^{B}\right|$$

The polar angles (θ_*P*_) of the monomers with respect to the axis connecting the **d**_1_ centers are the next two interfragment coordinates, given by

4 $$\cos\theta_{P}=e_{12}^{P}\cdot e_{R}^{P}$$

where (*P, Q*) = (*A, B*) or (*B, A*) and **e**_12_^*P*^ is an intrafragment vector. The monomer-monomer torsion angle, which corresponds to the difference between the azimuthal Euler angles ϕ_A_ and ϕ_B_ of the two monomers, may be defined as

5 $$\cos\tau =\frac{\left(e_{12}^{A}\times e_{R}^{A}\right)\cdot \left(e_{R}^{B}\times e_{12}^{B}\right)}{\sin\theta_{A}\sin\theta_{B}}=\frac{\cos\theta_{A}\cos\theta_{B}+e_{12}^{A}\cdot e_{12}^{B}}{\sin\theta_{A}\sin\theta_{B}}$$

Finally, the two monomer internal rotation angles are defined as

6 $$\sin\chi_{P}=\frac{e_{32}^{P}\cdot \left(e_{R}^{P}\times e_{12}^{P}\right)}{\sin\theta_{P}\sin\alpha_{P}}$$

where

7 $$\cos\alpha_{P}=e_{32}^{P}\cdot e_{12}^{P}$$

and *P* = A or B. In defining the χ_*p*_ angles, **d**_3_^*P*^ is assumed to lie in the monomer body-fixed *yz* plane. The B-tensor elements for our interfragment Euler-angle coordinates can be attained merely by modifying standard formulas to use the generalized points **d**_*K*_ rather than nuclear positions, followed by differentiation of the **d**_*K*_ points with respect to the nuclear positions. Use of interfragment Euler-angle coordinates here is an important advancement in the vibrational analysis of dimer systems. Pending a more general formalism for trimer systems, the coordinates for (H_2_O)_3_ were chosen by considering the hydrogen-bonded framework as a six-membered ring possessing customary natural internal coordinates. Each of the remaining three O–H bonds external to the ring were assigned a stretch, wag, and rock coordinate.

## Geometries and Binding Energies

Our CCSD­(T)/aTZ optimized structures for complexes **1**–**4**, **6**, **7**, **9**, **10** and **12** are compared to the best previously reported structures of the same symmetry in Table 1, which presents chemically relevant interfragment coordinates and maximum absolute differences in intrafragment coordinates, as well as binding energies and differences in them. A careful examination of the data was undertaken to ensure the chemical validity of our CMA benchmarks. In Table 1, the mean absolute error (MAE) of the critical interfragment distances is 0.0053 Å with a standard deviation (σ) of 0.0077 Å. Among the interfragment angles, intrafragment coordinates, and binding energies (BE), four outliers warrant removal from statistical consideration. As discussed below, these outliers involve *r*_CO_, θ_HCN_, and BE of complex **6** as well as the out-of-plane angle γ_H···OHH_ of complex **7**. For the remaining data set, the MAE of the critical interfragment angles is 0.9° with σ = 0.7°. The intrafragment coordinate (MAE, σ) values are (0.0030 Å, 0.0015 Å) for distances and (0.2°, 0.2°) for angles, respectively, whereas (MAE, σ) = (0.21, 0.15) kcal mol^–1^ for binding energies. Overall, the structures and binding energies computed here show very good agreement with the best prior literature, and the corresponding complexes provide dependable CMA benchmarks.

**Table 1:** Comparisons of CCSD­(T)/aTZ Optimized Results of This Study With Best Previous Literature Values^a^

complex	critical distance	critical angle	max |ΔGeom|^b^	ΔBE (BE)^c^ (kcal mol^–1^)
(H_2_O)_2_ ^d^ (**1**)	Δ*r*_O···H_ = –0.0005 Å	Δ*γ*_H···OHH_ = −1.6°	0.0040 Å, 0.4°	0.20 (5.02)
	AE-CCSDTQ/CBS^32^			AE-CCSDTQ/CBS^32^
(NH_3_)_2_ (**2**)	Δ*r*_N···H_ = −0.0023 Å	Δ*θ*_NH···N_ = −1.2°	0.0048 Å, 0.5°	0.13 (3.14)
	MP2/aTZ^33^			CCSD(T)/a5Z^34^
H_2_O···NH_3_ ^e^ (**3**)	Δ*r*_N···H_ = 0.0109 Å	Δ*θ*_OH···N_ = −0.7°	0.0031 Å, 0.2°	0.12 (6.47)
	CCSD(T)/CBS^35^			CCSD(T)/CBS^35^
(HCOOH)_2_ (**4**)	Δ*r*_O···H_ = 0.0038 Å	Δ*θ*_OH···O_ = −0.7°	0.0034 Å, 0.2°	0.30 (16.49)
	CCSD(T)/aQZ^36^			CCSD(T)/aQZ^36^
(HCONH_2_)_2_ (**6**)	Δ*r*_O···H_ = 0.0066 Å	Δ*θ*_NH···O_ = 0.6°	0.0056 Å, 7.7°	0.73 (14.73)
	CCSD(T)/CBS^37^			CCSD(T)/CBS^37^
C_2_H_2_···H_2_O (**7**)	Δ*r*_O···H_ = 0.0030 Å	Δ*γ*_H···OHH_ = 6.1°	0.0001 Å, 0.1°	0.00 (3.20)
	CCSD(T)/aTZ^38^			CCSD(T)/aTZ^38^
(HF)_2_ (**9**)	Δ*r*_F···H_ = −0.0005 Å	Δ*θ*_HF···H_ = 0.8°	0.0037 Å	0.32 (4.51)
	CCSD(T)/ha5Z^39^			CCSD(T)/CBS^35^
(H_2_O)_3_ (**10**)	Δ*r*_O···H_ = 0.0004 Å	Δ*θ*_OH···O_ = −0.5°	0.0028 Å, 0.2°	0.49 (15.79)
	(T):MP2/haQZ^40^			(T):MP2/haQZ^40^
(CH_4_)_2_ (**12**)	Δ*r*_C···C_ = −0.0166 Å		0.0018 Å, 0.0°	0.12 (0.52)
	CCSD(T)/CBS^35^			CCSD(T)/aQZ^41^

a The criterion of fully optimized structures is adopted here in selecting best previous literature values. The corresponding level of theory is given beneath the numerical differences for each complex.

b The largest absolute differences in distance and angle (where applicable) within the set of intrafragment coordinates.

c Reported vibrationless binding energy (BE) at referenced level of theory.

d A composite method including core correlation, relativistic, and DBOC corrections is utilized in ref 32 to get the AE-CCSDTQ/CBS geometries and energies.

e The *E* ^CCSD(T)^ – *E* ^MP2^ correlation increment is calculated with the small aDZ basis set in ref 35.

The largest deviation in critical bond distances in Table 1 occurs for *r*_C···C_ of the methane dimer (**12**), for which CCSD­(T)/CBS and CCSD­(T)/aTZ yield 3.6380 and 3.6214 Å, respectively. This difference is nonetheless satisfactory given the extremely flat PES with a binding energy of only about 0.5 kcal mol^–1^. For critical angles involving monomer orientation, the only absolute deviation larger than 1.6° is found for the C_2_H_2_···H_2_O complex (**7**). Reference 38 reports an out-of-plane wagging angle of γ_H···OHH_ = 24.0° at CCSD­(T)/aTZ; however, at the same level of theory we find a substantially different value of γ_H···OHH_ = 30.0°. Because these results should be identical, we redoubled our efforts and confirmed our CCSD­(T)/aTZ structure by finding that it is obtained by multiple programs, viz., CFOUR, PSI4, and Molpro. Tight convergence in the geometry optimizations is essential because the surface is so flat, and the optimum geometry is extremely sensitive to basis set. Indeed, in going from aTZ to aQZ, the optimized CCSD­(T) wagging angle is reduced from 30° to 16°; however, the change in the CCSD­(T)/aTZ energy between the two structures is a mere 10 cm^–1^.

The largest differences in intrafragment coordinates and binding energy in Table 1 all arise from the hydrogen-bonded (HCONH_2_)_2_ complex (**6**), for which an optimized CCSD­(T)/CBS structure has been reported by Alessandrini and Puzzarini.^37^ With respect to their benchmark, our CCSD(T)/aTZ results for (*r*_CO_, BE) differ by (0.0056 Å, 0.73 kcal mol^–1^), while an unreasonably large disparity of 7.7° occurs for θ_HCN_. Our *r*_CO_ and θ_HCN_ values are in good agreement with those of the earlier RI-MP2/aQZ structure of Frey and Leutwyler,^42^ as well as additional CCSD­(T)/aQZ, CCSD­(T)/haTZ, and CCSD­(T)/TZ results we computed for comparison (SI, Tables S21 and S22). In ref 37 it was speculated that a typographical error for θ_HCN_ was made in ref 42; however, it is now apparent that the fault actually lies with the CCSD­(T)/CBS structure. One feature that vitiates the reported CCSD­(T)/CBS results is that the stiff valence bond angle θ_HCN_ within each monomer is predicted to change upon dimerization by an excessive 8.8°.

To our knowledge, the best structures and energetics computed here for complexes **5**–**8**, **11**, and **13**–**17** are at the highest level of theory available in the current literature. Critical distances, angles, and the binding energy of each complex as well as the level of theory are summarized in Table 2. Full information for these complexes is given in the SI. Complexes **6**, **7**, **11**, and **13**–**17** come from the S22 test set, and the results presented here are substantial improvements to the benchmarks therein. While not a part of the S22 set, our findings for complexes **5** and **8** are an important advancement to the literature for hydrogen-bonded systems. In order to resolve the disagreements between our CCSD­(T)/aTZ computations and earlier literature, we also provide new results from higher levels of theory for complexes **6** and **7** in Table 2.

**Table 2:** New Benchmark Optimized Structures and Binding Energies^a^

complex	critical interfragment coordinates			BE (kcal mol^–1^)
CCSD(T)/h-aug-cc-pVTZ				
C_6_H_6_···CH_4_ (**13**)	*r*(*X*_c_|H_X_) = 3.700 Å	θ(C_σ_|*X*_c_|H_X_) = 93.6°	θ(Y_C_|*X*_c_|H_X_) = 90.0°	1.67
C_6_H_6_···HCN (**14**)	*r*(*X*_c_|H_X_) = 2.309 Å	θ(C_σ_|*X*_c_|H_X_) = 90.0°	θ(Y_C_|*X*_c_|H_X_) = 90.0°	4.92
C_6_H_6_···H_2_O (**15**)	*r*(*X*_c_|H_X_) = 2.484 Å	θ(C_σ_|*X*_c_|H_X_) = 76.8°	θ(Y_C_|*X*_c_|H_X_) = 90.2°	3.54
C_6_H_6_···NH_3_ (**16**)	*r*(*X*_c_|H_X_) = 2.549 Å	θ(C_σ_|*X*_c_|H_X_) = 82.3°	θ(Y_C_|*X*_c_|H_X_) = 90.0°	2.51
CCSD(T)/aug-cc-pVTZ				
CH_3_NO···NH_3_ (**5**)	*r*_O···H_ = 2.392 Å	*r*_N···H_ = 2.559 Å	θ_O··· HN_ = 148.9°	3.24
CH_3_COOH···H_2_O (**8**)	*r*_H_2_O···H_a__ = 1.799 Å	*r*_O···HOH_ = 1.948 Å	γ_H_a_···OH_2__ = 55.0°	10.66
(C_2_H_4_)_2_ (**11**)	*r*(X_C_|X_C_^′^) = 3.695 Å	τ_CX_C_···X_C_^′^C′_ = 90.0°		1.76
C_2_H_4_···C_2_H_2_ (**17**)	*r*(*X*_c_|H_X_) = 2.682 Å	θ(C|*X*_c_|H_X_) = 90.0°		1.84
CCSD(T)/aug-cc-pVQZ				
(HCONH_2_)_2_ (**6**)	*r*_O···H_ = 1.846 Å	θ_NH···O_ = 173.2°		15.13
CCSD(T)/aug-cc-pV5Z				
C_2_H_2_···H_2_O (**7**)	*r*_O···H_ = 2.197 Å	γ_H···OHH_ = 15.9°		2.93

a BE is the vibrationless binding energy. *X*_c_ denotes the centroid of the carbon atoms of benzene or ethylene. H_X_ refers to the hydrogen atom on the complementary dimer unit closest to the centroid, *X*_c_. C_σ_ is the benzene carbon atom defining the (pseudo) plane of symmetry. Y_C_ is a bond midpoint within the benzene ring selected so that the *X*_c_ → Y_C_ and *X*_c_ →C_σ_ vectors are perpendicular. H_a_ denotes an acidic proton. Notation, atomic indices, and full geometric information are given in the SI.

## Benchmarking Level B for CMA-0A

The CMA-0A procedure targeting our aTZ or haTZ CCSD­(T) reference data was applied to complexes **1**–**17** for 11 Level B methods, as summarized in Table 3 and documented fully in the SI. As shown by the size (*N*) of each data set, computations for each Level B were not exhaustive but sufficient to identify the best performers, such as MP2/haTZ with *N* = 435 results. Moreover, the CCSD­(T)/haTZ data (*N* = 219) only include the cases for which Level A = CCSD­(T)/aTZ. The mean absolute error (MAE ϵ) of the CMA-0A residuals goes from 1.40 cm^–1^ at B = MP2/DZ down to a mere 0.04 cm^–1^ for B = CCSD­(T)/haTZ. Similarly, mean ϵ = (0.45, 0.01) and σ_ϵ_ = (3.79, 0.12) cm^–1^ characterize the ranges with the endpoints B = (CCSD­(T)/DZ, CCSD­(T)/haTZ) for ϵ and B = (MP2/DZ, CCSD­(T)/haTZ) for σ_ϵ_. The minuscule values for mean ϵ highlight the substantial degree of error cancellation present in CMA-0A, because the frequencies change in nearly equal and opposite magnitudes as the normal modes mix beyond Level B. The range of the % outliers is (11.2, 0.0)% for the Level B choices, with (MP2/DZ, CCSD­(T)/haTZ) once again forming the end points. In summary, CMA-0A performs very well for most levels of theory, but sparse outliers still remain that justify higher rungs of the CMA hierarchy.

**Table 3:** Summary Statistics of Residuals in Harmonic Frequencies (*ϵ*, cm^–1^) and ZPVEs (Δ, cm^–1^) given by CMA-0A targeting Level A = CCSD­(T)/aTZ or CCSD­(T)/haTZ

Level B	*N* ^a^	MAE ϵ	mean ϵ	ϵ_max_ ^b^	σ_ϵ_	% outliers^c^	MAE Δ	mean Δ	σ_Δ_
MP2/DZ	243	1.40	0.41	10.37	3.79	11.9	4.20	4.20	4.27
MP2/haDZ	243	0.44	0.07	2.90	1.95	1.7	0.70	0.70	0.36
MP2/aDZ	243	0.75	0.14	6.17	3.81	3.3	1.41	1.41	1.34
MP2/TZ	408	0.52	0.19	4.24	1.84	6.1	1.88	1.88	3.26
MP2/haTZ	435	0.23	0.06	1.79	0.84	3.0	0.81	0.81	1.34
MP2/aTZ	243	0.22	0.03	1.49	1.17	1.2	0.23	0.23	0.32
CCSD(T)/DZ	243	1.29	0.45	9.08	3.59	10.7	3.67	3.67	4.64
CCSD(T)/haDZ	243	0.38	0.08	2.52	1.57	1.2	0.76	0.76	0.50
CCSD(T)/aDZ	219	0.48	0.12	3.78	1.70	3.6	1.24	1.24	1.55
CCSD(T)/TZ	219	0.32	0.12	2.29	0.94	3.7	1.16	1.16	1.27
CCSD(T)/haTZ	219	0.04	0.01	0.27	0.12	0.0	0.10	0.10	0.10

a Number of benchmark vibrational frequencies included.

b Average maximum absolute ϵ per molecule.

c Percentage of the *N* residuals with magnitude greater than 2.5 cm^–1^.

The zero-point vibrational energy (ZPVE) MAE residuals (MAE Δ) and ZPVE residual standard deviations (σ_Δ_) are no larger than 4.20 cm^–1^ (MP2/DZ) and 4.64 cm^–1^ (CCSD­(T)/haTZ), respectively, for the 11 Level B choices. These statistical signatures for the ZPVEs are somewhat larger than those observed previously,^15,16^ but they are still negligible in thermochemical applications. A curious mathematical fact is that the MAE and mean Δ values are exactly the same, indicating a systematic bias in which all ZPVE residuals are positive. This phenomenon is inherent to the CMA-0A method, arising because for any symmetric, real-valued matrix with all positive diagonal elements and eigenvalues, the sum of the square roots of the diagonal values will always be greater than or equal to the sum of the square roots of the eigenvalues. This theorem is proved rigorously in Section S1.2 of the SI, and its manifestations are present in all of our prior work,^15,16^ although unnoticed heretofore.

Within the G2 test set, Kitzmiller and co-workers^16^ showed that basis set quality is more important than higher-order treatment of electron correlation in the selection of Level B. Quite remarkably for our 17 complexes, the MAE ϵ values for MP2/haTZ and MP2/aTZ are 0.23 and 0.22 cm^–1^, respectively, only marginally larger than the 0.04 cm^–1^ for CCSD­(T)/haTZ. Furthermore, MP2/haTZ and MP2/aTZ face the most demanding test of all Level B choices here, as they were benchmarked on the largest sample and were the only methods utilized for the benzene-containing heterodimers. Once again, the best choice for Level B is MP2 with a large basis set (aTZ or haTZ) rather than CCSD­(T) with a smaller basis set (DZ, haDZ, aDZ), as demonstrated by the superior MP2 residual statistics in Table 3 as well as the dramatically better MP2 scaling with respect to system size (conventional *N* ^5^ vs *N* ^7^). Finally, we recommend MP2/haTZ over MP2/aTZ for Level B when A = CCSD­(T)/aTZ because it still has superb accuracy, is lower in cost, and avoids issues with linear dependencies in the basis set.

The importance of the basis set quality in the accurate description of normal modes is further shown by the water dimer residuals in Table 4. Perhaps unsurprisingly, MP2 and CCSD­(T) with the unaugmented DZ basis set yield the largest residuals, most notably (24.3, 17.0) cm^–1^ and (25.7, 14.0) cm^–1^, respectively, for (ω_8_, ω_11_). Both ω_8_(*a*′) and ω_11_(*a*″) correspond to interfragment rotations, however they differ in symmetry. Moving to the haDZ basis set brings the residuals of these modes down to (2.0, 1.9) cm^–1^ and (1.3, 2.8) cm^–1^, respectively. Further improvement can be seen moving to the aDZ basis set, where the residuals decrease to (0.4, 1.0) cm^–1^ and (−1.2, 3.2) cm^–1^, respectively. Results with the unaugmented TZ basis set are significantly better than those of the unaugmented DZ basis set; nevertheless, three residuals above the 2.5 cm^–1^ threshold occur for both MP2 and CCSD­(T). Moving to the haTZ basis set with MP2 reduces all residuals below 0.5 cm^–1^, rivaling MP2/aTZ in accuracy while using a smaller set of basis functions. In fact, B = MP2/haTZ produces a MAE of 0.08 cm^–1^, the lowest among all tested Level B choices.

**Table 4:** CMA-0A Residuals with Respect to the Reference Harmonic Frequencies (in cm^–1^) for (H_2_O)_2_

	Reference	Level B						Level B				
	CCSD(T)	MP2						CCSD(T)				
	aTZ	DZ	haDZ	aDZ	TZ	haTZ	aTZ	DZ	haDZ	aDZ	TZ	haTZ
ω_1_(*a*′)	3891.4	–1.1	–0.1	–0.1	–0.1	0.0	0.0	–1.7	–0.1	–0.1	–0.1	0.0
ω_2_(*a*′)	3805.5	–0.1	0.0	0.0	0.0	0.0	0.0	0.0	0.0	0.0	0.0	0.0
ω_3_(*a*′)	3731.1	1.2	0.0	0.1	0.1	0.0	0.0	1.8	0.0	0.1	0.1	0.0
ω_4_(*a*′)	1667.5	–0.1	0.0	0.0	0.0	0.0	0.0	–0.1	0.0	0.1	0.0	0.0
ω_5_(*a*′)	1646.3	0.1	0.0	0.0	0.1	0.0	0.0	0.1	0.0	0.0	0.1	0.1
ω_6_(*a*′)	359.8	–0.2	–0.7	–0.3	0.0	0.0	0.0	–0.3	–0.8	–0.3	0.0	0.0
ω_7_(*a*′)	185.1	–5.8	–0.1	0.0	–2.6	0.0	–0.2	–5.3	0.5	–0.4	–2.5	–0.1
ω_8_(*a*′)	153.5	24.3	2.0	0.4	–0.4	0.0	0.3	25.7	1.3	–1.2	2.1	0.1
ω_9_(*a*″)	3911.1	0.0	0.0	0.0	0.0	0.0	0.0	0.1	0.1	0.0	0.0	0.0
ω_10_(*a*″)	621.8	–9.4	–0.4	0.2	–2.0	0.3	0.3	–9.0	–0.2	0.2	–2.0	0.2
ω_11_(*a*″)	143.4	17.0	1.9	1.0	7.1	0.1	0.5	14.0	2.8	3.2	7.1	0.0
ω_12_(*a*″)	130.0	2.0	1.0	0.1	6.8	0.5	0.1	5.0	1.3	0.1	3.9	0.7

An abnormal case for CMA-0A performance can be seen in the residuals collected in Table 5 for the formic acid dimer (**4**). All 11 Level B choices describe the interfragment modes of (HCOOH)_2_ very well, but the intrafragment in-phase O–H (ω_1_) and C–H (ω_2_) stretching modes are poorly captured by DZ basis sets. For the formic acid monomer at CCSD­(T)/aTZ, the (C–H, O–H) stretching frequencies are (3088.0, 3742.2) cm^–1^ with total energy distributions (TEDs)^43–45^ of (99.5, 100.0)% for the corresponding simple internal coordinates. The 654 cm^–1^ gap between these vibrational modes quenches any mixing in the monomer, but in the formic acid dimer, strong O–H···O hydrogen bonds are formed (BE = 16.5 kcal mol^–1^) and the O–H stretching frequencies are greatly reduced. Consequently, both the symmetric [ω_1_(*a*_g_)] and antisymmetric [ω_17_(*b*_u_)] O–H stretches move into the same energetic region as the C–H stretches (ω_2_, ω_18_). Nevertheless, in the CCSD­(T)/aTZ reference structure, the differences δ_1,2_ = ω_1_ – ω_2_ = 92.1 cm^–1^ and δ_17,18_ = ω_17_ – ω_18_ = 199.7 cm^–1^ are still too large to facilitate mixing, and the corresponding modes of vibration remain pure (leading TED values ≥ 95%). In contrast, five Level B choices with a basis set of DZ cardinality [MP2/DZ, MP2/haDZ, MP2/aDZ, CCSD­(T)/haDZ, CCSD­(T)/aDZ] yield Level B (ω_1_, ω_2_) frequencies with a mean δ_1,2_ = 16.0 cm^–1^ and corresponding normal modes with significant mixing (mean leading TED element = 81%). For this set of Level B choices, large CMA-0A residuals between 5.9 and 39.2 cm^–1^ in magnitude are observed for ω_1_ and ω_2_, because the corresponding normal modes incorrectly mix the C–H and O–H stretches. Meanwhile, the mean δ_17,18_ = 119.1 cm^–1^ in this set remains sufficient to avoid spurious mixing and limit the ω_17_ and ω_18_ CMA-0A absolute residuals to less than 3.0 cm^–1^. Overall, if ω_1_ and ω_2_ are removed from statistical consideration in Table 5, the MAE values for all Level B choices range from 0.93 to 0.02 cm^–1^, and the average maximum residual for each Level B is only 1.5 cm^–1^. Thus, CMA-0A yields a sea of very good results encompassing the peculiar (ω_1_, ω_2_) DZ anomalies, and it is only necessary to use a basis set of TZ cardinality to avoid any pitfalls.

**Table 5:** CMA-0A Residuals with Respect to the Reference Harmonic Frequencies (in cm^–1^) for (HCOOH)_2_

	Reference	Level B						Level B				
	CCSD(T)	MP2						CCSD(T)				
	aTZ	DZ	haDZ	aDZ	TZ	haTZ	aTZ	DZ	haDZ	aDZ	TZ	haTZ
ω_1_(*a*_g_)	3190.6	–7.2	–20.4	–38.6	–0.1	–0.1	–0.1	–0.9	–16.4	–5.8	0.0	0.0
ω_2_(*a*_g_)	3098.5	7.4	20.8	39.2	0.1	0.1	0.1	0.8	16.7	5.9	0.0	0.0
ω_3_(*a*_g_)	1708.0	–2.4	–0.8	–0.9	–0.5	–0.3	–0.3	–1.7	–0.3	–0.3	–0.1	0.0
ω_4_(*a*_g_)	1486.8	1.6	0.3	0.4	–0.5	–0.6	–0.4	1.4	0.2	0.3	0.0	0.0
ω_5_(*a*_g_)	1404.2	0.4	0.4	0.3	0.4	0.6	0.5	0.1	–0.1	–0.1	0.0	0.0
ω_6_(*a*_g_)	1250.2	0.9	0.4	0.4	0.8	0.5	0.3	0.7	0.2	0.2	0.1	0.0
ω_7_(*a*_g_)	682.2	0.1	0.1	0.1	0.0	0.0	0.0	0.1	0.0	0.0	0.0	0.0
ω_8_(*a*_g_)	211.1	–0.1	0.1	0.0	–0.3	0.0	0.0	0.1	0.0	–0.1	–0.1	0.0
ω_9_(*a*_g_)	168.2	0.2	0.0	0.1	0.5	0.1	0.1	0.1	0.2	0.3	0.2	0.0
ω_10_(*b*_g_)	1080.2	–0.8	–0.4	–0.3	–0.2	–0.1	–0.1	–0.6	–0.1	–0.1	0.0	0.0
ω_11_(*b*_g_)	963.2	1.0	0.5	0.4	0.3	0.2	0.2	0.6	0.3	0.2	0.1	0.1
ω_12_(*b*_g_)	252.6	0.3	0.0	0.0	0.1	0.0	0.0	0.3	0.0	0.0	0.1	0.0
ω_13_(*a*_u_)	1101.7	–0.9	–0.3	–0.2	–0.1	0.0	–0.1	–0.6	–0.1	0.0	0.0	0.0
ω_14_(*a*_u_)	987.1	1.1	0.4	0.3	0.2	0.1	0.2	0.8	0.2	0.1	0.1	0.1
ω_15_(*a*_u_)	175.9	0.2	0.1	0.1	0.0	0.1	0.0	0.1	0.0	0.1	0.0	0.0
ω_16_(*a*_u_)	69.5	0.1	0.1	0.1	0.1	0.0	0.1	0.2	0.1	0.2	0.2	0.0
ω_17_(*b*_u_)	3294.4	–1.5	–2.8	–2.3	–0.1	–0.1	–0.1	–0.7	–1.4	–1.1	0.0	0.0
ω_18_(*b*_u_)	3094.7	1.5	3.0	2.4	0.1	0.1	0.1	0.7	1.5	1.1	0.0	0.0
ω_19_(*b*_u_)	1772.6	–0.9	–0.4	–0.5	–0.3	–0.2	–0.2	–0.6	–0.1	–0.1	–0.1	0.0
ω_20_(*b*_u_)	1458.2	–2.4	–0.7	–0.3	–1.4	–1.7	–0.9	0.0	0.0	0.1	0.0	–0.1
ω_21_(*b*_u_)	1401.5	3.0	1.0	0.7	1.1	1.6	0.9	0.5	0.0	0.0	0.1	0.1
ω_22_(*b*_u_)	1254.8	0.9	0.4	0.3	0.7	0.6	0.4	0.3	0.1	0.1	0.1	0.0
ω_23_(*b*_u_)	710.8	0.1	0.0	0.0	0.0	0.0	0.0	0.1	0.0	0.0	0.0	0.0
ω_24_(*b*_u_)	277.1	0.1	0.1	0.1	0.1	0.1	0.1	0.2	0.2	0.2	0.1	0.1

The application of CMA-0A to the dispersion-bound benzene···CH_4_ complex (**13**) is summarized in Table 6 for the two Level B choices MP2/TZ and MP2/haTZ. The reference CCSD­(T)/haTZ structure was initially optimized in *C*_3*v*_ symmetry, resulting in a degenerate pair of very small imaginary frequencies (19*i* cm^–1^). Relaxing the geometry to *C*_s_ symmetry yields the new benchmark (**13**) described in Table 2 with a scant 1.2 cm^–1^ lowering of the energy. This *C*_s_ structure still retains a minuscule imaginary frequency ω_45_(*a*″) = 13.0*i* cm^–1^ for further methane reorientations into *C*_1_ symmetry. Locating this final minimum proved computationally intractable, and the whole exercise was somewhat pointless because our CMA methods do not require the reference structure to be a minimum on the PES. Therefore, our *C*_*s*_ structure was adopted for CMA-0A testing on complex **13**.

**Table 6:** CMA-0A Residuals with Respect to the Reference Harmonic Frequencies (in cm^–1^) for Benzene···CH_4_

	Reference	CMA-0A		CMA-1A(3) {5}^a^	CMA-2A(ξ = 0.13)
	CCSD(T)	MP2		MP2	MP2
	haTZ	TZ	haTZ	haTZ	haTZ
ω_1_(*a*′)	3201.3	0.0	0.1	0.1	0.1
ω_2_(*a*′)	3190.7	0.0	0.0	0.0	0.0
ω_3_(*a*′)	3173.0	0.0	0.0	0.0	0.0
ω_4_(*a*′)	3160.5	0.0	0.0	0.0	0.0
ω_5_(*a*′)	3153.7	0.0	–0.1	–0.1	–0.1
ω_6_(*a*′)	3141.5	0.0	0.0	0.0	0.1
ω_7_(*a*′)	3025.0	0.0	0.0	0.0	0.0
ω_8_(*a*′)	1629.7	0.0	0.0	0.0	0.0
ω_9_(*a*′)	1571.1	0.0	0.0	0.0	0.0
ω_10_(*a*′)	1502.8	0.0	0.0	0.0	0.0
ω_11_(*a*′)	1350.1	0.0	0.0	0.0	0.0
ω_12_(*a*′)	1343.8	0.0	0.0	0.0	0.1
ω_13_(*a*′)	1185.8	0.0	0.1	0.1	0.0
ω_14_(*a*′)	1051.4	0.0	0.0	0.0	0.0
ω_15_(*a*′)	1011.1	–0.2	0.0	0.0	0.0
ω_16_(*a*′)	1002.4	0.0	0.0	0.0	0.0
ω_17_(*a*′)	978.0	–**2.5**	–0.1	–0.1	–0.2
ω_18_(*a*′)	975.5	–0.1	0.0	0.0	0.0
ω_19_(*a*′)	855.8	0.0	0.0	0.0	0.0
ω_20_(*a*′)	683.8	**3.8**	0.2	0.2	0.2
ω_21_(*a*′)	682.4	0.0	0.0	0.0	0.0
ω_22_(*a*′)	606.4	0.0	0.0	0.0	0.0
ω_23_(*a*′)	397.9	0.2	0.0	0.0	0.0
ω_24_(*a*′)	89.7	0.0	**–3.3**	–1.3 {0.1}	0.1
ω_25_(*a*′)	63.7	0.1	0.1	0.1 {0.1}	0.1
ω_26_(*a*′)	31.8	0.1	**8.4**	**3.9** {0.1}	0.1
ω_27_(*a*″)	3190.9	0.0	0.0	0.0	0.0
ω_28_(*a*″)	3173.2	0.0	0.0	0.0	0.0
ω_29_(*a*″)	3139.9	0.0	0.0	0.0	0.0
ω_30_(*a*″)	1629.8	0.0	0.0	0.0	0.0
ω_31_(*a*″)	1572.2	0.0	0.0	0.0	0.0
ω_32_(*a*″)	1502.8	0.0	0.0	0.0	0.0
ω_33_(*a*″)	1368.9	0.0	0.0	0.0	0.0
ω_34_(*a*″)	1351.8	0.0	0.0	0.0	0.0
ω_35_(*a*″)	1331.1	–**3.7**	–**4.0**	0.0	0.0
ω_36_(*a*″)	1185.8	0.0	0.0	0.0	0.0
ω_37_(*a*″)	1155.0	**4.2**	**4.6**	0.0	0.0
ω_38_(*a*″)	1051.5	0.0	0.0	0.0	0.0
ω_39_(*a*″)	976.1	–0.1	0.0	0.0	0.0
ω_40_(*a*″)	856.1	0.0	0.0	0.0	0.0
ω_41_(*a*″)	606.4	0.0	0.0	0.0	0.0
ω_42_(*a*″)	397.6	0.2	0.0	0.0	0.0
ω_43_(*a*″)	76.0	–0.5	–0.5	–0.5	0.0
ω_44_(*a*″)	18.7	–1.1	–0.3	–0.3	0.0
ω_45_(*a*″)	13.0*i*	–**6.4** *i*	–**4.0** *i*	–1.0*i*	–0.1*i*

a CMA-1A­(5) residuals given in braces.

Among the 45 vibrational modes of benzene···CH_4_, there are only 5 CMA-0A residuals ≥ 2.5 cm^–1^ for each Level B choice, as highlighted in boldface in Table 6. The benzene ring deformations (ω_35_, ω_37_) display residuals of (−3.7, 4.2) and (−4.0, 4.6) cm^–1^ when B = MP2/TZ and MP2/haTZ, respectively. Similar residuals for these modes occur for isolated benzene when Level B = MP2/TZ targeting Level A = CCSD­(T)/TZ.^16^ The remaining (−2.5, 3.8) cm^–1^ outlier residuals for interfragment vibrations occur for the [(chair) ring-puckering, C–H out-of-plane wagging] modes (ω_17_, ω_20_) when B = MP2/TZ. This same Level B produces residuals of (−3.3, 8.4) cm^–1^ for the diminutive interfragment frequencies (ω_24_, ω_26_) = (89.7, 31.8) cm^–1^. The occurrence of (ω_17_, ω_20_), (ω_24_, ω_26_), and (ω_35_, ω_37_) pairs with opposite signs indicates that CMA-1A and CMA-2A, which are shown in the final two columns of Table 6, should handle all outliers well for the benzene···CH_4_ dimer (*vide infra*). Finally, the characterization of our *C*_s_ symmetry structure as a first-order saddle-point with a tiny imaginary frequency is confirmed by the CMA-0A computations that yield ω_45_(*a*″) = 6.6*i* and 9.0*i* cm^–1^ when Level B = MP2/TZ and MP2/haTZ, respectively. The overall MAE of residuals is (0.52, 0.57) cm^–1^ when Level B = (MP2/TZ, MP2/haTZ), showing that these methods perform equally well for the benzene···CH_4_ system.

The performance of CMA-0A on the mixed-interaction C_2_H_4_···C_2_H_2_ complex (**17**) is highlighted in Table 7. In line with previous results, Level B = MP2/haTZ performs exceptionally well for this system, with no CMA-0A residuals exceeding 0.6 cm^–1^ in magnitude. Additionally, for Level B = MP2/aTZ, MP2/TZ, CCSD­(T)/haDZ, MP2/haDZ, and MP2/DZ no residual magnitudes exceed 2.0 cm^–1^. A single, isolated outlier of 3.8 cm^–1^ arises when Level B = MP2/aDZ, corresponding to an interfragment rotation ω_24_(*b*_2_). The MAEs of all Level B choices are less than 0.5 cm^–1^; MP2/aTZ is the best performer, yielding MAE = 0.03 cm^–1^ and nearly exact results. The success of the frequency computations regardless of Level B for this complex is a powerful demonstration of the CMA-0A method.

**Table 7:** CMA-0A Residuals with Respect to the Reference Harmonic Frequencies (in cm^–1^) for C_2_H_4_···C_2_H_2_

	Reference	Level B						Level B	
	CCSD(T)	MP2						CCSD(T)	
	aTZ	DZ	haDZ	aDZ	TZ	haTZ	aTZ	DZ	haDZ
ω_1_(*a*_1_)	3486.8	–0.4	–0.4	0.0	–0.3	–0.4	–0.1	–0.1	–0.1
ω_2_(*a*_1_)	3375.3	0.3	0.4	0.0	0.3	0.4	0.1	0.1	0.1
ω_3_(*a*_1_)	3149.3	0.0	–0.1	0.0	0.0	0.0	0.0	0.0	–0.1
ω_4_(*a*_1_)	1989.5	0.0	0.0	0.0	0.0	0.0	0.0	0.0	0.0
ω_5_(*a*_1_)	1663.8	–1.3	–1.4	–1.3	0.0	0.0	0.0	–1.1	–1.2
ω_6_(*a*_1_)	1364.4	1.7	1.8	1.7	0.0	0.0	0.0	1.4	1.6
ω_7_(*a*_1_)	967.2	0.0	0.0	0.0	0.0	0.0	0.0	0.0	0.0
ω_8_(*a*_1_)	78.4	0.1	0.9	0.7	0.2	0.2	0.1	0.1	1.7
ω_9_(*a*_2_)	3213.5	0.0	0.0	0.0	0.0	0.0	0.0	0.0	0.0
ω_10_(*a*_2_)	1240.8	0.0	0.0	0.0	0.0	0.0	0.0	0.0	0.0
ω_11_(*a*_2_)	1042.9	0.0	0.0	0.0	0.0	0.0	0.0	0.0	0.0
ω_12_(*b*_1_)	3131.7	0.0	0.0	0.0	0.0	0.0	0.0	0.0	0.0
ω_13_(*b*_1_)	1472.8	0.0	0.0	0.0	0.0	0.0	0.0	0.0	0.0
ω_14_(*b*_1_)	936.4	0.0	0.0	0.0	0.0	0.0	0.0	0.0	0.0
ω_15_(*b*_1_)	775.4	–0.7	–0.5	–0.1	0.0	0.0	0.0	–0.4	–0.5
ω_16_(*b*_1_)	611.9	1.5	0.6	–0.3	0.0	0.1	0.0	0.6	0.6
ω_17_(*b*_1_)	82.3	0.0	–0.8	2.0	–0.3	–0.2	0.0	0.2	–1.5
ω_18_(*b*_1_)	33.6	0.2	0.1	0.3	0.5	0.3	0.0	0.3	0.1
ω_19_(*b*_2_)	3239.9	0.0	0.0	0.0	0.0	0.0	0.0	0.0	0.0
ω_20_(*b*_2_)	817.6	0.1	0.0	0.0	0.0	0.0	0.0	0.0	0.0
ω_21_(*b*_2_)	780.0	–0.5	–0.4	–0.3	–0.3	–0.1	0.0	–0.3	–0.4
ω_22_(*b*_2_)	613.4	0.1	0.5	0.0	0.4	0.1	0.0	0.4	0.5
ω_23_(*b*_2_)	99.4	–0.3	–0.3	0.9	–0.6	–0.2	–0.1	–0.7	0.0
ω_24_(*b*_2_)	35.4	1.2	1.3	3.8	1.8	0.6	0.4	2.3	0.9

## Application of CMA-2A and CMA-1A

While CMA-0A produces highly accurate vibrational frequencies for the intermolecular complexes studied here, the application of CMA-2A promises further improvements in the residual statistics and the elimination of sparse but aggravating outliers. Thus, the entire data set of Level A = CCSD­(T)/aTZ or CCSD­(T)/haTZ frequencies, curated here for species **1**–**17**, was tackled by CMA-2A using the preferred choices Level B = MP2/haTZ and Level C = HF/haTZ for all molecules. To track the systematic convergence to exact Level A frequencies, the CMA-2A procedure was repeated for sequentially smaller values of the ξ cutoff parameter that selects off-diagonal **F**_CMA_(A) elements for explicit computation. Figure 4 provides a cogent representation of the results by plotting the MAE of the residuals of all 435 frequencies in the data set versus the % nonzero off-diagonal elements included in **F**_CMA_(A) for each ξ value chosen in the mapping. The MAE in Figure 4 is rapidly reduced from 0.23 cm^–1^ at CMA-0A to 0.075 cm^–1^ with the inclusion of only 3.0% of the off-diagonal elements. This success is quite astounding, especially considering the challenges posed by the weak binding of the systems studied here. By the time 20% of the off-diagonal elements is introduced, the MAE decreases to 0.026 cm^–1^, almost an order of magnitude smaller than the CMA-0A starting point. Thereafter, the MAE slowly tapers down to 0 cm^–1^ at 100%.

**Figure 4:**
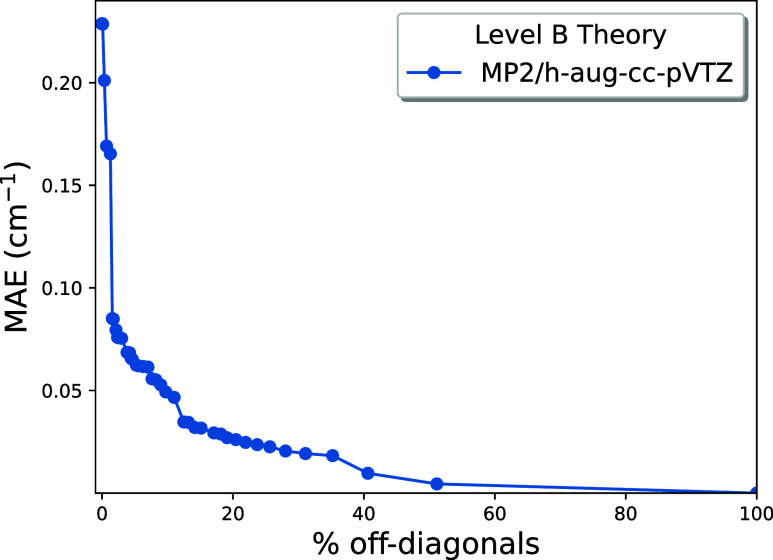
CMA-2A MAE of the CCSD­(T)/aTZ or CCSD­(T)/haTZ benchmark frequencies plotted as a function of % nonzero off-diagonal elements appearing in **F**_CMA_(A).

Special attention was given to the CMA-0A residuals greater than 1.5 cm^–1^ in magnitude, in order to demonstrate that CMA-1A and CMA-2A can eliminate all outliers. All such residuals are collected in Table 8, where 12 cases ranging in magnitude from 3.3 to 8.4 cm^–1^ arise from 4 complexes, all containing benzene. Eight of the 12 outliers involve intrafragment modes of benzene and correspond to a single coupling between ring deformations. This coupling was observed in our previous work^16^ and is completely resolved in all cases by the addition of a single off-diagonal force constant in a CMA-1A treatment. The four outliers involving interfragment modes prove to be a greater challenge for CMA-1A. The inclusion of a single force constant per outlier pair reduces the (ω_20_, ω_45_, ω_24_) residuals to (1.9, −1.0*i*, −1.3) cm^–1^, but such a treatment gives an unsatisfactory 3.9 cm^–1^ residual for ω_26_(*a*′) of the benzene···CH_4_ complex. As seen in Table 6, when CMA-1A(3) is used to fully couple ω_24_(*a*′), ω_25_(*a*′), and ω_26_(*a*′), all the resulting residuals are a minuscule 0.1 cm^–1^.

**Table 8:** Only Cases within the Set of 435 Benchmark Frequencies with CMA-0A­[CCSD­(T)/haTZ, MP2/haTZ] Residuals (*ϵ*, cm^–1^) Greater than 1.5 cm^–1^ in Magnitude, Together with the Corresponding CMA-1A and CMA-2A Results That Target These Outliers

molecule and mode	description	benchmark	ϵ[CMA-0A]	ϵ[CMA-1A]	couplings	ϵ[CMA-2A](*n*)^a^	η^b^ (%)	% off-diagonals	ξ
benzene···CH_4_, ω_26_(*a*′)	interfrag. rot.	31.8	8.4	3.9	(24,26)	0.0(7)	16	1.4	0.13
benzene···HCN, ω_20_(*e*_1_)	interfrag. rot.	14.6	4.2	1.9	(19,20)	0.1(17)	44	9.5	0.04
benzene···CH_4_, ω_45_(*a*″)	interfrag. rot.	13.0*i*	–4.0*i*	–1.0*i*	(43,45)	0.0(7)	16	1.4	0.13
benzene···CH_4_, ω_24_(*a*′)	interfrag. str.	89.7	–3.3	–1.3	(24,26)	0.1(7)	16	1.4	0.13
benzene···H_2_O, ω_18_(*a*)	ring def.	1156.3	–4.7	0.0	(15,18)	0.0(5)	13	0.7	0.18
benzene···CH_4_, ω_37_(*a*″)	ring def.	1155.0	4.6	0.0	(34,36)	0.0(7)	16	1.4	0.13
benzene···NH_3_, ω_34_(*a*″)	ring def.	1155.6	4.6	0.0	(32,34)	0.0(5)	12	1.2	0.18
benzene···HCN, ω_9_(*b*_1_)	ring def.	1157.4	4.5	0.0	(8,9)	0.0(17)	44	9.5	0.04
benzene···H_2_O, ω_15_(*a*)	ring def.	1328.8	–4.1	0.0	(15,18)	0.0(5)	13	0.7	0.18
benzene···CH_4_, ω_35_(*a*″)	ring def.	1331.1	–4.0	0.0	(34,36)	0.0(7)	16	1.4	0.13
benzene···NH_3_, ω_32_(*a*″)	ring def.	1329.8	–4.0	0.0	(32,34)	0.0(5)	12	1.2	0.18
benzene···HCN, ω_8_(*b*_1_)	ring def.	1330.4	–3.9	0.0	(8,9)	0.0(17)	44	9.5	0.04

a *n* = the number of off-diagonal elements included for the specified ξ cutoff.

b η = number of **F**_CMA_(A) off-diagonal elements included as a percentage of the vibrational degrees of freedom for the given molecule.

While CMA-1A exhibits superb performance in eliminating outliers with optimal efficiency, this method requires the manual selection of off-diagonal elements; thus, assessment of the automated CMA-2A approach is warranted. CMA-2A was tested on the largest residuals with Level C = HF/haTZ so that no further computations are required, since Level B = MP2/haTZ single-point energies have already been computed. A set of optimally performing ξ values (0.04–0.18) were utilized, as shown in the final column of Table 8. Spectacularly, all 12 of the outliers are eliminated by CMA-2A (|ϵ| ≤ 0.1 cm^–1^). At most, only 17 extra force constants were introduced, corresponding roughly to a 12–44% increase in computations relative to CMA-0A. This CMA-2A performance is remarkable considering that CMA-0A already reduces the cost of Level A frequency computations by 700–1000%.^15^ The full CMA-2A (ξ = 0.13) results for the benzene···CH_4_ complex are shown in the final column of Table 6 where all residuals are reduced below 0.2 cm^–1^, thus definitively capturing the Level A frequencies.

## Summary

The Level A benchmark structures and energetics computed in this work agree with, upgrade, or correct the best available literature values. A remarkable finding is that CCSD­(T)/aTZ yields an anomalous, nonplanar benzene framework for the monomer and its complexes due to insidious near linear dependencies in the basis set. This problem is fixed by using Level A = CCSD­(T)/haTZ for benzene-containing complexes. For the four benzene heterodimers containing methane, hydrogen cyanide, water, and ammonia, our CCSD­(T)/haTZ results are now the best fully optimized structures and vibrationless BEs. At Level A = CCSD­(T)/aTZ, the fully optimized nitrosomethane-ammonia, acetic acid-water, ethylene dimer, and ethylene-acetylene complexes computed here also have the highest level of theory structures and vibrationless BEs. To settle disagreements between our CCSD­(T)/aTZ structures and prior literature, the (formamide dimer, acetylene-water) complexes were fully optimized with [CCSD­(T)/aQZ, CCSD­(T)/a5Z], yielding definitive structures and binding energies.

In congruence with prior CMA benchmarking,^15,16^ it was determined that when targeting CCSD­(T)/aTZ frequencies with CMA-0A for our selected dimer systems, MP2/haTZ is the preferred Level B method. This Level B reproduced 435 benchmark frequencies with an MAE of 0.23 cm^–1^ and a corresponding σ_ϵ_ of 0.84 cm^–1^. The choice of basis set is of fundamental importance for hydrogen-bonded, dispersion, and mixed complexes, as highlighted by the water, formic acid, benzene-methane, and ethylene-acetylene dimers. A linear algebra theorem was derived proving that for any symmetric, real-valued matrix with all positive diagonal elements and eigenvalues, the sum of the square roots of the diagonal values will always be greater than or equal to the sum of the square roots of the eigenvalues. Application of this theorem to the CMA-0A method substantiates that the associated ZPVE will always be greater than or equal to the Level A ZPVE.

The manual CMA-1A and automated CMA-2A schemes for sparse off-diagonal force constant computation beyond CMA-0A significantly reduce the residual frequency errors for our selected dimer systems. For the set of CMA-0A frequencies with Level B = MP2/haTZ, CMA-2A reduces the MAE of residuals from 0.23 cm^–1^ to 0.08 cm^–1^ by including only 3.0% of the off-diagonal force constants. Twelve outlier residuals in the range of 3.3–8.4 cm^–1^ were found to be corrected by CMA-1A and CMA-2A. Eleven of the 12 residuals are reduced below 2 cm^–1^ via CMA-1A by including only one off-diagonal force constant per pair of outliers, and the remaining residual is nullified by the addition of just two more off-diagonal force constants. CMA-2A proves to be exceptionally powerful, as all 12 residuals are corrected with only a 12–44% increase in computations relative to CMA-0A. Overall, the CMA hierarchy continues to exhibit exceptional performance for these dimer systems and thus handles the diversification into intermolecular vibrations with ease.

## Supplementary Material





## Notes

xyz structures (ZIP)
Basis set definitions, interfragment internal coordinate definitions, proof concerning ZPVEs of CMA-0A, benzene monomer frequencies, and CMA dimer frequencies (PDF)
