# Supplementary material for: Concordant Mode Approach (CMA): Vibrational Analysis of New and Upgraded Intermolecular Benchmarks for Noncovalent Bonding
Source: J Phys Chem A. 2026 Apr 13;130(16):3249–60. doi: 10.1021/acs.jpca.6c00689 (PMC13112346; doi:10.1021/acs.jpca.6c00689)
Supplement: Supplementary file 2 [file jp6c00689_si_002.pdf]

Supporting Information for Concordant Mode Approach (CMA) Vibrational Analysis of New and Upgraded Intermolecular Benchmarks for Noncovalent Bonding

Laura N. Olive Dornshuld[1], Mitchell E. Lahm[1], Nathaniel L. Kitzmiller[1,2], Wesley D. Allen[1,3], and Henry F. Schaefer III[1]

[1]Center for Computational Quantum Chemistry, University of Georgia, Athens, GA 30602 USA
[2]Indiana Wesleyan University, Marion, Indiana 46953, USA
[3]Allen Heritage Foundation, Dickson, Tennessee 37055, USA

Contents

S1 Definitions and Proofs **S3**

S1.1 Basis Set Definitions S3

S1.2 Interfragment Internal Coordinate Definitions S3

S1.3 Proof Concerning ZPVEs of CMA-0A S3

S2 Monomer Frequencies **S6**

S2.1 benzene S6

S3 CMA Dimer Frequencies **S7**

S3.1 water dimer S7

S3.2 ammonia dimer S10

S3.3 water – ammonia dimer S14

S3.4 formic acid dimer S17

S3.5 nitrosomethane – ammonia dimer S21

S3.6 formamide dimer S24

S3.7 water – acetylene dimer S29

S3.8 water – acetic acid dimer S32

S3.9 hydrofluoric acid dimer S36

S3.10 water trimer S39

S3.11 ethylene dimer S45

S3.12 methane dimer S49

S3.13 benzene – methane dimer S53

S3.14 benzene – hydrogen cyanide dimer S57

S3.15 benzene – water dimer S61

S3.16 benzene – ammonia dimer S65

S3.17 ethylene – acetylene dimer S69

S1 Definitions and Proofs

S1.1 Basis Set Definitions

Throughout this Supporting Information, the basis sets cc-pVXZ, h-aug-cc-pVXZ, aug-cc-pVXZ are denoted as XZ, ha$X$Z, and aXZ, respectively, where X is the cardinality of the basis set.

S1.2 Interfragment Internal Coordinate Definitions

As discussed in Section 4 of the text, some special interfragment internal coordinates were utilized that employ generalized reference points in each monomer. In this work centroids were often used as these reference points. In our notation, when entries separated by vertical bars are listed as multiple values separated by commas, it means that a centroid of the listed atomic centers is used for that point of the respective internal coordinate. For example, the $r(1,2,3|4,5,6)$ coordinate is a distance between the centroids of atoms (1, 2, 3) and (4, 5, 6), respectively. Likewise, $\theta(1|2|3,4)$ is the angle between points 1, 2, and the centroid of (3, 4).

S1.3 Proof Concerning ZPVEs of CMA-0A

Consider any symmetric matrix $\mathbf{A}$ whose diagonal elements and eigenvalues are all positive. Here we prove that the sum of the square roots of its eigenvalues is less than or equal to the sum of the square roots of its diagonal elements. In benchmarking the CMA method, a force constant matrix in the basis of level B normal modes, $\mathbf{F}_{\mathrm{CMA}}$, is constructed. $\mathbf{F}_{\mathrm{CMA}}$ is semi-diagonal so as an approximation the off-diagonal elements can be neglected. This approximation is referred to as CMA-0. Furthermore, if the CMA-0A variant is employed the geometry on which the level B normal modes and level A force constants are computed will stay constant at level A. Consequently, the G-matrix analogue to $\mathbf{F}_{\mathrm{CMA}}$, $\mathbf{G}_{\mathrm{CMA}}$, will be strictly diagonal and only ever a weighting to $\mathbf{F}_{\mathrm{CMA}}$ in the GF-matrix method. Thus, it is only relevant for the purposes of comparing zero-point vibrational energies (ZPVEs) between these methods to consider the sum of square roots of the *diagonal elements* and *eigenvalues* of $\mathbf{F}_{\mathrm{CMA}}$.

Start with $\mathbf{A} = \mathbf{A}^{(0)}$ and execute a series of Jacobi rotations until $\mathbf{A}$ becomes diagonal. Suppose the matrix $\mathbf{A}^{(p-1)}$ at iteration $(p-1)$ is subjected to a Jacobi rotation targeting elements $A_{mn}^{(p-1)} = A_{nm}^{(p-1)}$. The unitary transformation yields

$$A_{ij}^{(p)} = \sum_{kl} U_{ki} A_{kl}^{(p-1)} U_{lj} \tag{1}$$

where the only nonzero, off-diagonal elements of $\mathbf{U}$ are $U_{mn} = \sin\theta$ and $U_{nm} = -\sin\theta$, while the only diagonal elements of $\mathbf{U}$ not equal to 1 are $U_{mm} = \cos\theta$ and $U_{nn} = \cos\theta$. Accordingly, $A_{ij}^{(p)} = A_{ij}^{(p-1)}$ if neither i nor j equals m and neither i nor j equals n. Otherwise, eq 1 yields

$$A_{mm}^{(p)} = A_{mm}^{(p-1)} \cos^2\theta + A_{nn}^{(p-1)} \sin^2\theta - 2A_{mn}^{(p-1)} \cos\theta \sin\theta \tag{2}$$

$$A_{nn}^{(p)} = A_{nn}^{(p-1)} \cos^2\theta + A_{mm}^{(p-1)} \sin^2\theta + 2A_{mn}^{(p-1)} \cos\theta \sin\theta \tag{3}$$

$$A_{mn}^{(p)} = (A_{mm}^{(p-1)} - A_{nn}^{(p-1)}) \cos\theta \sin\theta + A_{mn}^{(p-1)} (\cos^2\theta - \sin^2\theta) \tag{4}$$

$$A_{mj}^{(p)} = \cos\theta A_{mj}^{(p-1)} - \sin\theta A_{nj}^{(p-1)} \quad (j \neq m,n) \tag{5}$$

$$A_{nj}^{(p)} = \cos\theta A_{nj}^{(p-1)} + \sin\theta A_{mj}^{(p-1)} \quad (j \neq m,n) \tag{6}$$

Setting $A_{mn}^{(p)} = 0$ in eq 4 gives

$$\rho = \frac{2A_{mn}^{(p-1)}}{A_{nn}^{(p-1)} - A_{mm}^{(p-1)}} = \tan 2\theta \tag{7}$$

or equivalently

$$\sin 2\theta = \frac{\rho}{\sqrt{\rho^2+1}} \tag{8}$$

$$\cos 2\theta = \frac{1}{\sqrt{\rho^2+1}} \tag{9}$$

$$2\sin^2\theta = 1 - \frac{1}{\sqrt{\rho^2+1}} \tag{10}$$

Employing eqs 5 and 6 with the condition $A_{mn}^{(p)} = 0$, we find

$$\sum_{j(\neq m)} [A_{mj}^{(p)}]^2 = \sum_{j(\neq m,n)} \{\cos^2\theta[A_{mj}^{(p-1)}]^2 + \sin^2\theta[A_{nj}^{(p-1)}]^2 - 2\sin\theta\cos\theta A_{mj}^{(p-1)}A_{nj}^{(p-1)}\} \tag{11}$$

$$\sum_{j(\neq n)} [A_{nj}^{(p)}]^2 = \sum_{j(\neq m,n)} \{\cos^2\theta[A_{nj}^{(p-1)}]^2 + \sin^2\theta[A_{mj}^{(p-1)}]^2 + 2\sin\theta\cos\theta A_{mj}^{(p-1)}A_{nj}^{(p-1)}\} \tag{12}$$

Therefore, the sum of the squares of the off-diagonal elements, $Q^{(p)}$, satisfies

$$Q^{(p)} = 2\sum_{j(\neq m,n)} \{[A_{nj}^{(p-1)}]^2 + [A_{mj}^{(p-1)}]^2\} = Q^{(p-1)} - 4[A_{mn}^{(p-1)}]^2, \tag{13}$$

so that $Q^{(p)}$ monotonically decreases during the Jacobi iterations, ultimately leading to a converged diagonal matrix.

Let us now return to eq 2 and use eqs 8 and 9 to obtain a final form.

$$A_{mm}^{(p)} = \frac{1}{2}[A_{mm}^{(p-1)} - A_{nn}^{(p-1)}](1+\cos 2\theta) + A_{nn}^{(p-1)} - A_{mn}^{(p-1)}\sin 2\theta \Longrightarrow$$

$$A_{mm}^{(p)} = \frac{1}{2}[A_{mm}^{(p-1)} - A_{nn}^{(p-1)}](1+\frac{1}{\sqrt{\rho^2+1}}) + A_{nn}^{(p-1)} - A_{mn}^{(p-1)}\frac{\rho}{\sqrt{\rho^2+1}} \Longrightarrow$$

$$2A_{mm}^{(p)} = A_{mm}^{(p-1)} + A_{nn}^{(p-1)} + \{A_{mm}^{(p-1)} - A_{nn}^{(p-1)} - 2\rho A_{mn}^{(p-1)}\}(\frac{1}{\sqrt{\rho^2+1}}) \Longrightarrow$$

$$2A_{mm}^{(p)} = A_{mm}^{(p-1)} + A_{nn}^{(p-1)} + \{A_{mm}^{(p-1)} - A_{nn}^{(p-1)} - \rho^2[A_{nn}^{(p-1)} - A_{mm}^{(p-1)}]\}(\frac{1}{\sqrt{\rho^2+1}}) \Longrightarrow$$

$$2A_{mm}^{(p)} = A_{mm}^{(p-1)} + A_{nn}^{(p-1)} + [A_{mm}^{(p-1)} - A_{nn}^{(p-1)}]\{1+\rho^2\}(\frac{1}{\sqrt{\rho^2+1}}) \Longrightarrow$$

$$2A_{mm}^{(p)} = A_{mm}^{(p-1)} + A_{nn}^{(p-1)} + [A_{mm}^{(p-1)} - A_{nn}^{(p-1)}]\sqrt{\rho^2+1}. \tag{14}$$

Likewise,

$$2A_{nn}^{(p)} = A_{mm}^{(p-1)} + A_{nn}^{(p-1)} - [A_{nn}^{(p-1)} - A_{mm}^{(p-1)}]\sqrt{\rho^2+1}. \tag{15}$$

Note that eqs 14 and 15 provide $A_{mm}^{(p)} + A_{nn}^{(p)} = A_{mm}^{(p-1)} + A_{nn}^{(p-1)}$, consistent with the invariance of the trace under a unitary transformation.

Now we can calculate

$$S^{(p)} = \sqrt{A_{mm}^{(p)}} + \sqrt{A_{nn}^{(p)}}, \tag{16}$$

for which

$$[S^{(p)}]^2 - [S^{(p-1)}]^2 = A_{mm}^{(p)} + A_{nn}^{(p)} + 2\sqrt{A_{mm}^{(p)}A_{nn}^{(p)}} - A_{mm}^{(p-1)} - A_{nn}^{(p-1)} - 2\sqrt{A_{mm}^{(p-1)}A_{nn}^{(p-1)}} \Longrightarrow$$

$$[S^{(p)}]^2 - [S^{(p-1)}]^2 = \sqrt{4A_{mm}^{(p)}A_{nn}^{(p)}} - \sqrt{4A_{mm}^{(p-1)}A_{nn}^{(p-1)}} \Longrightarrow$$

$$[S^{(p)}]^2 - [S^{(p-1)}]^2 = \sqrt{4A_{mm}^{(p-1)}A_{nn}^{(p-1)}}\left[\sqrt{\frac{A_{mm}^{(p)}A_{nn}^{(p)}}{A_{mm}^{(p-1)}A_{nn}^{(p-1)}}} - 1\right] \tag{17}$$

However, from eqs 14 and 15,

$$4A_{mm}^{(p)}A_{nn}^{(p)} = [A_{mm}^{(p-1)} + A_{nn}^{(p-1)}]^2 - [A_{mm}^{(p-1)} - A_{nn}^{(p-1)}]^2(\rho^2+1) \Longrightarrow$$

$$4A_{mm}^{(p)}A_{nn}^{(p)} = 4A_{mm}^{(p-1)}A_{nn}^{(p-1)} - \rho^2[A_{mm}^{(p-1)} - A_{nn}^{(p-1)}]^2 \Longrightarrow$$

$$\frac{A_{mm}^{(p)}A_{nn}^{(p)}}{A_{mm}^{(p-1)}A_{nn}^{(p-1)}} = 1 - \rho^2\frac{[A_{mm}^{(p-1)} + A_{nn}^{(p-1)}]^2}{4A_{mm}^{(p-1)}A_{nn}^{(p-1)}} = 1 - \frac{[A_{mn}^{(p-1)}]^2}{A_{mm}^{(p-1)}A_{nn}^{(p-1)}} \tag{18}$$

Utilizing eq 18 in eq 17 yields

$$[S^{(p)}]^2 - [S^{(p-1)}]^2 = -\sqrt{4A_{mm}^{(p-1)}A_{nn}^{(p-1)}}\left[1 - \sqrt{1 - \frac{[A_{mn}^{(p-1)}]^2}{A_{mm}^{(p-1)}A_{nn}^{(p-1)}}}\right], \tag{19}$$

which proves that $S^{(p)}$ always decreases during the Jacobi iterations if two conditions are met: (a) all diagonal elements remain positive, and (b) $[A_{mn}^{(p-1)}]^2 < A_{mm}^{(p-1)}A_{nn}^{(p-1)}$. In other words, our theorem of concern has been proved if conditions (a) and (b) are met.

Condition (a)
Eqs 14 and 15 can be re-written as

$$A_{mm}^{(p)} = \frac{1}{2}A_{mm}^{(p-1)}(1+\sqrt{\rho^2+1}) + \frac{1}{2}A_{nn}^{(p-1)}(1-\sqrt{\rho^2+1}) \tag{20}$$

$$A_{nn}^{(p)} = \frac{1}{2}A_{mm}^{(p-1)}(1-\sqrt{\rho^2+1}) + \frac{1}{2}A_{nn}^{(p-1)}(1+\sqrt{\rho^2+1}) \tag{21}$$

From eqs 20 and 21 and the invariance of the trace, we can deduce the following outcomes regarding the signs of the diagonal elements resulting from a Jacobi rotation.

$$\begin{aligned}(\textit{two positives}) &\rightarrow (\textit{two positives})\ \textit{OR}\ (\textit{one positive}, \textit{one negative})\\ (\textit{one positive}, \textit{one negative}) &\rightarrow (\textit{one positive}, \textit{one negative})\\ (\textit{two negatives}) &\rightarrow (\textit{two negatives})\ \textit{OR}\ (\textit{one positive}, \textit{one negative})\end{aligned}$$

Before the Jacobi iterations commence, all the diagonal elements of **A** are assumed positive. Suppose at some point in the iterations, one or more of the diagonal elements becomes negative. The outcome possibilites then provide no means of making *all* diagonal elements positive again. Therefore, at least one negative eigenvalue of **A** will appear, inconsistent with the premise of our theorem. In other words, condition (a) must hold if all eigenvalues of **A** are positive.

Condition (b)
Suppose $[A_{mn}^{(p-1)}]^2 = \frac{1}{4}[A_{mm}^{(p-1)} - A_{nn}^{(p-1)}]^2\rho^2 > A_{mm}^{(p-1)}A_{nn}^{(p-1)}$ while both diagonal elements are positive. Then

$$\rho^2 + 1 > \frac{4A_{mm}^{(p-1)}A_{nn}^{(p-1)}}{[A_{mm}^{(p-1)} - A_{nn}^{(p-1)}]^2} + 1 = \frac{[A_{mm}^{(p-1)} - A_{nn}^{(p-1)}]^2 + 4A_{mm}^{(p-1)}A_{nn}^{(p-1)}}{[A_{mm}^{(p-1)} - A_{nn}^{(p-1)}]^2} \Longrightarrow$$

$$\rho^2 + 1 > \frac{[A_{mm}^{(p-1)} + A_{nn}^{(p-1)}]^2}{[A_{mm}^{(p-1)} - A_{nn}^{(p-1)}]^2} \Longrightarrow$$

$$|A_{mm}^{(p-1)} - A_{nn}^{(p-1)}|\sqrt{\rho^2+1} > A_{mm}^{(p-1)} + A_{nn}^{(p-1)}. \tag{22}$$

Now consider the consequences of eq 22 in eqs 14 and 15. We see that if $A_{mm}^{(p-1)} > A_{nn}^{(p-1)}$, then $A_{nn}^{(p)} < 0$. On the other hand, if $A_{mm}^{(p-1)} < A_{nn}^{(p-1)}$, then $A_{mm}^{(p)} < 0$. In either case a diagonal element has become negative in the Jacobi iteration, which from our considerations from condition (a) means that the eigenvalues of **A** cannot all be positive. Therefore, we deduce by contradiction that $[A_{mn}^{(p-1)}]^2 < A_{mm}^{(p-1)}A_{nn}^{(p-1)}$ and that condition (b) holds from the premise of our theorem.

In summary, eq 19 reveals that $S^{(p)} < S^{(p-1)}$ for every Jacobi iteration in the sequence that diagonalizes **A**. These quantities involve the only diagonal elements that change in a single Jacobi iteration. Thus, the overall sum of the square roots of the diagonal elements of $\mathbf{A}^{(p-1)}$ must decrease in each Jacobi step. Ultimately, we arrive at the sum of the square roots of the eigenvalues of **A**, which is the limit of a monotonically decreasing series starting at the sum of the square roots of the original diagonal elements of **A**. This non-broken connection proves the desired theorem.

S2 Monomer Frequencies

S2.1 benzene

Table S1: Estimated experimental harmonic frequencies (in cm^{-1}) compared to CCSD(T)/h-aug-cc-pVTZ and CCSD(T)/cc-pVTZ harmonic frequencies for D_{6h} benzene with exceptional variations for the puckering mode in bold.

	Experiment[1]	CCSD(T)/ h-aug-cc-pVTZ[a]	CCSD(T)/ cc-pVTZ[a]
ω_1 (a_{1g})	3191	3201	3209
ω_2 (a_{1g})	1008	1002	1005
ω_3 (a_{2g})	1367	1370	1370
ω_4 (a_{2u})	686	683	685
ω_5 (b_{1u})	3174	3160	3169
ω_6 (b_{1u})	1024	1009	1010
ω_7 (b_{2g})	990	976	967
ω_8 (b_{2g})	**718**	**646**	**675**
ω_9 (b_{2u})	1318	1330	1328
ω_{10}(b_{2u})	1167	1155	1159
ω_{11}(e_{1g})	847	855	856
ω_{12}(e_{1u})	3181	3191	3198
ω_{13}(e_{1u})	1494	1503	1507
ω_{14}(e_{1u})	1058	1051	1054
ω_{15}(e_{2g})	3174	3173	3181
ω_{16}(e_{2g})	1607	1630	1637
ω_{17}(e_{2g})	1178	1186	1191
ω_{18}(e_{2g})	613	607	607
ω_{19}(e_{2u})	967	977	959
ω_{20}(e_{2u})	407	395	401

[a] Computed in this work

S3 CMA Dimer Frequencies

S3.1 water dimer

Structure

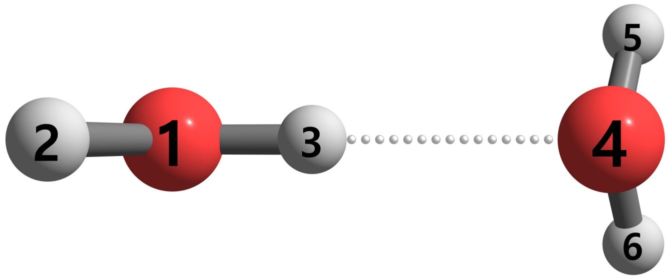

Figure S1: Water dimer structure labeled with atomic indices.

Literature Comparison

Table S2: A comparison of key geometric features to the highest available level of theory Literature geometric structure. Units are either Å or °, depending on the coordinate type.

Geometric Parameter	This work CCSD(T)/aug-cc-pVTZ	Lane CCSDT(Q)/CBS[2]	(Q)/CBS Difference
$r_{O_4H_5}$	0.9624	0.9584	−0.0040
$r_{O_1H_3}$	0.9677	0.9641	−0.0036
$r_{O_1H_2}$	0.9608	0.9569	−0.0039
$\theta_{H_2O_1H_3}$	104.53	104.85	0.32
$\theta_{H_5O_4H_6}$	104.58	104.95	0.36
$r_{O_4\cdots H_3}$	1.9527	1.9521	−0.0005
$\theta_{O_1H_3\cdots O_4}$	171.57	171.51	−0.06
$\gamma_{H_3\cdots O_4H_5H_6}$	55.37	53.74	−1.63

Frequencies

Table S3: CMA-0A residuals with respect to the reference harmonic frequencies (in cm^{-1}) for $(H_2O)_2$ using natural internal coordinates with cartesian force constants.

	Reference	CMA-0A										
	CCSD(T)	MP2						CCSD(T)				
	aTZ	DZ	haDZ	aDZ	TZ	haTZ	aTZ	DZ	haDZ	aDZ	TZ	haTZ
$\omega_1(a')$	3891.4	−1.1	−0.1	−0.1	−0.1	0.0	0.0	−1.7	−0.1	−0.1	−0.1	0.0
$\omega_2(a')$	3805.5	−0.1	0.0	0.0	0.0	0.0	0.0	0.0	0.0	0.0	0.0	0.0
$\omega_3(a')$	3731.1	1.2	0.0	0.1	0.1	0.0	0.0	1.8	0.0	0.1	0.1	0.0
$\omega_4(a')$	1667.5	−0.1	0.0	0.0	0.0	0.0	0.0	−0.1	0.0	0.1	0.0	0.0
$\omega_5(a')$	1646.3	0.1	0.0	0.0	0.1	0.0	0.0	0.1	0.0	0.0	0.1	0.1
$\omega_6(a')$	359.8	−0.2	−0.7	−0.3	0.0	0.0	0.0	−0.3	−0.8	−0.3	0.0	0.0
$\omega_7(a')$	185.1	−5.8	−0.1	0.0	−2.6	0.0	−0.2	−5.3	0.5	−0.4	−2.5	−0.1
$\omega_8(a')$	153.5	24.3	2.0	0.4	−0.4	0.0	0.3	25.7	1.3	−1.2	2.1	0.1
$\omega_9(a'')$	3911.1	0.0	0.0	0.0	0.0	0.0	0.0	0.1	0.1	0.0	0.0	0.0
$\omega_{10}(a'')$	621.8	−9.4	−0.4	0.2	−2.0	0.3	0.3	−9.0	−0.2	0.2	−2.0	0.2
$\omega_{11}(a'')$	143.4	17.0	1.9	1.0	7.1	0.1	0.5	14.0	2.8	3.2	7.1	0.0
$\omega_{12}(a'')$	130.0	2.0	1.0	0.1	6.8	0.5	0.1	5.0	1.3	0.1	3.9	0.7

Table S4: Pure level B residuals with respect to the reference harmonic frequencies (in cm^{-1}) for $(H_2O)_2$ using natural internal coordinates with cartesian force constants.

	Reference	Pure										
	CCSD(T)	MP2						CCSD(T)				
	aTZ	DZ	haDZ	aDZ	TZ	haTZ	aTZ	DZ	haDZ	aDZ	TZ	haTZ
$\omega_1(a')$	3891.4	97.88	77.59	70.27	20.41	24.86	20.92	78.54	57.72	50.19	−1.19	4.08
$\omega_2(a')$	3805.5	81.86	56.16	45.73	9.12	8.20	6.40	73.05	49.88	39.40	1.23	1.76
$\omega_3(a')$	3731.1	86.21	47.27	40.01	5.64	0.92	−2.74	86.60	49.86	42.92	6.44	3.74
$\omega_4(a')$	1667.5	13.20	−0.61	−16.20	−7.52	15.15	−17.62	28.24	16.50	0.61	10.30	2.73
$\omega_5(a')$	1646.3	6.92	−2.01	−16.06	−9.04	17.00	−18.22	22.32	15.39	1.40	8.92	1.41
$\omega_6(a')$	359.8	8.21	14.17	0.70	−0.78	−0.63	2.13	7.36	12.52	−0.97	−1.94	−2.59
$\omega_7(a')$	185.1	1.48	8.45	−0.84	−5.01	−1.81	−2.31	5.85	10.58	2.62	−1.82	0.89
$\omega_8(a')$	153.5	−25.80	8.49	−4.41	−18.95	−1.47	0.65	−22.57	9.77	−5.86	−18.32	−2.60
$\omega_9(a'')$	3911.1	110.36	80.53	73.06	25.87	26.89	23.88	89.77	59.04	51.27	2.44	4.31
$\omega_{10}(a'')$	621.8	−48.07	8.02	24.83	−32.48	5.55	9.21	−55.51	−1.88	14.75	−41.38	−5.30
$\omega_{11}(a'')$	143.4	−7.73	11.50	9.89	−10.58	3.86	3.92	−9.04	8.30	5.46	−11.86	0.16
$\omega_{12}(a'')$	130.0	−235.77	6.82	2.40	−90.16	−0.73	0.69	−232.53	6.69	3.35	−91.30	−0.44

Natural Internal Coordinates

Table S5: Symmetrized, unnormalized natural internal coordinates for water.

1	$r_{1,2} + r_{1,3}$
2	$r_{1,2} - r_{1,3}$
3	$r_{4,5} + r_{4,6}$
4	$r_{4,5} - r_{4,6}$
5	$r_{3,4}$
6	$\phi_{5,4,6}$
7	$\phi_{2,1,3}$
8	$\phi_{1,3,4}$
9	$\phi(3\vert 4\vert 5,6)$
10	$\tau(1\vert 3\vert 4\vert 5,6)$
11	$\tau(2,3\vert 1\vert 3\vert 4)$
12	$\tau(5\vert 5,6\vert 4\vert 3) + \tau(6\vert 5,6\vert 4\vert 3)$

S3.2 ammonia dimer

Structure

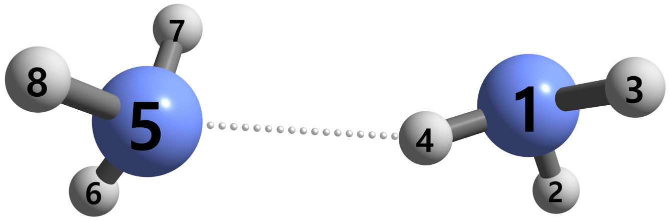

Figure S2: Ammonia dimer structure labeled with atomic indices.

Literature Comparison

Table S6: A comparison of key geometric features to the highest available level of theory Literature geometric structure. Units are either Å or °, depending on the coordinate type.

Geometric Parameter	This work CCSD(T)/aug-cc-pVTZ	Mella MP2/aTZ[3]	Difference
$r_{N_1H_4}$	1.0183	1.0146	−0.0037
$r_{N_1H_2}$	1.0150	1.0102	−0.0048
$r_{N_5H_7}$	1.0157	1.0112	−0.0045
$r_{N_5H_6}$	1.0150	1.0104	−0.0046
$\theta_{H_2N_1H_3}$	106.13	106.60	0.47
$\theta_{H_4N_1H_3}$	106.84	107.33	0.51
$\theta_{H_6N_5H_8}$	106.43	106.85	0.42
$\theta_{H_7N_5H_8}$	106.51	106.95	0.44
$r_{N_5\cdots H_4}$	2.2902	2.2879	−0.0023
$\theta_{N_1H_4\cdots N_5}$	156.65	155.46	−1.19
$\theta_{H_7N_5\cdots H_4}$	85.18	83.56	−1.62

Frequencies

Table S7: CMA-0A residuals with respect to the reference harmonic frequencies (in cm^{-1}) for $(NH_3)_2$ using natural internal coordinates with cartesian force constants.

	Reference	CMA-0A										
	CCSD(T)	MP2						CCSD(T)				
	aTZ	DZ	haDZ	aDZ	TZ	haTZ	aTZ	DZ	haDZ	aDZ	TZ	haTZ
$\omega_1(a')$	3587.5	−0.2	0.0	0.0	−0.2	−0.3	0.0	−0.2	−0.4	0.0	−0.1	−0.1
$\omega_2(a')$	3562.8	−0.3	−0.1	−0.1	0.0	−0.1	−0.1	−0.7	−0.1	−0.1	−0.1	−0.1
$\omega_3(a')$	3461.1	−0.1	0.0	0.0	0.0	0.0	0.0	0.0	0.0	0.0	0.0	0.0
$\omega_4(a')$	3438.5	0.2	0.0	0.0	0.0	0.1	0.1	0.7	0.0	0.1	0.0	0.0
$\omega_5(a')$	1677.9	0.3	0.3	0.3	0.4	0.4	0.2	0.3	0.3	0.4	0.3	0.6
$\omega_6(a')$	1665.0	−0.3	−0.3	−0.3	−0.3	−0.4	−0.2	−0.3	−0.3	−0.3	−0.4	−0.5
$\omega_7(a')$	1094.3	−2.5	0.1	−0.1	−0.7	0.0	0.0	−2.5	−0.1	−0.3	−0.4	0.0
$\omega_8(a')$	1083.0	2.8	0.1	0.2	0.8	0.0	0.0	2.7	0.1	0.3	0.5	0.0
$\omega_9(a')$	381.9	0.4	−0.1	0.2	0.1	0.0	0.1	0.1	−0.1	0.0	0.0	0.0
$\omega_{10}(a')$	141.1	0.1	0.0	−0.4	0.0	0.1	0.0	0.1	0.0	−0.3	0.0	0.1
$\omega_{11}(a')$	60.4	0.5	0.8	1.5	0.3	0.1	0.1	0.6	1.4	1.0	0.3	0.3
$\omega_{12}(a'')$	3591.3	0.0	−0.1	−0.5	0.1	0.5	0.0	0.0	0.7	−1.0	0.2	0.1
$\omega_{13}(a'')$	3587.7	0.1	0.2	0.6	0.2	−0.2	0.1	0.2	−0.2	1.0	0.0	0.1
$\omega_{14}(a'')$	1696.7	0.1	0.1	0.1	0.0	0.0	0.0	0.0	0.0	0.0	0.0	0.0
$\omega_{15}(a'')$	1674.1	0.0	0.0	0.1	0.0	0.0	0.0	0.1	0.0	0.0	0.0	0.0
$\omega_{16}(a'')$	238.1	0.1	0.0	0.0	0.2	0.0	0.0	0.1	0.0	−0.2	0.1	0.0
$\omega_{17}(a'')$	105.0	−0.1	0.2	0.2	0.1	0.1	0.1	−0.1	0.2	0.4	0.1	0.1
$\omega_{18}(a'')$	42.8	1.5	0.4	0.4	0.5	0.3	0.3	1.3	0.4	0.6	0.5	0.2

Table S8: Pure level B residuals with respect to the reference harmonic frequencies (in cm^{-1}) for $(NH_3)_2$ using natural internal coordinates with cartesian force constants.

	Reference	Pure										
	CCSD(T)	MP2						CCSD(T)				
	aTZ	DZ	haDZ	aDZ	TZ	haTZ	aTZ	DZ	haDZ	aDZ	TZ	haTZ
$\omega_1(a')$	3587.5	144.21	108.84	98.63	24.60	25.13	24.10	124.47	88.45	77.18	77.18	0.90
$\omega_2(a')$	3562.8	138.90	104.37	99.13	18.31	19.88	18.96	124.70	88.80	82.73	82.73	0.77
$\omega_3(a')$	3461.1	111.22	83.76	71.98	8.08	8.91	9.26	102.40	76.99	63.76	63.76	−0.42
$\omega_4(a')$	3438.5	107.80	79.31	73.38	3.96	4.44	5.15	102.41	76.32	69.12	69.12	−0.79
$\omega_5(a')$	1677.9	−1.90	6.67	−10.30	−4.85	−5.61	−6.98	0.91	13.55	−3.60	−3.60	1.31
$\omega_6(a')$	1665.0	−2.77	6.08	−11.69	−5.95	−5.86	−7.33	0.42	13.39	−4.48	−4.48	1.41
$\omega_7(a')$	1094.3	−16.79	15.33	−6.74	−7.96	−8.32	−10.83	−10.88	25.07	4.13	4.13	2.67
$\omega_8(a')$	1083.0	−22.96	13.69	−8.42	−7.59	−10.37	−12.22	−14.00	24.61	3.43	3.43	2.06
$\omega_9(a')$	381.9	−1.39	−3.52	20.20	−0.76	1.83	5.18	−7.56	−9.35	15.49	15.49	−3.56
$\omega_{10}(a')$	141.1	−0.37	−2.56	7.32	−2.94	−4.70	−3.29	4.43	1.14	11.29	11.29	−1.17
$\omega_{11}(a')$	60.4	147.51	−2.31	22.40	−108.58	5.50	3.19	−147.73	−6.33	20.58	20.58	2.37
$\omega_{12}(a'')$	3591.3	141.26	109.66	101.46	23.66	25.24	24.16	121.04	88.50	78.38	78.38	0.23
$\omega_{13}(a'')$	3587.7	139.81	111.46	102.72	23.92	27.09	26.06	118.00	89.07	80.15	80.15	0.70
$\omega_{14}(a'')$	1696.7	5.00	9.07	−11.25	2.99	−4.39	−6.30	6.73	15.34	−5.16	−5.16	1.96
$\omega_{15}(a'')$	1674.1	3.73	7.14	−11.12	−1.34	−5.58	−6.76	6.16	14.11	−4.29	−4.29	1.51
$\omega_{16}(a'')$	238.1	27.28	1.18	2.56	21.08	1.74	2.75	23.86	−2.36	−0.09	−0.09	−0.79
$\omega_{17}(a'')$	105.0	6.40	0.39	−4.79	6.44	−1.87	−0.05	4.91	−1.29	−5.74	−5.74	−3.30
$\omega_{18}(a'')$	42.8	7.84	−7.30	5.86	6.09	−4.20	−2.51	7.65	−8.27	6.80	6.80	−3.29

Natural Internal Coordinates

Table S9: Symmetrized, unnormalized natural internal coordinates for ammonia.

1	$r_{1,4} + r_{1,2} + r_{1,3}$
2	$2r_{1,4} - r_{1,2} - r_{1,3}$
3	$r_{1,2} - r_{1,3}$
4	$r_{5,7} + r_{5,6} + r_{5,8}$
5	$2r_{5,7} - r_{5,6} - r_{5,8}$
6	$r_{5,6} - r_{5,8}$
7	$r_{1,5}$
8	$2\phi_{2,1,3} - \phi_{4,1,2} - \phi_{4,1,3}$
9	$\phi_{4,1,2} - \phi_{4,1,3}$
10	$2\phi_{6,5,8} - \phi_{7,5,6} - \phi_{7,5,8}$
11	$\phi_{7,5,6} - \phi_{7,5,8}$
12	$\phi_{4,5,7}$
13	$\phi_{5,4,1}$
14	$\tau_{7,5,4,1}$
15	$\tau(6,8\|7\|5\|4)$
16	$\tau(2,3\|4\|1\|5)$
17	$\gamma_{4,1,2,3} + \gamma_{2,1,3,4} + \gamma_{3,1,4,2}$
18	$\gamma_{7,5,6,8} + \gamma_{6,5,8,7} + \gamma_{8,5,7,6}$

S3.3 water − ammonia dimer

Structure

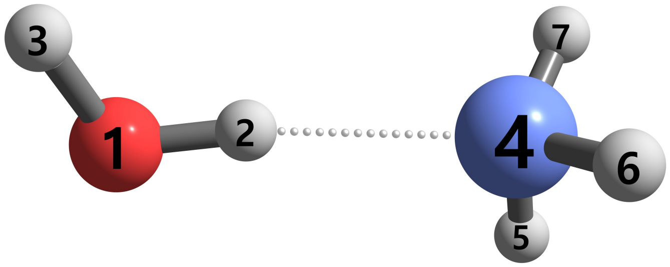

Figure S3: Water-ammonia dimer structure labeled with atomic indices.

Literature Comparison

Table S10: A comparison of key geometric features to the highest available level of theory Literature geometric structure. Units are either Å or °, depending on the coordinate type.

Geometric Parameter	This work CCSD(T)/aug-cc-pVTZ	Grein CCSD(T)/CBS[4]	Difference
$r_{N_4H_7}$	1.0149	1.0127	−0.0022
$r_{N_4H_5}$	1.0154	1.0132	−0.0022
$r_{O_1H_2}$	0.9733	0.9702	−0.0031
$r_{O_1H_3}$	0.9607	0.9576	−0.0031
$\theta_{H_5N_4H_6}$	106.58	106.70	0.12
$\theta_{H_7N_4H_5}$	106.58	106.70	0.12
$\theta_{H_3O_1H_2}$	104.75	104.96	0.21
$r_{N_4 \cdots H_2}$	1.9741	1.9850	0.0109
$\theta_{H_7N_4 \cdots H_2}$	116.36	116.50	0.14
$\theta_{O_1H_2 \cdots N_4}$	170.64	169.98	−0.66

Frequencies

Table S11: CMA-0A residuals with respect to the reference harmonic frequencies (in cm^{-1}) for $H_2O \cdots NH_3$ using natural internal coordinates with cartesian force constants.

	Reference	CMA-0A										
	CCSD(T)	MP2						CCSD(T)				
	aTZ	DZ	haDZ	aDZ	TZ	haTZ	aTZ	DZ	haDZ	aDZ	TZ	haTZ
$\omega_1(a')$	3883.3	−1.0	−0.1	−0.2	−0.1	−0.1	0.0	−1.1	−0.1	−0.1	−0.1	0.0
$\omega_2(a')$	3620.4	1.0	0.1	0.1	0.2	−0.6	−12.2	1.2	−0.2	−0.5	0.1	0.1
$\omega_3(a')$	3590.5	0.0	0.0	0.0	−0.1	0.6	3.5	0.0	0.3	0.7	0.0	−0.1
$\omega_4(a')$	3465.0	0.0	0.0	0.0	0.0	0.0	0.0	0.0	0.0	0.0	0.0	0.0
$\omega_5(a')$	1689.4	−1.0	−0.3	0.1	0.1	−0.6	−0.9	−2.1	0.0	−0.1	−0.5	0.1
$\omega_6(a')$	1663.2	0.8	0.2	−0.2	−0.1	0.6	0.8	1.9	−0.2	0.0	0.4	−0.2
$\omega_7(a')$	1112.6	0.2	0.1	0.1	0.0	0.0	0.0	0.2	0.1	0.1	0.0	0.0
$\omega_8(a')$	449.5	−0.1	−0.1	0.1	−0.1	0.0	0.0	−0.1	−0.2	0.0	−0.1	0.0
$\omega_9(a')$	198.3	−1.2	−1.1	0.1	−0.2	0.0	−0.2	−1.3	−0.2	−0.1	−0.6	0.0
$\omega_{10}(a')$	166.3	2.3	2.2	0.1	0.7	0.1	0.3	2.5	1.1	0.3	1.0	0.1
$\omega_{11}(a'')$	3594.2	−0.1	0.0	0.0	0.0	0.0	8.8	0.0	−0.1	−0.2	0.0	0.0
$\omega_{12}(a'')$	1672.9	0.4	0.2	0.2	0.1	0.1	0.1	0.4	0.3	0.3	0.2	0.1
$\omega_{13}(a'')$	698.2	−0.1	0.0	0.1	−0.2	0.1	0.1	0.0	0.0	0.0	−0.1	0.0
$\omega_{14}(a'')$	175.0	−0.1	0.3	0.0	0.8	0.0	0.1	−0.2	0.2	0.0	0.6	0.1
$\omega_{15}(a'')$	19.2	4.6	0.1	0.6	0.3	0.3	0.1	4.1	0.1	0.7	0.3	0.3

Table S12: Pure level B residuals with respect to the reference harmonic frequencies (in cm^{-1}) for $H_2O \cdots NH_3$ using natural internal coordinates with cartesian force constants.

	Reference	Pure										
	CCSD(T)	MP2						CCSD(T)				
	aTZ	DZ	haDZ	aDZ	TZ	haTZ	aTZ	DZ	haDZ	aDZ	TZ	haTZ
$\omega_1(a')$	3883.3	91.70	76.56	68.31	19.14	24.50	21.09	71.33	56.55	48.12	−2.70	3.65
$\omega_2(a')$	3620.4	114.57	77.79	67.28	9.11	−2.39	−5.92	101.07	58.06	46.45	12.85	4.06
$\omega_3(a')$	3590.5	127.32	72.35	63.66	24.15	24.47	22.91	125.33	78.81	70.48	0.07	0.88
$\omega_4(a')$	3465.0	112.36	82.31	70.04	8.15	8.29	8.49	104.60	76.35	62.50	−1.25	−0.30
$\omega_5(a')$	1689.4	12.29	2.19	−13.34	−5.84	−11.45	−13.12	25.53	15.53	0.27	8.42	2.11
$\omega_6(a')$	1663.2	2.30	1.66	−15.77	−5.14	−9.42	−11.26	6.34	12.22	−6.01	2.78	1.46
$\omega_7(a')$	1112.6	−26.02	17.39	−6.62	−10.25	−11.47	−11.97	−17.42	28.47	5.33	0.66	0.59
$\omega_8(a')$	449.5	12.61	8.26	0.50	0.79	−1.26	1.76	10.35	6.82	−2.03	−1.36	−3.48
$\omega_9(a')$	198.3	9.82	3.91	−0.92	−1.29	−2.89	−3.83	13.37	7.69	4.15	2.50	0.94
$\omega_{10}(a')$	166.3	−11.33	7.57	−2.29	−11.04	−3.50	−1.47	−10.19	10.37	−1.53	−10.22	−2.70
$\omega_{11}(a'')$	3594.2	142.91	107.77	97.76	23.72	24.17	23.02	123.65	87.73	76.53	−0.59	0.24
$\omega_{12}(a'')$	1672.9	1.57	4.81	−13.78	−2.89	−6.08	−6.88	4.07	11.79	−6.96	1.65	0.82
$\omega_{13}(a'')$	698.2	34.42	21.58	15.61	9.41	1.75	3.39	30.86	18.94	12.49	7.31	−1.74
$\omega_{14}(a'')$	175.0	−0.36	16.28	5.24	−7.40	−1.65	−0.12	−0.04	16.63	4.75	−5.82	−1.40
$\omega_{15}(a'')$	19.2	−51.51	3.22	−31.96	12.71	2.83	6.72	−49.97	3.31	−34.32	12.62	0.91

Natural Internal Coordinates

Table S13: Symmetrized, unnormalized natural internal coordinates for water$\cdots$ammonia.

1	$r_{1,2} + r_{1,3}$
2	$r_{1,2} - r_{1,3}$
3	$r_{4,5} + r_{4,6} + r_{4,7}$
4	$2r_{4,5} - r_{4,6} - r_{4,7}$
5	$r_{4,6} - r_{4,7}$
6	$r_{4,2}$
7	$\phi_{3,1,2}$
8	$2\phi_{6,4,7} - \phi_{5,4,6} - \phi_{5,4,7}$
9	$\phi_{5,4,6} - \phi_{5,4,7}$
10	$\phi(4\|2\|2,3)$
11	$\phi_{5,4,2}$
12	$\tau(2,3\|2\|4\|5)$
13	$\tau(1\|2,3\|2\|4)$
14	$\tau(6,7\|5\|4\|2)$
15	$\gamma_{5,4,6,7} + \gamma_{6,4,7,5} + \gamma_{7,4,5,6}$

S3.4 formic acid dimer

Structure

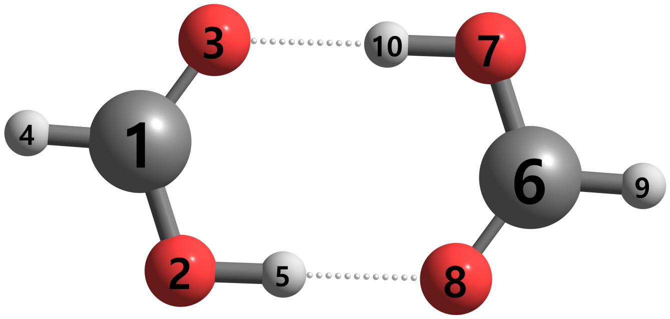

Figure S4: Formic Acid dimer structure labeled with atomic indices.

Literature Comparison

Table S14: A comparison of key geometric features to the highest available level of theory Literature geometric structure. Units are either Å or °, depending on the coordinate type.

Geometric Parameter	This work CCSD(T)/aug-cc-pVTZ	Xantheas CCSD(T)/aug-cc-pVQZ[5]	Difference
$r_{O_3C_1}$	1.2220	1.2186	−0.0034
$r_{H_4C_1}$	1.0939	1.0932	−0.0007
$r_{O_2C_1}$	1.3160	1.3127	−0.0033
$r_{O_2H_5}$	0.9957	0.9934	−0.0023
$\theta_{O_2C_1H_4}$	111.77	111.81	0.04
$\theta_{O_3C_1H_4}$	122.02	122.00	−0.02
$\theta_{C_1O_2H_5}$	109.55	109.72	0.17
$r_{H_5 \cdots O_8}$	1.6784	1.6822	0.0038
$\theta_{O_2H_5 \cdots O_8}$	179.43	178.75	−0.68

Frequencies

Table S15: CMA-0A residuals with respect to the reference harmonic frequencies (in cm^{-1}) for $(HCOOH)_2$ using natural internal coordinates with cartesian force constants.

	Reference	CMA-0A										
	CCSD(T)	MP2						CCSD(T)				
	aTZ	DZ	haDZ	aDZ	TZ	haTZ	aTZ	DZ	haDZ	aDZ	TZ	haTZ
$\omega_1(a_g)$	3190.6	−7.2	−20.4	−38.6	−0.1	−0.1	−0.1	−0.9	−16.4	−5.8	0.0	0.0
$\omega_2(a_g)$	3098.5	7.4	20.8	39.2	0.1	0.1	0.1	0.8	16.7	5.9	0.0	0.0
$\omega_3(a_g)$	1708.0	−2.4	−0.8	−0.9	−0.5	−0.3	−0.3	−1.7	−0.3	−0.3	−0.1	0.0
$\omega_4(a_g)$	1486.8	1.6	0.3	0.4	−0.5	−0.6	−0.4	1.4	0.2	0.3	0.0	0.0
$\omega_5(a_g)$	1404.2	0.4	0.4	0.3	0.4	0.6	0.5	0.1	−0.1	−0.1	0.0	0.0
$\omega_6(a_g)$	1250.2	0.9	0.4	0.4	0.8	0.5	0.3	0.7	0.2	0.2	0.1	0.0
$\omega_7(a_g)$	682.2	0.1	0.1	0.1	0.0	0.0	0.0	0.1	0.0	0.0	0.0	0.0
$\omega_8(a_g)$	211.1	−0.1	0.1	0.0	−0.3	0.0	0.0	0.1	0.0	−0.1	−0.1	0.0
$\omega_9(a_g)$	168.2	0.2	0.0	0.1	0.5	0.1	0.1	0.1	0.2	0.3	0.2	0.0
$\omega_{10}(b_g)$	1080.2	−0.8	−0.4	−0.3	−0.2	−0.1	−0.1	−0.6	−0.1	−0.1	0.0	0.0
$\omega_{11}(b_g)$	963.2	1.0	0.5	0.4	0.3	0.2	0.2	0.6	0.3	0.2	0.1	0.1
$\omega_{12}(b_g)$	252.6	0.3	0.0	0.0	0.1	0.0	0.0	0.3	0.0	0.0	0.1	0.0
$\omega_{13}(a_u)$	1101.7	−0.9	−0.3	−0.2	−0.1	0.0	−0.1	−0.6	−0.1	0.0	0.0	0.0
$\omega_{14}(a_u)$	987.1	1.1	0.4	0.3	0.2	0.1	0.2	0.8	0.2	0.1	0.1	0.1
$\omega_{15}(a_u)$	175.9	0.2	0.1	0.1	0.0	0.1	0.0	0.1	0.0	0.1	0.0	0.0
$\omega_{16}(a_u)$	69.5	0.1	0.1	0.1	0.1	0.0	0.1	0.2	0.1	0.2	0.2	0.0
$\omega_{17}(b_u)$	3294.4	−1.5	−2.8	−2.3	−0.1	−0.1	−0.1	−0.7	−1.4	−1.1	0.0	0.0
$\omega_{18}(b_u)$	3094.7	1.5	3.0	2.4	0.1	0.1	0.1	0.7	1.5	1.1	0.0	0.0
$\omega_{19}(b_u)$	1772.6	−0.9	−0.4	−0.5	−0.3	−0.2	−0.2	−0.6	−0.1	−0.1	−0.1	0.0
$\omega_{20}(b_u)$	1458.2	−2.4	−0.7	−0.3	−1.4	−1.7	−0.9	0.0	0.0	0.1	0.0	−0.1
$\omega_{21}(b_u)$	1401.5	3.0	1.0	0.7	1.1	1.6	0.9	0.5	0.0	0.0	0.1	0.1
$\omega_{22}(b_u)$	1254.8	0.9	0.4	0.3	0.7	0.6	0.4	0.3	0.1	0.1	0.1	0.0
$\omega_{23}(b_u)$	710.8	0.1	0.0	0.0	0.0	0.0	0.0	0.1	0.0	0.0	0.0	0.0
$\omega_{24}(b_u)$	277.1	0.1	0.1	0.1	0.1	0.1	0.1	0.2	0.2	0.2	0.1	0.1

Table S16: Pure level B residuals with respect to the reference harmonic frequencies (in cm^{-1}) for $(HCOOH)_2$ using natural internal coordinates with cartesian force constants.

	Reference	Pure										
	CCSD(T)	MP2						CCSD(T)				
	aTZ	DZ	haDZ	aDZ	TZ	haTZ	aTZ	DZ	haDZ	aDZ	TZ	haTZ
$\omega_1(a_g)$	3190.6	78.71	42.95	36.01	−4.29	−5.66	−7.22	84.97	39.71	37.02	0.88	1.44
$\omega_2(a_g)$	3098.5	152.90	118.74	115.27	15.26	17.92	15.98	143.80	117.87	109.66	−1.57	1.81
$\omega_3(a_g)$	1708.0	75.08	46.47	44.00	12.12	2.20	1.90	68.50	43.42	40.94	8.66	0.22
$\omega_4(a_g)$	1486.8	18.25	9.52	7.02	−9.96	−13.40	−11.89	29.67	21.43	18.94	1.23	−1.87
$\omega_5(a_g)$	1404.2	32.39	13.45	11.72	6.66	1.56	−1.29	31.95	15.05	13.02	7.77	3.11
$\omega_6(a_g)$	1250.2	37.64	22.85	22.09	2.21	−0.05	1.06	34.17	20.86	20.03	0.84	−1.06
$\omega_7(a_g)$	682.2	11.95	4.05	3.97	2.05	−0.30	−0.05	10.56	3.42	3.35	1.63	−0.32
$\omega_8(a_g)$	211.1	1.31	7.18	6.10	−5.30	−2.77	−3.15	4.49	11.35	10.52	−2.47	0.47
$\omega_9(a_g)$	168.2	1.36	3.75	2.85	−4.11	−2.55	−2.83	3.79	6.57	5.52	−1.43	0.26
$\omega_{10}(b_g)$	1080.2	25.29	18.77	16.72	10.00	5.41	4.63	18.69	12.59	10.04	5.73	1.24
$\omega_{11}(b_g)$	963.2	30.67	21.10	18.58	14.04	4.96	6.15	26.46	16.12	13.91	9.82	−0.94
$\omega_{12}(b_g)$	252.6	33.26	8.76	9.24	14.53	3.12	2.64	30.29	5.32	5.54	12.34	0.53
$\omega_{13}(a_u)$	1101.7	32.39	22.62	21.01	12.64	7.25	7.62	23.34	13.92	11.45	5.82	0.17
$\omega_{14}(a_u)$	987.1	20.76	13.29	7.94	4.19	−0.90	1.27	19.39	11.68	6.12	3.86	−2.16
$\omega_{15}(a_u)$	175.9	12.29	3.21	1.48	6.46	0.55	1.14	11.03	1.76	−0.02	5.83	−0.24
$\omega_{16}(a_u)$	69.5	5.93	0.45	0.40	2.29	1.20	0.73	4.90	−0.54	−0.82	2.01	0.87
$\omega_{17}(b_u)$	3294.4	90.08	32.62	30.07	3.39	−3.96	−5.36	95.18	36.99	34.70	6.88	1.27
$\omega_{18}(b_u)$	3094.7	150.72	128.70	120.34	14.54	17.63	15.76	141.27	118.51	109.34	−2.05	1.71
$\omega_{19}(b_u)$	1772.6	78.01	43.83	41.68	11.68	0.89	0.28	73.43	42.42	40.26	9.86	0.61
$\omega_{20}(b_u)$	1458.2	15.19	10.09	8.54	−5.09	−9.01	−8.24	21.45	18.00	16.92	2.79	−1.54
$\omega_{21}(b_u)$	1401.5	29.61	11.11	10.18	5.55	−0.88	−2.17	31.55	13.89	12.46	7.82	1.91
$\omega_{22}(b_u)$	1254.8	34.69	23.04	22.32	2.21	0.39	1.39	31.32	20.84	20.06	0.57	−0.91
$\omega_{23}(b_u)$	710.8	12.69	5.50	5.32	3.22	−0.49	−0.16	11.13	4.80	4.73	3.15	−0.41
$\omega_{24}(b_u)$	277.1	6.40	5.35	4.43	−3.91	−3.36	−3.60	10.57	10.23	9.48	0.01	0.69

Natural Internal Coordinates

Table S17: Symmetrized, unnormalized natural internal coordinates for formic acid dimer.

1	$r_{2,5} + r_{7,10}$
2	$r_{2,5} - r_{7,10}$
3	$r_{1,2} + r_{6,7}$
4	$r_{1,2} - r_{6,7}$
5	$r_{1,3} + r_{6,8}$
6	$r_{1,3} - r_{6,8}$
7	$r_{1,4} + r_{6,9}$
8	$r_{1,4} - r_{6,9}$
9	$r(3, 5\vert 8, 10)$
10	$\phi_{2,1,3} + \phi_{7,6,8}$
11	$\phi_{2,1,3} - \phi_{7,6,8}$
12	$\phi_{1,2,5} + \phi_{6,7,10}$
13	$\phi_{1,2,5} - \phi_{6,7,10}$
14	$\phi_{3,1,4} - \phi_{2,1,4} + \phi_{8,6,9} - \phi_{7,6,9}$
15	$\phi_{3,1,4} - \phi_{2,1,4} - \phi_{8,6,9} + \phi_{7,6,9}$
16	$\phi(2, 1\vert 3, 5\vert 8, 10) + \phi(6, 7\vert 8, 10\vert 3, 5)$
17	$\phi(2, 1\vert 3, 5\vert 8, 10) - \phi(6, 7\vert 8, 10\vert 3, 5)$
18	$\tau_{3,1,2,5} + \tau_{8,6,7,10}$
19	$\tau_{3,1,2,5} - \tau_{8,6,7,10}$
20	$\tau(2, 1\vert 3, 5\vert 8, 10\vert 6, 7)$
21	$\tau(4\vert 2, 1\vert 3, 5\vert 8, 10) + \tau(9\vert 6, 7\vert 8, 10\vert 3, 5)$
22	$\tau(4\vert 2, 1\vert 3, 5\vert 8, 10) - \tau(9\vert 6, 7\vert 8, 10\vert 3, 5)$
23	$\gamma_{4,1,2,3} + \gamma_{9,6,7,8}$
24	$\gamma_{4,1,2,3} - \gamma_{9,6,7,8}$

S3.5 nitrosomethane–ammonia dimer

Structure

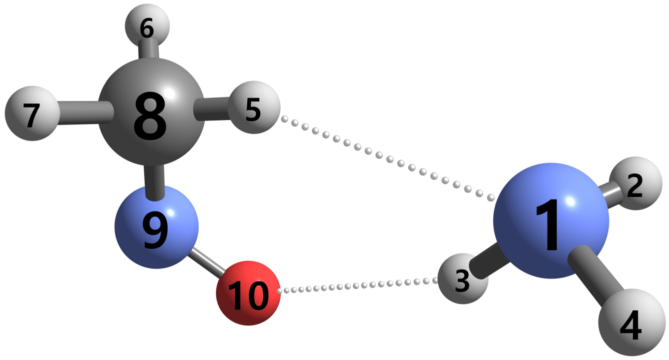

Figure S5: Nitrosomethane-Ammonia dimer structure labeled with atomic indices.

Frequencies

Table S18: CMA-0A residuals with respect to the reference harmonic frequencies (in cm^{-1}) for $CH_3NO\cdots NH_3$ using natural internal coordinates with cartesian force constants.

	Reference	CMA-0A										
	CCSD(T)	MP2						CCSD(T)				
	aTZ	DZ	haDZ	aDZ	TZ	haTZ	aTZ	DZ	haDZ	aDZ	TZ	haTZ
$\omega_1(a')$	3580.20	−0.15	−0.02	0.00	0.01	−0.01	−0.01	−0.27	−0.00	−0.01	0.00	0.02
$\omega_2(a')$	3453.90	0.05	0.00	−0.02	−0.01	0.02	0.02	0.21	−0.01	0.01	0.00	0.00
$\omega_3(a')$	3147.71	−0.09	−0.07	−0.03	−0.08	−0.06	−0.07	−0.10	−0.01	−0.01	−0.01	−0.01
$\omega_4(a')$	3027.42	−0.03	0.06	0.01	0.07	0.06	0.07	−0.01	−0.01	−0.01	−0.00	0.00
$\omega_5(a')$	1671.49	0.02	0.01	−0.03	−0.01	−0.00	−0.00	−0.10	−0.02	−0.14	−0.01	0.00
$\omega_6(a')$	1579.76	−0.65	−0.49	−0.63	−0.08	−0.38	−0.39	−0.78	−0.77	−0.85	−0.07	−0.00
$\omega_7(a')$	1472.43	0.66	0.36	0.50	−0.08	0.11	0.10	0.89	0.48	0.75	0.06	−0.02
$\omega_8(a')$	1396.40	−0.06	−0.00	−0.00	0.10	0.26	0.29	0.11	0.14	0.18	0.03	0.02
$\omega_9(a')$	1166.51	−0.03	−0.03	−0.00	0.08	0.03	0.03	−0.06	0.10	0.01	0.01	−0.00
$\omega_{10}(a')$	1086.79	0.14	0.08	0.09	−0.00	0.01	0.01	0.06	0.04	0.04	0.03	0.00
$\omega_{11}(a')$	875.13	0.62	0.37	0.41	0.04	0.03	0.03	0.46	0.34	0.33	0.01	0.00
$\omega_{12}(a')$	580.33	0.12	0.10	0.11	0.07	0.05	0.05	0.03	0.06	0.05	0.01	0.00
$\omega_{13}(a')$	279.83	0.26	0.04	−0.12	0.13	−0.03	−0.00	0.21	0.07	−0.14	0.10	−0.01
$\omega_{14}(a')$	106.08	−1.93	−0.34	0.06	−0.38	0.03	0.00	−1.90	−0.24	0.05	−0.54	0.05
$\omega_{15}(a')$	96.47	2.93	0.51	0.48	0.51	0.15	0.06	2.94	0.35	0.52	0.65	0.03
$\omega_{16}(a'')$	3587.29	−0.02	−0.01	−0.01	−0.00	−0.00	−0.00	−0.01	−0.00	−0.01	−0.00	0.00
$\omega_{17}(a'')$	3116.66	−0.08	−0.02	−0.02	−0.01	−0.00	−0.00	−0.06	−0.01	−0.01	−0.00	0.00
$\omega_{18}(a'')$	1684.09	−0.04	0.02	0.02	−0.02	0.00	0.00	−0.07	0.01	0.01	−0.01	0.00
$\omega_{19}(a'')$	1482.64	−0.24	0.02	0.01	−0.03	−0.00	−0.00	−0.24	0.00	0.01	−0.02	0.00
$\omega_{20}(a'')$	989.63	0.22	0.05	0.02	−0.02	0.01	0.01	0.29	0.04	0.02	−0.00	−0.00
$\omega_{21}(a'')$	178.21	13.43	−0.11	−0.34	−1.48	−0.00	0.00	−14.00	0.02	−0.31	−1.65	−0.00
$\omega_{22}(a'')$	128.83	7.93	−0.07	−1.56	−3.18	0.04	−0.04	7.12	−0.07	−2.37	−4.00	−0.15
$\omega_{23}(a'')$	93.98	−0.36	−0.11	−9.71	4.69	0.01	0.11	−1.56	−0.08	−9.97	5.18	0.20
$\omega_{24}(a'')$	57.08	26.17	0.81	17.56	5.81	0.06	0.05	28.68	0.57	19.01	6.42	0.04

Table S19: Pure level B residuals with respect to the reference harmonic frequencies (in cm^{-1}) for $CH_3NO\cdots NH_3$ using natural internal coordinates with cartesian force constants.

	Reference	Pure										
	CCSD(T)	MP2						CCSD(T)				
	aTZ	DZ	haDZ	aDZ	TZ	haTZ	aTZ	DZ	haDZ	aDZ	TZ	haTZ
$\omega_1(a')$	3580.20	144.28	105.59	99.23	22.18	21.96	21.00	127.66	87.97	80.69	0.54	0.83
$\omega_2(a')$	3453.90	110.91	81.82	72.83	6.93	7.41	8.03	103.01	76.06	65.77	−2.20	−0.67
$\omega_3(a')$	3147.71	162.08	135.04	125.18	16.11	16.99	17.22	150.16	122.09	111.10	−2.21	−0.63
$\omega_4(a')$	3027.42	134.25	115.54	105.10	8.79	9.28	10.04	129.03	109.73	97.60	−2.11	−1.02
$\omega_5(a')$	1671.49	−2.81	5.41	−8.96	−7.03	−6.06	−7.35	0.48	12.57	−1.97	−1.96	1.05
$\omega_6(a')$	1579.76	42.35	37.74	36.43	−9.44	−17.02	−17.09	56.91	53.42	52.15	6.83	0.04
$\omega_7(a')$	1472.43	10.39	14.00	1.27	−1.19	2.34	2.97	8.59	11.40	−1.39	−4.85	−0.42
$\omega_8(a')$	1396.40	4.63	3.61	−7.60	−12.74	−9.25	−7.44	9.91	11.13	−0.60	−6.38	−1.91
$\omega_9(a')$	1166.51	15.57	12.02	9.42	−1.92	−2.08	−2.68	15.43	12.76	10.21	0.06	0.46
$\omega_{10}(a')$	1086.79	−19.16	11.25	−11.63	−9.42	−11.29	−13.32	−10.11	22.84	1.12	2.23	1.80
$\omega_{11}(a')$	875.13	25.93	23.28	22.10	−1.40	−2.63	−3.14	26.95	26.13	24.92	1.30	0.58
$\omega_{12}(a')$	580.33	9.56	5.45	4.53	−1.52	−0.40	−0.11	8.18	4.78	4.04	−1.87	−0.56
$\omega_{13}(a')$	279.83	14.22	−5.92	−8.73	3.06	−1.58	−2.34	15.06	−5.84	−9.87	4.18	−1.20
$\omega_{14}(a')$	106.08	6.35	0.88	3.93	−3.53	−2.51	−1.28	7.44	1.85	5.40	−2.64	−1.49
$\omega_{15}(a')$	96.47	13.16	3.10	−0.71	−0.03	−1.19	−0.50	14.59	4.25	0.10	0.62	−0.30
$\omega_{16}(a'')$	3587.29	141.35	110.78	102.05	24.92	26.47	25.28	120.49	89.20	79.32	−0.92	1.07
$\omega_{17}(a'')$	3116.66	163.41	139.26	127.97	20.84	21.97	22.30	146.22	121.60	109.02	−2.67	−0.79
$\omega_{18}(a'')$	1684.09	8.17	6.43	−11.74	−0.23	−6.23	−7.23	10.63	13.64	−5.08	4.52	1.03
$\omega_{19}(a'')$	1482.64	22.00	13.17	−0.38	2.86	1.93	2.82	20.70	11.42	−2.02	−0.45	−0.58
$\omega_{20}(a'')$	989.63	3.09	1.29	−1.69	−6.51	−7.50	−6.69	7.52	7.08	3.97	−0.62	−0.90
$\omega_{21}(a'')$	178.21	36.35	−3.39	1.41	8.74	−0.31	0.41	34.34	−5.04	0.49	7.62	−0.68
$\omega_{22}(a'')$	128.83	55.62	−1.88	12.30	25.73	−2.15	−2.19	54.99	−1.13	12.75	25.96	0.02
$\omega_{23}(a'')$	93.98	28.33	−3.61	−12.27	12.41	−3.50	−6.87	28.08	−2.24	−10.07	13.52	1.61
$\omega_{24}(a'')$	57.08	36.73	−3.67	−7.31	23.58	−5.37	−6.36	35.55	1.79	−8.21	24.92	1.18

Natural Internal Coordinates

Table S20: Symmetrized, unnormalized natural internal coordinates for ammonia−nitrosmethane.

1	$r_{1,3} + r_{1,2} + r_{1,4}$
2	$2r_{1,3} - r_{1,2} - r_{1,4}$
3	$r_{1,2} - r_{1,4}$
4	$r_{10,9}$
5	$r_{9,8}$
6	$r_{8,5} + r_{8,6} + r_{8,7}$
7	$2r_{8,5} - r_{8,6} - r_{8,7}$
8	$r_{8,6} - r_{8,7}$
9	$r(1,3\|5,10)$
10	$2\phi_{2,1,4} - \phi_{3,1,2} - \phi_{3,1,4}$
11	$\phi_{3,1,2} - \phi_{3,1,4}$
12	$\phi_{8,9,10}$
13	$\phi_{6,8,7} + \phi_{5,8,6} + \phi_{5,8,7} - \phi_{5,8,9} - \phi_{7,8,9} - \phi_{6,8,9}$
14	$2\phi_{6,8,7} - \phi_{5,8,6} - \phi_{5,8,7}$
15	$\phi_{5,8,6} - \phi_{5,8,7}$
16	$2\phi_{5,8,9} - \phi_{7,8,9} - \phi_{6,8,9}$
17	$\phi_{7,8,9} - \phi_{6,8,9}$
18	$\phi(5,10\|1,3\|2,4)$
19	$\phi(1,3\|5,10\|8,9)$
20	$\tau_{5,8,9,10} + \tau_{6,8,9,10} + \tau_{7,8,9,10}$
21	$\tau(2,4\|1,3\|5,10\|8,9)$
22	$\tau(2\|2,4\|1,3\|5,10) + \tau(4\|2,4\|1,3\|5,10)$
23	$\tau(1,3\|5,10\|8,9\|6,7)$
24	$\gamma_{3,1,2,4} + \gamma_{2,1,4,3} + \gamma_{4,1,3,2}$

S3.6 formamide dimer

Structure

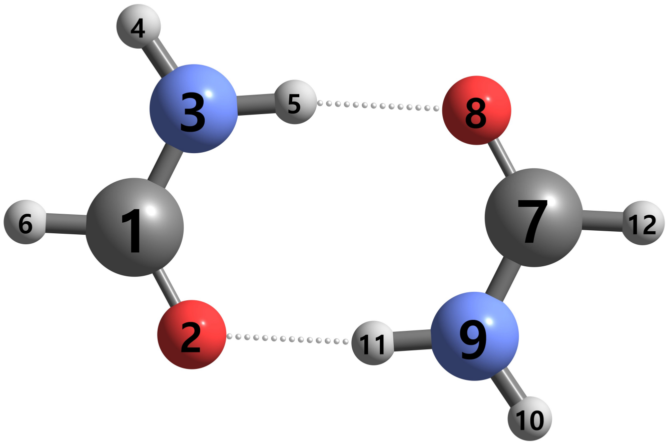

Figure S6: Formamide dimer structure labeled with atomic indices.

Literature Comparison

Table S21: A comparison of key geometric features to the highest available level of theory Literature geometric structure. Units are either Å or °, depending on the coordinate type.

Geometric Parameter	This work CCSD(T)/cc-pVTZ	This work CCSD(T)/h-aug-cc-pVTZ	Puzzarini CCSD(T)/CBS[6]
$r_{H_4N_3}$	1.0032	1.0047	1.0023
$r_{H_5N_3}$	1.0226	1.0024	1.0216
$r_{C_1N_3}$	1.3424	1.3431	1.3383
$r_{C_1H_6}$	1.1004	1.1003	1.0987
$r_{C_1O_2}$	1.2290	1.2312	1.2256
$\theta_{H_4N_3C_1}$	119.21	119.33	119.43
$\theta_{H_5N_3C_1}$	120.73	120.52	120.41
$\theta_{N_3C_1O_2}$	125.49	125.16	125.06
$\theta_{N_3C_1H_6}$	113.60	113.89	121.58
$r_{H_5 \cdots O_8}$	1.8407	1.8472	1.8375
$\theta_{N_3H_5 \cdots O_8}$	174.34	173.23	172.97

Table S22: A comparison of key geometric features to the highest available level of theory Literature geometric structure. Units are either Å or °, depending on the coordinate type.

Geometric Parameter	This work CCSD(T)/aug-cc-pVTZ	This work CCSD(T)/aug-cc-pVQZ	Puzzarini CCSD(T)/CBS[6]
$r_{H_4N_3}$	1.0047	1.0032	1.0023
$r_{H_5N_3}$	1.0238	1.0225	1.0216
$r_{C_1N_3}$	1.3433	1.3404	1.3383
$r_{C_1H_6}$	1.1002	1.0995	1.0987
$r_{C_1O_2}$	1.2312	1.2276	1.2256
$\theta_{H_4N_3C_1}$	119.35	119.38	119.43
$\theta_{H_5N_3C_1}$	120.52	120.47	120.41
$\theta_{N_3C_1O_2}$	125.11	125.08	125.06
$\theta_{N_3C_1H_6}$	113.91	113.93	121.58
$r_{H_5 \cdots O_8}$	1.8441	1.8458	1.8375
$\theta_{N_3H_5 \cdots O_8}$	173.55	173.24	172.97

Frequencies

Table S23: CMA-0A residuals with respect to the reference harmonic frequencies (in cm^{-1}) for $(HCONH_2)_2$ using natural internal coordinates with cartesian force constants.

	Reference	CMA-0A										
	CCSD(T)	MP2						CCSD(T)				
	aTZ	DZ	haDZ	aDZ	TZ	haTZ	aTZ	DZ	haDZ	aDZ	TZ	haTZ
$\omega_1(a_g)$	3688.2	−0.2	−0.1	−0.1	0.0	−0.1	0.0	−0.3	−0.1	−0.1	0.0	0.0
$\omega_2(a_g)$	3308.2	0.2	0.0	0.0	0.0	0.0	0.0	0.2	0.0	0.0	0.0	0.0
$\omega_3(a_g)$	3010.6	0.0	0.0	0.0	0.0	0.0	0.0	0.0	0.0	0.0	0.0	0.0
$\omega_4(a_g)$	1740.2	−6.4	−2.2	−2.6	−1.3	−0.6	−0.5	−4.6	−0.9	−1.1	−0.2	0.0
$\omega_5(a_g)$	1640.6	4.5	1.3	1.6	1.0	0.5	0.3	3.3	0.5	0.7	0.2	0.0
$\omega_6(a_g)$	1417.5	0.5	0.9	0.4	0.0	−0.1	0.0	0.0	−0.1	−0.1	0.0	0.0
$\omega_7(a_g)$	1348.0	1.2	0.4	0.5	0.3	0.3	0.2	1.0	0.4	0.7	0.1	0.0
$\omega_8(a_g)$	1101.7	1.4	0.8	0.6	0.1	0.1	0.1	1.0	0.4	0.3	0.0	0.0
$\omega_9(a_g)$	606.3	0.1	0.0	0.1	0.0	0.0	0.0	0.1	0.0	0.1	0.0	0.0
$\omega_{10}(a_g)$	181.1	0.0	0.1	−0.1	−0.2	0.0	0.0	0.1	0.1	0.1	−0.1	0.0
$\omega_{11}(a_g)$	138.5	0.2	0.0	0.0	0.3	0.1	0.0	0.3	0.0	0.0	0.1	0.0
$\omega_{12}(b_g)$	1061.6	−0.1	0.0	−0.1	0.0	0.0	0.0	−0.1	0.0	−0.1	0.0	0.0
$\omega_{13}(b_g)$	823.2	−0.1	0.0	−0.5	−0.1	0.0	0.0	−0.2	0.0	−0.4	−0.2	0.0
$\omega_{14}(b_g)$	472.7	−2.7	−0.1	0.2	0.1	−0.1	−0.1	−4.2	0.0	0.4	0.0	0.0
$\omega_{15}(b_g)$	162.7	9.0	0.5	2.8	0.4	0.5	0.3	13.3	0.0	1.3	1.1	0.1
$\omega_{16}(a_u)$	1071.2	−0.3	−0.1	−0.4	0.0	0.0	0.0	−0.2	0.0	−0.2	0.0	0.0
$\omega_{17}(a_u)$	856.7	0.3	0.1	0.1	0.0	0.0	0.0	0.2	0.0	0.0	−0.1	0.0
$\omega_{18}(a_u)$	457.0	−0.5	−0.1	0.3	0.2	−0.1	0.0	−1.1	0.0	0.4	0.2	0.0
$\omega_{19}(a_u)$	128.9	0.2	0.6	1.9	0.0	0.4	0.2	0.3	0.1	0.9	0.0	0.1
$\omega_{20}(a_u)$	55.1	5.4	0.0	0.2	0.2	0.1	0.1	9.9	0.1	0.2	0.7	0.0
$\omega_{21}(b_u)$	3688.3	−0.2	0.0	−0.1	0.0	0.1	0.0	−0.3	−0.1	−0.1	0.0	0.0
$\omega_{22}(b_u)$	3358.1	0.2	0.0	0.0	0.0	0.0	0.0	0.3	0.0	0.1	0.0	0.0
$\omega_{23}(b_u)$	3008.5	0.1	0.0	0.0	0.0	0.0	0.0	0.0	0.0	0.0	0.0	0.0
$\omega_{24}(b_u)$	1769.5	−1.9	−0.8	−1.0	−0.3	−0.2	−0.2	−1.3	−0.3	−0.5	−0.1	0.0
$\omega_{25}(b_u)$	1652.0	0.1	−0.1	0.0	0.2	0.1	0.0	0.1	−0.1	−0.1	0.0	0.0
$\omega_{26}(b_u)$	1417.3	0.2	−0.3	0.4	0.0	0.0	0.0	0.0	0.0	−0.1	0.0	0.0
$\omega_{27}(b_u)$	1334.1	1.2	0.3	0.5	0.2	0.1	0.1	1.0	0.2	0.4	0.1	0.0
$\omega_{28}(b_u)$	1094.8	1.3	0.8	0.7	0.1	0.1	0.1	0.9	0.4	0.3	0.0	0.0
$\omega_{29}(b_u)$	627.8	0.2	0.0	0.0	0.0	0.0	0.0	0.2	0.0	0.1	0.0	0.0
$\omega_{30}(b_u)$	227.6	0.1	0.0	0.0	0.0	0.0	0.0	0.1	0.1	0.0	0.0	0.0

Table S24: Pure level B residuals with respect to the reference harmonic frequencies (in cm^{-1}) for $(HCONH_2)_2$ using natural internal coordinates with cartesian force constants.

	Reference	Pure										
	CCSD(T)	MP2						CCSD(T)				
	aTZ	DZ	haDZ	aDZ	TZ	haTZ	aTZ	DZ	haDZ	aDZ	TZ	haTZ
$\omega_1(a_g)$	3688.2	124.62	96.88	86.93	15.58	14.52	13.82	115.07	86.11	74.52	1.73	1.56
$\omega_2(a_g)$	3308.2	114.24	77.47	73.38	−3.15	−2.33	−3.39	119.88	82.82	77.80	−0.63	1.49
$\omega_3(a_g)$	3010.6	149.83	127.80	118.53	14.70	16.71	15.21	140.97	117.90	107.51	−1.11	1.67
$\omega_4(a_g)$	1740.2	80.03	51.26	46.41	16.97	5.01	5.08	68.88	44.76	39.31	9.80	−0.03
$\omega_5(a_g)$	1640.6	19.52	21.91	17.11	−1.88	−4.22	−3.66	22.44	25.97	21.18	0.93	−0.66
$\omega_6(a_g)$	1417.5	34.50	21.48	19.30	7.78	4.52	3.15	30.52	18.59	16.09	4.58	1.39
$\omega_7(a_g)$	1348.0	38.70	29.60	28.64	3.84	−0.53	−0.44	36.94	29.40	27.85	3.70	0.29
$\omega_8(a_g)$	1101.7	16.99	12.80	14.40	−3.69	−3.90	−4.16	18.06	16.09	18.06	−0.99	−0.49
$\omega_9(a_g)$	606.3	14.76	4.41	5.05	3.09	−0.17	−1.05	13.66	4.44	5.20	3.33	0.59
$\omega_{10}(a_g)$	181.1	0.15	3.85	3.35	−6.03	−2.37	−3.57	4.64	8.53	7.75	−2.45	1.52
$\omega_{11}(a_g)$	138.5	6.40	0.29	0.08	0.72	−1.56	−2.05	7.44	2.11	1.92	2.49	0.18
$\omega_{12}(b_g)$	1061.6	18.86	11.85	8.17	1.76	−0.74	1.63	14.80	8.18	3.72	0.51	−1.99
$\omega_{13}(b_g)$	823.2	20.35	10.91	22.30	−6.52	1.02	5.30	14.74	4.95	14.59	−11.96	−3.85
$\omega_{14}(b_g)$	472.7	−9.44	17.83	36.03	−2.77	10.94	9.53	−17.51	7.82	23.44	−11.32	2.51
$\omega_{15}(b_g)$	162.7	4.03	13.42	27.05	8.89	7.37	6.81	−8.06	5.78	18.85	2.39	1.75
$\omega_{16}(a_u)$	1071.2	22.57	13.79	11.55	4.18	0.32	2.66	17.52	9.18	5.74	2.03	−1.88
$\omega_{17}(a_u)$	856.7	14.38	9.92	23.64	−5.35	0.06	4.35	9.22	4.35	17.16	−9.77	−4.08
$\omega_{18}(a_u)$	457.0	−20.86	18.99	39.88	−3.57	13.04	11.14	−31.76	7.42	25.67	−13.55	3.41
$\omega_{19}(a_u)$	128.9	−6.12	8.49	15.24	−0.65	4.17	4.27	−10.58	4.47	10.20	−3.69	1.34
$\omega_{20}(a_u)$	55.1	5.74	1.37	1.52	1.97	−0.70	0.12	4.16	1.77	0.45	2.47	0.67
$\omega_{21}(b_u)$	3688.3	124.60	96.68	86.87	15.32	14.34	13.47	115.21	86.09	74.80	1.67	1.54
$\omega_{22}(b_u)$	3358.1	117.78	75.80	71.70	0.49	−1.87	−2.89	122.93	80.52	75.48	2.66	1.44
$\omega_{23}(b_u)$	3008.5	148.40	127.68	118.37	14.16	16.64	15.18	139.64	117.82	107.38	−1.56	1.66
$\omega_{24}(b_u)$	1769.5	83.62	51.99	47.93	17.13	5.82	5.36	74.30	45.80	41.30	10.15	0.68
$\omega_{25}(b_u)$	1652.0	6.78	16.35	9.22	−5.83	−7.55	−6.62	11.21	22.92	15.72	−0.24	−1.26
$\omega_{26}(b_u)$	1417.3	35.78	22.73	20.26	8.67	5.72	4.42	30.60	18.43	15.45	4.32	1.44
$\omega_{27}(b_u)$	1334.1	36.26	27.23	26.14	3.68	−0.82	−0.56	34.55	27.38	25.90	3.81	0.12
$\omega_{28}(b_u)$	1094.8	14.51	12.47	13.11	−3.41	−3.63	−3.94	15.32	15.54	16.67	−0.96	−0.47
$\omega_{29}(b_u)$	627.8	16.46	4.94	4.45	4.69	−0.48	−0.95	14.86	4.63	4.31	5.00	0.08
$\omega_{30}(b_u)$	227.6	3.80	1.35	−0.03	−4.72	−3.20	−5.27	7.66	6.43	5.70	−0.66	1.01

Natural Internal Coordinates

Table S25: Symmetrized, unnormalized natural internal coordinates for formamide dimer.

1	$r_{1,2} + r_{7,8}$
2	$r_{1,2} - r_{7,8}$
3	$r_{1,3} + r_{7,9}$
4	$r_{1,3} - r_{7,9}$
5	$r_{3,4} + r_{3,5} + r_{9,10} + r_{9,11}$
6	$r_{3,4} + r_{3,5} - r_{9,10} - r_{9,11}$
7	$r_{3,4} - r_{3,5} + r_{9,10} - r_{9,11}$
8	$r_{3,4} - r_{3,5} - r_{9,10} + r_{9,11}$
9	$r_{1,6} + r_{7,12}$
10	$r_{1,6} - r_{7,12}$
11	$r(2,5\|8,11)$
12	$\phi_{3,1,2} + \phi_{9,7,8}$
13	$\phi_{3,1,2} - \phi_{9,7,8}$
14	$\phi_{2,1,6} - \phi_{3,1,6} + \phi_{8,7,12} - \phi_{9,7,12}$
15	$\phi_{2,1,6} - \phi_{3,1,6} - \phi_{8,7,12} + \phi_{9,7,12}$
16	$2\phi_{4,3,5} - \phi_{4,3,1} - \phi_{5,3,1} + 2\phi_{10,9,11} - \phi_{10,9,7} - \phi_{11,9,7}$
17	$2\phi_{4,3,5} - \phi_{4,3,1} - \phi_{5,3,1} - 2\phi_{10,9,11} + \phi_{10,9,7} + \phi_{11,9,7}$
18	$\phi_{4,3,1} - \phi_{5,3,1} + \phi_{10,9,7} - \phi_{11,9,7}$
19	$\phi_{4,3,1} - \phi_{5,3,1} - \phi_{10,9,7} + \phi_{11,9,7}$
20	$\phi(3,4\|2,5\|8,11) + \phi(2,5\|8,11\|9,10)$
21	$\phi(3,4\|2,5\|8,11) - \phi(2,5\|8,11\|9,10)$
22	$\tau_{2,1,3,4} + \tau_{2,1,3,5} + \tau_{8,7,9,10} + \tau_{8,7,9,11}$
23	$\tau_{2,1,3,4} + \tau_{2,1,3,5} - \tau_{8,7,9,10} - \tau_{8,7,9,11}$
24	$\tau(3,4\|2,5\|8,11\|9,10)$
25	$\tau(1,6\|3,4\|2,5\|8,11) + \tau(2,5\|8,11\|9,10\|7,12)$
26	$\tau(1,6\|3,4\|2,5\|8,11) - \tau(2,5\|8,11\|9,10\|7,12)$
27	$\gamma_{6,1,2,3} + \gamma_{12,7,8,9}$
28	$\gamma_{6,1,2,3} - \gamma_{12,7,8,9}$
29	$\gamma_{1,3,4,5} + \gamma_{7,9,10,11}$
30	$\gamma_{1,3,4,5} - \gamma_{7,9,10,11}$

S3.7 water−acetylene dimer

Structure

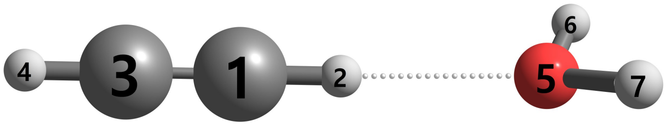

Figure S7: Water-acetylene dimer structure labeled with atomic indices.

Literature Comparison

Table S26: A comparison of key geometric features to the highest available level of theory Literature geometric structure. Units are either Å or °, depending on the coordinate type.

Geometric Parameter	This work CCSD(T)/aug-cc-pVTZ	Xantheas CCSD(T)/aug-cc-pVTZ[7]	Difference
$r_{H_4C_3}$	1.0643	1.0643	0.0000
$r_{C_3C_1}$	1.2115	1.2115	0.0000
$r_{C_1H_2}$	1.0687	1.0686	0.0001
$r_{O_5H_6}$	0.9619	0.9618	0.0001
$\theta_{H_4C_3C_1}$	179.93	179.98	−0.05
$\theta_{C_3C_1H_2}$	179.90	179.96	−0.06
$\theta_{H_6O_5H_7}$	104.51	104.55	−0.04
$r_{H_2 \cdots O_5}$	2.1925	2.1895	0.0030
$\gamma_{H_2 \cdots O_5H_6H_7}$	30.04	23.98	6.06

Frequencies

Table S27: CMA-0A residuals with respect to the reference harmonic frequencies (in cm^{-1}) for $H_2O \cdots C_2H_2$ using natural internal coordinates with cartesian force constants.

	Reference	CMA-0A										
	CCSD(T)	MP2						CCSD(T)				
	aTZ	DZ	haDZ	aDZ	TZ	haTZ	aTZ	DZ	haDZ	aDZ	TZ	haTZ
$\omega_1(a')$	3809.7	0.0	0.0	0.0	0.0	0.0	0.0	0.0	0.0	0.0	0.0	0.0
$\omega_2(a')$	3479.2	−1.3	−1.1	−0.2	−0.7	−1.2	−0.1	−0.6	−0.5	−0.1	−0.2	−0.6
$\omega_3(a')$	3360.0	1.3	1.1	0.2	0.7	1.2	0.1	0.6	0.5	0.0	0.2	0.6
$\omega_4(a')$	1985.6	0.0	0.0	0.0	0.0	0.0	0.0	0.0	0.0	0.1	0.0	0.0
$\omega_5(a')$	1646.9	0.0	0.0	0.0	0.0	0.0	0.0	0.0	0.1	0.1	0.0	0.0
$\omega_6(a')$	808.7	−0.8	−0.7	−0.7	−0.1	−0.2	0.0	−0.5	−0.9	−0.9	−0.1	−0.2
$\omega_7(a')$	621.6	0.9	0.9	0.3	0.0	0.2	0.0	0.5	1.1	0.1	0.0	0.3
$\omega_8(a')$	126.4	−8.0	−1.0	2.0	−2.7	−0.8	0.0	−4.1	−1.0	1.8	−1.9	−0.5
$\omega_9(a')$	122.7	−4.6	0.6	0.2	0.8	0.2	0.0	−8.4	0.7	2.6	0.5	0.2
$\omega_{10}(a')$	78.2	18.5	1.0	1.3	3.5	1.2	0.1	18.5	0.8	1.7	2.7	0.7
$\omega_{11}(a'')$	3917.4	0.0	0.0	0.0	0.0	0.0	0.0	0.0	0.0	0.0	0.0	0.0
$\omega_{12}(a'')$	826.8	−2.7	−0.6	−0.9	−1.0	−0.1	0.0	−2.1	−0.9	−1.2	−0.7	−0.2
$\omega_{13}(a'')$	627.1	3.3	0.7	0.3	1.2	0.1	0.0	2.4	1.0	−0.2	0.8	0.2
$\omega_{14}(a'')$	194.1	−0.1	0.4	2.3	0.0	0.0	0.0	0.0	0.6	5.0	0.0	0.0
$\omega_{15}(a'')$	71.1	2.4	0.0	1.3	1.3	0.1	0.2	3.0	0.0	1.2	1.4	0.2

Table S28: Pure level B residuals with respect to the reference harmonic frequencies (in cm^{-1}) for $H_2O \cdots C_2H_2$ using natural internal coordinates with cartesian force constants.

	Reference	CMA-0A										
	CCSD(T)	MP2						CCSD(T)				
	aTZ	DZ	haDZ	aDZ	TZ	haTZ	aTZ	DZ	haDZ	aDZ	TZ	haTZ
$\omega_1(a')$	3809.7	80.82	56.12	44.25	10.57	8.48	6.69	71.68	49.51	37.72	2.52	1.80
$\omega_2(a')$	3479.2	155.38	139.06	129.03	16.00	9.45	10.22	148.45	131.01	120.87	5.68	−1.18
$\omega_3(a')$	3360.0	158.11	139.42	136.52	18.94	15.79	11.06	152.50	131.93	128.16	8.67	5.75
$\omega_4(a')$	1985.6	83.60	79.52	73.70	−14.17	−15.24	−17.63	98.58	94.72	88.95	3.56	2.51
$\omega_5(a')$	1646.9	1.51	−4.94	−16.34	−7.40	−17.68	−18.86	17.40	12.84	1.44	10.82	1.06
$\omega_6(a')$	808.7	48.31	−18.28	−10.60	3.28	−7.02	4.46	34.92	−27.58	−20.01	−1.46	−11.84
$\omega_7(a')$	621.6	35.08	−20.98	−102.56	−2.95	11.67	9.97	9.68	−44.87	−138.41	−14.96	0.03
$\omega_8(a')$	126.4	−8.15	8.86	4.92	−6.65	3.65	1.58	−6.52	7.60	4.31	−4.94	1.38
$\omega_9(a')$	122.7	−8.66	−2.10	4.40	−10.11	−3.10	−1.89	−7.46	0.01	5.11	−8.30	−0.96
$\omega_{10}(a')$	78.2	−166.26	9.32	6.24	−15.05	−0.86	1.38	−163.60	7.90	4.25	−12.32	−3.06
$\omega_{11}(a'')$	3917.4	108.52	79.44	70.47	25.99	26.45	23.31	87.26	57.23	48.08	1.75	3.16
$\omega_{12}(a'')$	826.8	79.05	−17.80	−16.44	33.78	−7.28	4.66	64.66	−27.70	−25.80	26.02	−12.40
$\omega_{13}(a'')$	627.1	40.50	−20.19	−99.74	9.66	10.88	9.50	16.46	−43.47	−133.63	−2.35	−0.35
$\omega_{14}(a'')$	194.1	29.35	2.53	−21.04	17.36	−0.98	−2.99	27.66	1.06	−22.56	16.54	0.44
$\omega_{15}(a'')$	71.1	21.68	2.27	0.76	7.26	−9.43	−4.13	21.65	3.86	2.02	7.68	−7.32

Natural Internal Coordinates

Table S29: Symmetrized, unnormalized natural internal coordinates for water$\cdots$acetylene.

1	$r_{1,3}$
2	$r_{1,2} + r_{3,4}$
3	$r_{1,2} - r_{3,4}$
4	$r_{5,6} + r_{5,7}$
5	$r_{5,6} - r_{5,7}$
6	$r_{2,5}$
7	$\phi_{6,5,7}$
8	$\phi(2\|5\|6,7)$
9	$\tau(2\|5\|6,7\|6) + \tau(2\|5\|6,7\|7)$
10	$\gamma_{5,2,1,8}$
11	$\gamma_{5,2,1,9}$
12	$\theta_{3,1,2,8} + \theta_{1,3,4,10}$
13	$\theta_{3,1,2,8} - \theta_{1,3,4,10}$
14	$\theta_{3,1,2,9} + \theta_{1,3,4,11}$
15	$\theta_{3,1,2,9} - \theta_{1,3,4,11}$

S3.8 water – acetic acid dimer

Structure

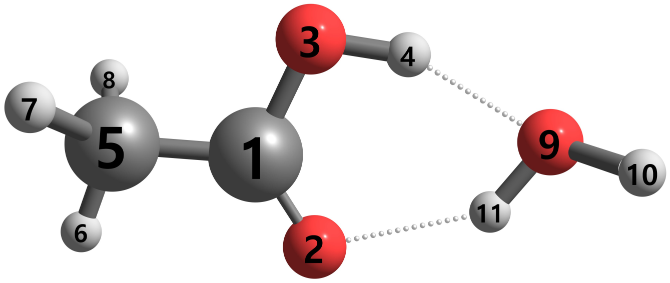

Figure S8: Water-acetic acid dimer structure labeled with atomic indices.

Frequencies

Table S30: CMA-0A residuals with respect to the reference harmonic frequencies (in cm^{-1}) for $CH_3COOH\cdots H_2O$ using natural internal coordinates with cartesian force constants.

	Reference	CMA-0A
	CCSD(T)	MP2
	aTZ	haTZ
$\omega_1(a)$	3880.35	−0.1
$\omega_2(a)$	3650.80	−0.1
$\omega_3(a)$	3452.24	0.0
$\omega_4(a)$	3173.89	0.0
$\omega_5(a)$	3131.10	0.0
$\omega_6(a)$	3058.60	0.0
$\omega_7(a)$	1778.73	−0.2
$\omega_8(a)$	1647.74	0.2
$\omega_9(a)$	1491.39	0.0
$\omega_{10}(a)$	1485.83	−0.4
$\omega_{11}(a)$	1459.26	0.5
$\omega_{12}(a)$	1401.51	0.0
$\omega_{13}(a)$	1302.08	0.1
$\omega_{14}(a)$	1076.21	0.0
$\omega_{15}(a)$	1025.04	0.1
$\omega_{16}(a)$	894.75	−0.7
$\omega_{17}(a)$	886.08	0.7
$\omega_{18}(a)$	654.80	0.1
$\omega_{19}(a)$	615.56	0.1
$\omega_{20}(a)$	594.81	0.0
$\omega_{21}(a)$	439.11	0.0
$\omega_{22}(a)$	365.72	0.6
$\omega_{23}(a)$	259.98	0.2
$\omega_{24}(a)$	196.64	0.0
$\omega_{25}(a)$	175.70	0.1
$\omega_{26}(a)$	88.61	0.2
$\omega_{27}(a)$	64.02	0.4

Table S31: Pure level B residuals with respect to the reference harmonic frequencies (in cm^{-1}) for $CH_3COOH\cdots H_2O$ using natural internal coordinates with cartesian force constants.

	Reference	Pure
	CCSD(T)	MP2
	aTZ	haTZ
$\omega_1(a)$	3880.35	19.36
$\omega_2(a)$	3650.80	0.50
$\omega_3(a)$	3452.24	−3.41
$\omega_4(a)$	3173.89	19.33
$\omega_5(a)$	3131.10	20.46
$\omega_6(a)$	3058.60	9.44
$\omega_7(a)$	1778.73	−2.07
$\omega_8(a)$	1647.74	−16.77
$\omega_9(a)$	1491.39	1.93
$\omega_{10}(a)$	1485.83	−0.67
$\omega_{11}(a)$	1459.26	−8.67
$\omega_{12}(a)$	1401.51	−9.61
$\omega_{13}(a)$	1302.08	−7.34
$\omega_{14}(a)$	1076.21	−2.97
$\omega_{15}(a)$	1025.04	−1.54
$\omega_{16}(a)$	894.75	2.37
$\omega_{17}(a)$	886.08	2.12
$\omega_{18}(a)$	654.80	−1.70
$\omega_{19}(a)$	615.56	−0.78
$\omega_{20}(a)$	594.81	0.73
$\omega_{21}(a)$	439.11	0.33
$\omega_{22}(a)$	365.72	−0.20
$\omega_{23}(a)$	259.98	2.46
$\omega_{24}(a)$	196.64	−4.48
$\omega_{25}(a)$	175.70	−3.45
$\omega_{26}(a)$	88.61	−0.83
$\omega_{27}(a)$	64.02	−3.29

Natural Internal Coordinates

Table S32: Symmetrized, unnormalized natural internal coordinates for acetic acid–water dimer.

1	$r_{3,4} + r_{4,9} + r_{9,11} + r_{11,2} + r_{2,1} + r_{1,3}$
2	$r_{3,4} - r_{4,9} + r_{9,11} - r_{11,2} + r_{2,1} - r_{1,3}$
3	$2r_{3,4} + r_{4,9} - r_{9,11} - 2r_{11,2} - r_{2,1} + r_{1,3}$
4	$r_{4,9} + r_{9,11} - r_{2,1} - r_{1,3}$
5	$2r_{3,4} - r_{4,9} - r_{9,11} + 2r_{11,2} - r_{2,1} - r_{1,3}$
6	$r_{4,9} - r_{9,11} + r_{2,1} - r_{1,3}$
7	$r_{1,5}$
8	$r_{5,6} + r_{5,7} + r_{5,8}$
9	$2r_{5,6} - r_{5,7} - r_{5,8}$
10	$r_{5,7} - r_{5,8}$
11	$r_{9,10}$
12	$\phi_{1,3,4} - \phi_{3,4,9} + \phi_{4,9,11} - \phi_{9,11,2} + \phi_{11,2,1} - \phi_{2,1,3}$
13	$2\phi_{1,3,4} - \phi_{3,4,9} - \phi_{4,9,11} + 2\phi_{9,11,2} - \phi_{11,2,1} - \phi_{2,1,3}$
14	$\phi_{3,4,9} - \phi_{4,9,11} + \phi_{11,2,1} - \phi_{2,1,3}$
15	$\phi_{5,1,3} - \phi_{5,1,2}$
16	$\phi_{10,9,11} + \phi_{10,9,4}$
17	$\phi_{10,9,11} - \phi_{10,9,4}$
18	$\phi_{6,5,1} + \phi_{7,5,1} + \phi_{8,5,1} - \phi_{7,5,8} - \phi_{7,5,6} - \phi_{8,5,6}$
19	$2\phi_{6,5,1} - \phi_{7,5,1} - \phi_{8,5,1}$
20	$\phi_{7,5,1} - \phi_{8,5,1}$
21	$2\phi_{7,5,8} - \phi_{7,5,6} - \phi_{8,5,6}$
22	$\phi_{7,5,6} - \phi_{8,5,6}$
23	$\tau_{2,1,3,4} - \tau_{1,3,4,9} + \tau_{3,4,9,11} - \tau_{4,9,11,2} + \tau_{9,11,2,1} - \tau_{11,2,1,3}$
24	$\tau_{2,1,3,4} - \tau_{3,4,9,11} + \tau_{4,9,11,2} - \tau_{11,2,1,3}$
25	$-\tau_{2,1,3,4} + 2\tau_{1,3,4,9} - \tau_{3,4,9,11} - \tau_{4,9,11,2} + 2\tau_{9,11,2,1} - \tau_{11,2,1,3}$
26	$\tau_{6,5,1,2} + \tau_{7,5,1,2} + \tau_{8,5,1,2}$
27	$\gamma_{5,1,3,2}$

S3.9 hydrofluoric acid dimer

Structure

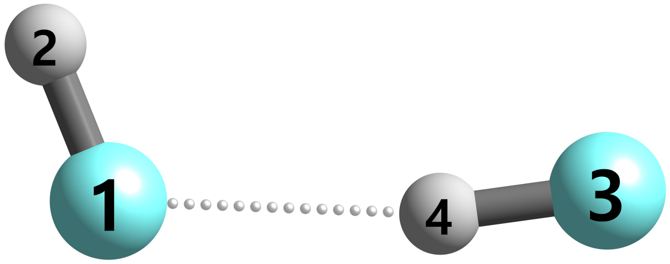

Figure S9: Hydrofluoric acid dimer structure labeled with atomic indices.

Literature Comparison

Table S33: A comparison of key geometric features to the highest available level of theory Literature geometric structure. Units are either Å or °, depending on the coordinate type.

Geometric Parameter	This work CCSD(T)/aug-cc-pVTZ	Tschumper CCSD(T)/h-aug-cc-pV5Z[8]	Difference
$r_{H_2F_1}$	0.9239	0.9202	−0.0037
$r_{H_4F_3}$	0.9265	0.9230	−0.0036
$r_{H_4 \cdots F_1}$	1.8246	1.8241	−0.0005
$\theta_{H_2F_1 \cdots H_4}$	114.16	114.91	0.75
$\theta_{F_1 \cdots H_4F_3}$	169.97	169.56	−0.40

Frequencies

Table S34: CMA-0A residuals with respect to the reference harmonic frequencies (in cm^{-1}) for $(HF)_2$ using natural internal coordinates with cartesian force constants.

	Reference	CMA-0A										
	CCSD(T)	MP2						CCSD(T)				
	aTZ	DZ	haDZ	aDZ	TZ	haTZ	aTZ	DZ	haDZ	aDZ	TZ	haTZ
$\omega_1(a')$	4088.4	−0.3	0.0	0.0	−0.1	0.0	0.0	−0.4	0.0	−0.2	−0.1	0.0
$\omega_2(a')$	4009.2	0.3	−0.1	0.0	0.0	−0.1	−0.1	0.3	0.0	−0.2	0.0	−0.1
$\omega_3(a')$	576.0	−0.1	−0.4	0.0	0.0	0.0	0.0	−0.1	0.0	0.2	−0.1	0.0
$\omega_4(a')$	218.2	−0.5	0.9	−0.5	−0.4	0.1	0.0	−1.2	−0.8	−0.2	−0.6	0.0
$\omega_5(a')$	161.2	1.3	0.2	0.9	0.8	0.1	0.1	2.3	1.3	1.6	1.0	0.1
$\omega_6(a'')$	471.7	0.5	0.5	0.5	0.5	0.5	0.5	0.5	0.5	0.4	0.6	0.5

Table S35: Pure level B residuals with respect to the reference harmonic frequencies (in cm^{-1}) for $(HF)_2$ using natural internal coordinates with cartesian force constants.

	Reference	Pure										
	CCSD(T)	MP2						CCSD(T)				
	aTZ	DZ	haDZ	aDZ	TZ	haTZ	aTZ	DZ	haDZ	aDZ	TZ	haTZ
$\omega_1(a')$	4088.4	34.29	13.55	9.40	10.65	8.30	8.80	20.87	2.87	−0.83	−0.45	−0.49
$\omega_2(a')$	4009.2	36.35	3.76	0.10	9.58	3.42	0.43	32.86	1.35	−1.93	6.48	2.82
$\omega_3(a')$	576.0	16.46	17.87	1.90	−2.65	8.19	4.26	13.38	15.01	−2.01	−6.95	4.17
$\omega_4(a')$	218.2	−9.68	8.54	−0.40	−8.29	4.48	2.54	−6.82	9.61	−1.37	−7.62	4.24
$\omega_5(a')$	161.2	−7.51	4.49	0.34	−8.90	0.14	−0.06	−5.71	5.40	0.33	−9.58	0.27
$\omega_6(a'')$	471.7	68.87	16.62	−9.88	27.73	7.56	−1.87	64.12	17.71	−7.74	27.34	10.44

Natural Internal Coordinates

Table S36: Symmetrized, unnormalized natural internal coordinates for hf dimer.

1	$r_{1,2}$
2	$r_{1,4}$
3	$r_{3,4}$
4	$\phi_{2,1,4}$
5	$\phi_{1,4,3}$
6	$\tau_{2,1,4,3}$

S3.10 water trimer

Structure

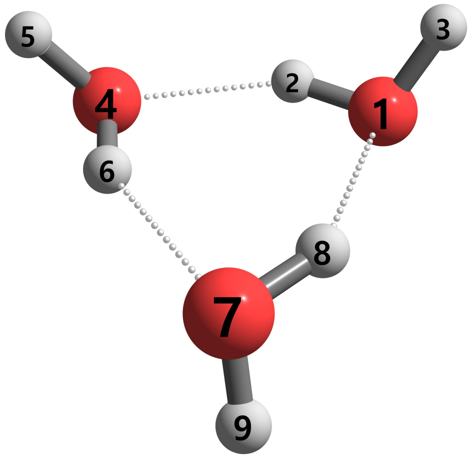

Figure S10: Water trimer structure labeled with atomic indices.

Literature Comparison

Table S37: A comparison of key geometric features to the highest available level of theory Literature geometric structure. Units are either Å or °, depending on the coordinate type.

Geometric Parameter	This work CCSD(T)/aug-cc-pVTZ	Tschumper (T):MP2/haQZ[9]	Difference
$r_{O_1H_2}$	0.9728	0.9700	0.0028
$r_{O_1H_3}$	0.9609	0.9582	0.0027
$r_{O_4H_6}$	0.9734	0.9707	0.0028
$r_{O_4H_5}$	0.9608	0.9581	0.0027
$r_{O_7H_8}$	0.9733	0.9706	0.0027
$r_{O_7H_9}$	0.9612	0.9584	0.0027
$\theta_{H_2O_1H_3}$	105.53	105.72	−0.19
$\theta_{H_5O_4H_6}$	105.44	105.67	−0.23
$\theta_{H_8O_7H_9}$	105.27	105.46	−0.19
$r_{H_2 \cdots O_4}$	1.9253	1.9251	0.0002
$r_{H_6 \cdots O_7}$	1.9055	1.9054	0.0001
$r_{H_8 \cdots O_1}$	1.9046	1.9038	0.0008
$\theta_{O_1H_2 \cdots O_4}$	148.97	148.48	−0.49
$\theta_{O_4H_6 \cdots O_7}$	151.59	150.96	−0.53
$\theta_{O_7H_8 \cdots O_1}$	151.34	150.99	−0.35
$\gamma_{H_2 \cdots O_4H_5H_6}$	49.17	47.18	1.99
$\gamma_{H_6 \cdots O_7H_8H_9}$	56.08	54.81	1.27
$\gamma_{H_8 \cdots O_1H_3H_2}$	51.81	50.30	1.51

Literature Comparison

Table S38: A comparison of key geometric features to the highest available level of theory Literature geometric structure. Units are either Å or °, depending on the coordinate type.

Geometric Parameter	This work CCSD(T)/h-aug-cc-pVTZ	Tschumper (T):MP2/haTZ []	Tschumper MP2/haTZ
$r_{O_1H_2}$	0.9727	0.9727	0.9740
$r_{O_1H_3}$	0.9608	0.9608	0.9607
$r_{O_4H_6}$	0.9733	0.9733	0.9747
$r_{O_4H_5}$	0.9608	0.9608	0.9606
$r_{O_7H_8}$	0.9732	0.9732	0.9746
$r_{O_7H_9}$	0.9611	0.9611	0.9609
$\theta_{H_2O_1H_3}$	105.53	105.55	105.55
$\theta_{H_5O_4H_6}$	105.44	105.47	105.47
$\theta_{H_8O_7H_9}$	105.27	105.27	105.27
$r_{H_2 \cdots O_4}$	1.9269	1.9249	1.9133
$r_{H_6 \cdots O_7}$	1.9065	1.9042	1.8936
$r_{H_8 \cdots O_1}$	1.9052	1.9030	1.8920
$\theta_{O_1H_2 \cdots O_4}$	148.97	148.49	148.46
$\theta_{O_4H_6 \cdots O_7}$	151.49	151.19	151.12
$\theta_{O_7H_8 \cdots O_1}$	151.34	151.09	151.11
$\gamma_{H_2 \cdots O_4H_5H_6}$	49.17	48.16	47.33
$\gamma_{H_6 \cdots O_7H_8H_9}$	56.08	55.71	54.87
$\gamma_{H_8 \cdots O_1H_3H_2}$	51.81	51.18	50.49

Frequencies

Table S39: CMA-0A residuals with respect to the reference harmonic frequencies (in cm^{-1}) for $(H_2O)_3$ using natural internal coordinates with cartesian force constants.

	Reference	CMA-0A										
	CCSD(T)	MP2						CCSD(T)				
	aTZ	DZ	haDZ	aDZ	TZ	haTZ	aTZ	DZ	haDZ	aDZ	TZ	haTZ
$\omega_1(a)$	3887.53	−0.7	−0.2	−0.3	−0.3	−0.2	−0.1	−0.6	−0.1	−0.2	−0.1	0.0
$\omega_2(a)$	3886.84	−1.0	−0.2	−0.3	−0.1	−0.1	−0.1	−0.7	−0.1	−0.2	−0.1	0.0
$\omega_3(a)$	3882.83	−0.1	−0.1	−0.2	0.2	0.1	0.1	−0.4	−0.1	−0.2	0.1	0.0
$\omega_4(a)$	3669.43	0.6	0.2	0.2	0.1	0.1	0.0	0.6	0.1	0.0	0.0	0.0
$\omega_5(a)$	3662.01	0.7	0.2	0.2	0.1	0.1	0.0	0.7	0.1	−0.1	0.0	0.0
$\omega_6(a)$	3605.15	0.6	0.2	0.3	0.1	0.1	0.1	0.5	0.0	−0.1	0.0	0.0
$\omega_7(a)$	1681.57	−1.3	0.1	0.1	0.0	0.1	0.1	−1.5	0.1	0.1	0.0	0.1
$\omega_8(a)$	1659.90	1.1	0.3	0.5	0.4	0.4	0.4	1.3	0.3	0.6	0.4	0.4
$\omega_9(a)$	1657.87	0.1	−0.3	−0.5	−0.4	−0.5	−0.5	0.1	−0.3	−0.5	−0.4	−0.5
$\omega_{10}(a)$	854.91	−1.2	−0.1	0.1	−0.1	0.0	0.0	−1.1	0.0	0.4	−0.2	0.0
$\omega_{11}(a)$	654.79	−1.6	−0.1	0.0	−0.3	0.0	0.0	−1.6	−0.1	0.3	−0.4	0.0
$\omega_{12}(a)$	565.25	−1.6	−0.1	0.0	−0.2	0.0	0.0	−1.7	−0.1	0.1	−0.4	0.0
$\omega_{13}(a)$	438.37	2.2	0.0	0.0	0.0	0.0	0.0	2.0	0.1	0.4	−0.1	0.0
$\omega_{14}(a)$	352.42	−4.0	0.3	−0.3	−0.4	0.1	0.1	−4.0	0.1	0.4	−0.6	0.0
$\omega_{15}(a)$	340.20	5.2	0.1	−0.1	0.4	0.0	0.0	5.1	0.0	0.1	0.4	0.0
$\omega_{16}(a)$	238.74	1.4	−0.4	0.3	0.4	0.1	0.1	1.1	−0.5	0.3	0.4	0.1
$\omega_{17}(a)$	218.29	0.0	0.4	0.2	0.1	−0.1	0.0	0.6	0.5	1.2	0.1	0.0
$\omega_{18}(a)$	196.32	−6.6	0.5	−1.9	−0.1	−0.2	−0.2	−5.7	0.4	−2.8	0.0	0.1
$\omega_{19}(a)$	186.63	2.8	−0.3	−0.2	−0.4	−1.0	−0.4	2.0	0.0	0.3	−0.4	−0.2
$\omega_{20}(a)$	180.27	7.9	0.7	2.5	1.4	0.3	0.3	8.1	0.8	6.0	1.8	0.1
$\omega_{21}(a)$	170.52	7.5	0.8	1.1	3.1	1.7	0.8	7.5	0.5	2.8	3.5	0.4

Table S40: Pure level B residuals with respect to the reference harmonic frequencies (in cm^{-1}) for $(H_2O)_3$ using natural internal coordinates with cartesian force constants.

	Reference	Pure										
	CCSD(T)	MP2						CCSD(T)				
	aTZ	DZ	haDZ	aDZ	TZ	haTZ	aTZ	DZ	haDZ	aDZ	TZ	haTZ
$\omega_1(a)$	3887.53	97.44	71.76	62.76	18.93	20.56	17.96	80.18	54.39	45.26	−0.07	2.61
$\omega_2(a)$	3886.84	96.86	71.77	62.76	18.30	20.42	17.83	79.79	54.51	45.38	−0.29	2.74
$\omega_3(a)$	3882.83	98.93	73.40	64.48	19.75	21.76	19.07	82.41	56.40	47.31	1.37	4.30
$\omega_4(a)$	3669.43	91.16	45.06	37.55	6.69	0.47	−2.36	92.40	47.62	40.08	7.44	2.93
$\omega_5(a)$	3662.01	92.06	44.64	36.91	6.70	0.04	−2.77	93.76	47.71	39.94	7.91	3.00
$\omega_6(a)$	3605.15	80.97	42.82	36.50	−2.62	−4.44	−7.38	88.58	50.48	44.14	3.79	3.19
$\omega_7(a)$	1681.57	9.04	−0.58	−15.29	−5.69	−14.61	−16.29	22.20	14.71	−0.10	10.27	1.82
$\omega_8(a)$	1659.90	17.04	−2.23	−18.02	−3.94	−15.35	−17.64	31.15	14.88	−1.15	13.36	2.75
$\omega_9(a)$	1657.87	14.62	−2.47	−18.48	−4.80	−15.71	−17.97	29.09	14.49	−1.69	12.58	2.25
$\omega_{10}(a)$	854.91	16.95	14.58	16.23	16.16	4.36	4.52	10.59	9.75	12.39	10.42	−0.29
$\omega_{11}(a)$	654.79	21.84	18.47	13.63	12.15	5.88	3.59	18.31	14.69	10.49	8.56	2.39
$\omega_{12}(a)$	565.25	−16.98	10.18	15.76	1.76	6.39	5.93	−19.09	4.76	11.41	−2.56	0.50
$\omega_{13}(a)$	438.37	30.50	7.76	2.29	13.51	3.32	1.45	28.12	6.12	1.26	11.55	1.99
$\omega_{14}(a)$	352.42	8.44	6.99	−3.90	2.21	1.66	0.63	7.71	5.80	−5.08	1.30	0.79
$\omega_{15}(a)$	340.20	11.12	6.20	−3.51	4.64	1.25	0.45	10.45	5.73	−3.54	4.11	1.12
$\omega_{16}(a)$	238.74	−6.98	9.26	−2.77	−1.60	2.66	0.21	−7.58	7.51	−2.15	−2.00	2.19
$\omega_{17}(a)$	218.29	−3.19	0.87	0.41	−6.99	−5.73	−6.36	3.22	7.47	7.29	−1.35	0.47
$\omega_{18}(a)$	196.32	−1.97	3.45	−5.98	−3.81	0.58	−1.61	1.55	3.07	−2.59	−2.81	0.67
$\omega_{19}(a)$	186.63	5.00	3.11	−0.42	2.66	0.05	−1.94	8.19	5.72	3.61	4.89	2.06
$\omega_{20}(a)$	180.27	−9.42	0.58	−10.01	−2.47	−1.65	−2.87	−7.69	2.27	−10.35	0.17	0.89
$\omega_{21}(a)$	170.52	−17.46	−0.42	−12.67	−1.20	1.03	−0.73	−15.75	−1.07	−12.89	−0.37	2.09

Natural Internal Coordinates

Table S41: Symmetrized, unnormalized natural internal coordinates for water trimer.

1	$r_{1,2} + r_{2,4} + r_{4,6} + r_{6,7} + r_{7,8} + r_{8,1}$
2	$r_{1,2} - r_{2,4} + r_{4,6} - r_{6,7} + r_{7,8} - r_{8,1}$
3	$2r_{1,2} + r_{2,4} - r_{4,6} - 2r_{6,7} - r_{7,8} + r_{8,1}$
4	$r_{2,4} + r_{4,6} - r_{7,8} - r_{8,1}$
5	$2r_{1,2} - r_{2,4} - r_{4,6} + 2r_{6,7} - r_{7,8} - r_{8,1}$
6	$r_{2,4} - r_{4,6} + r_{7,8} - r_{8,1}$
7	$r_{4,5}$
8	$r_{1,3}$
9	$r_{7,9}$
10	$\phi_{2,1,3} + \phi_{8,1,3}$
11	$\phi_{2,1,3} - \phi_{8,1,3}$
12	$\phi_{5,4,2} + \phi_{5,4,6}$
13	$\phi_{5,4,2} - \phi_{5,4,6}$
14	$\phi_{6,7,9} + \phi_{8,7,9}$
15	$\phi_{6,7,9} - \phi_{8,7,9}$
16	$\phi_{2,4,6} - \phi_{4,6,7} + \phi_{6,7,8} - \phi_{7,8,1} + \phi_{8,1,2} - \phi_{1,2,4}$
17	$2\phi_{2,4,6} - \phi_{4,6,7} - \phi_{6,7,8} + 2\phi_{7,8,1} - \phi_{8,1,2} - \phi_{1,2,4}$
18	$\phi_{4,6,7} - \phi_{6,7,8} + \phi_{8,1,2} - \phi_{1,2,4}$
19	$\tau_{2,4,6,7} - \tau_{4,6,7,8} + \tau_{6,7,8,1} - \tau_{7,8,1,2} + \tau_{8,1,2,4} - \tau_{1,2,4,6}$
20	$\tau_{2,4,6,7} - \tau_{6,7,8,1} + \tau_{7,8,1,2} - \tau_{1,2,4,6}$
21	$-\tau_{2,4,6,7} + 2\tau_{4,6,7,8} - \tau_{6,7,8,1} - \tau_{7,8,1,2} + 2\tau_{8,1,2,4} - \tau_{1,2,4,6}$

S3.11 ethylene dimer

Structure

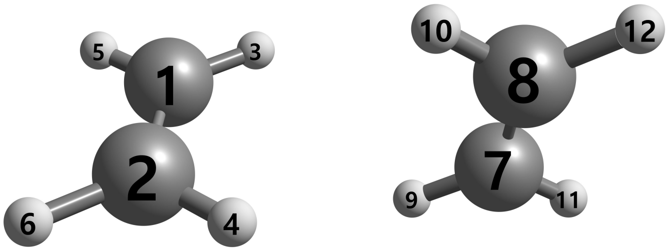

Figure S11: Ethylene dimer structure labeled with atomic indices.

Frequencies

Table S42: CMA-0A residuals with respect to the reference harmonic frequencies (in cm^{-1}) for $(C_2H_4)_2$ using natural internal coordinates with cartesian force constants.

	Reference	CMA-0A										
	CCSD(T)	MP2						CCSD(T)				
	aTZ	DZ	haDZ	aDZ	TZ	haTZ	aTZ	DZ	haDZ	aDZ	TZ	haTZ
$\omega_1(a_1)$	3237.8	−0.06	−0.01	−0.03	0.00	−0.00	−0.00	−0.07	−0.01	−0.01	−0.00	0.00
$\omega_2(a_1)$	3148.2	0.00	−0.05	−0.03	−0.01	−0.01	−0.00	0.04	−0.03	−0.02	0.00	−0.00
$\omega_3(a_1)$	1666.5	−1.47	−1.26	−1.33	0.00	0.00	0.00	−1.23	−1.10	−1.21	0.00	0.01
$\omega_4(a_1)$	1369.6	1.91	1.69	1.77	0.02	0.02	0.01	1.58	1.47	1.58	0.01	0.00
$\omega_5(a_1)$	825.7	0.08	0.04	0.06	0.02	0.02	0.02	0.05	0.02	0.03	0.00	0.01
$\omega_6(a_1)$	79.4	0.02	0.05	0.04	0.00	0.01	0.00	0.04	0.08	0.05	0.00	0.00
$\omega_7(a_2)$	936.0	−0.00	0.00	0.00	0.00	0.00	0.00	−0.00	0.00	−0.00	0.00	−0.00
$\omega_8(b_1)$	943.2	−0.00	−0.00	−0.00	−0.00	−0.00	−0.00	−0.00	−0.00	−0.00	0.00	−0.00
$\omega_9(b_1)$	74.1	0.01	0.00	0.00	0.00	0.00	0.00	0.00	0.00	0.01	0.00	0.00
$\omega_{10}(b_2)$	3237.7	−0.03	−0.01	−0.04	−0.01	−0.01	−0.01	−0.02	−0.01	−0.03	−0.00	−0.00
$\omega_{11}(b_2)$	3149.2	−0.02	−0.05	−0.02	−0.01	−0.01	−0.00	−0.01	−0.04	−0.01	−0.00	−0.00
$\omega_{12}(b_2)$	1661.4	−1.28	−1.25	−1.45	0.00	0.00	0.00	−1.01	−1.02	−1.25	−0.00	0.01
$\omega_{13}(b_2)$	1360.6	1.69	1.68	1.89	0.02	0.02	0.02	1.31	1.39	1.60	0.01	0.00
$\omega_{14}(b_2)$	817.7	0.07	0.04	0.09	0.02	0.02	0.02	0.04	0.02	0.06	0.00	0.01
$\omega_{15a}(e)$	3212.0	−0.01	−0.00	−0.03	−0.00	−0.00	−0.01	−0.03	0.03	−0.02	−0.00	0.02
$\omega_{15b}(e)$	3212.0	−0.01	−0.00	−0.03	−0.00	−0.00	−0.01	−0.03	0.03	−0.02	−0.00	0.02
$\omega_{16a}(e)$	3132.0	0.01	−0.01	0.03	−0.00	0.00	0.00	0.03	−0.04	0.02	−0.00	−0.02
$\omega_{16b}(e)$	3132.0	0.01	−0.01	0.03	−0.00	0.00	0.00	0.03	−0.04	0.02	−0.00	−0.02
$\omega_{17a}(e)$	1474.5	0.01	0.01	−0.00	0.01	0.01	0.01	0.01	0.02	−0.00	0.01	0.01
$\omega_{17b}(e)$	1474.5	0.01	0.01	−0.00	0.01	0.01	0.01	0.01	0.02	−0.00	0.01	0.01
$\omega_{18a}(e)$	1242.5	−0.00	−0.00	0.01	−0.00	0.00	0.00	−0.00	−0.00	0.00	−0.00	−0.00
$\omega_{18b}(e)$	1242.5	−0.00	−0.00	0.01	−0.00	0.00	0.00	−0.00	−0.00	0.00	−0.00	−0.00
$\omega_{19a}(e)$	1036.6	0.03	0.03	0.02	0.02	0.02	0.02	0.02	0.02	0.03	0.02	0.02
$\omega_{19b}(e)$	1036.6	0.03	0.03	0.02	0.02	0.02	0.02	0.02	0.02	0.03	0.02	0.02
$\omega_{20a}(e)$	964.7	−0.02	−0.04	−0.02	−0.02	−0.02	−0.01	−0.02	−0.03	−0.02	−0.02	−0.02
$\omega_{20b}(e)$	964.7	−0.02	−0.04	−0.02	−0.02	−0.02	−0.01	−0.02	−0.03	−0.02	−0.02	−0.02
$\omega_{21a}(e)$	94.1	0.00	0.01	0.01	−0.01	0.01	0.00	0.02	0.02	0.02	−0.00	0.01
$\omega_{21b}(e)$	94.1	0.00	0.01	0.01	−0.01	0.01	0.00	0.02	0.02	0.02	−0.00	0.01
$\omega_{22a}(e)$	76.6	0.03	0.06	0.02	0.03	0.02	0.03	0.02	0.07	0.02	0.03	0.02
$\omega_{22b}(e)$	76.6	0.03	0.06	0.02	0.03	0.02	0.03	0.02	0.07	0.02	0.03	0.02

Table S43: Pure level B residuals with respect to the reference harmonic frequencies (in cm^{-1}) for $(C_2H_4)_2$ using natural internal coordinates with cartesian force constants.

	Reference	Pure										
	CCSD(T)	MP2						CCSD(T)				
	aTZ	DZ	haDZ	aDZ	TZ	haTZ	aTZ	DZ	haDZ	aDZ	TZ	haTZ
$\omega_1(a_1)$	3237.8	174.00	150.28	140.64	21.90	22.48	20.72	157.91	133.86	123.16	0.46	1.49
$\omega_2(a_1)$	3148.2	156.02	131.23	123.66	15.39	14.69	14.68	146.50	120.77	112.07	0.42	−0.13
$\omega_3(a_1)$	1666.5	54.05	49.59	44.30	−3.46	−4.47	−5.72	60.20	56.18	50.64	2.62	1.10
$\omega_4(a_1)$	1369.6	36.35	35.34	35.38	6.96	5.92	4.21	31.57	31.13	30.72	2.55	1.64
$\omega_5(a_1)$	825.7	19.49	15.97	15.23	0.05	0.29	−2.61	22.32	18.95	17.73	2.13	2.78
$\omega_6(a_1)$	79.4	7.20	4.22	2.70	−2.99	−1.95	−2.96	9.46	6.87	5.19	−0.14	0.87
$\omega_7(a_2)$	936.0	25.88	22.60	37.02	14.52	12.28	6.72	12.18	10.84	24.30	7.17	5.12
$\omega_8(b_1)$	943.2	29.37	22.32	37.36	15.76	12.38	7.19	14.83	10.11	24.46	7.81	4.82
$\omega_9(b_1)$	74.1	3.73	2.47	1.94	2.31	3.35	2.79	1.68	0.65	−0.70	−0.62	0.60
$\omega_{10}(b_2)$	3237.7	173.23	149.57	140.43	21.91	22.64	21.00	156.63	132.70	122.64	0.08	1.33
$\omega_{11}(b_2)$	3149.2	155.30	130.82	123.73	15.25	14.79	15.01	145.23	119.85	111.77	−0.17	−0.41
$\omega_{12}(b_2)$	1661.4	56.79	50.83	45.18	−2.32	−4.12	−5.42	62.51	57.05	51.19	3.46	1.20
$\omega_{13}(b_2)$	1360.6	34.22	33.06	31.33	6.35	5.39	3.71	30.24	29.50	27.38	2.64	1.61
$\omega_{14}(b_2)$	817.7	20.28	14.24	8.35	1.02	−0.33	−3.24	23.00	17.61	11.27	3.79	2.68
$\omega_{15a}(e)$	3212.0	175.04	149.23	140.13	22.00	21.92	20.64	158.70	132.70	122.58	0.32	0.81
$\omega_{15b}(e)$	3212.0	175.04	149.23	140.13	22.00	21.92	20.64	158.70	132.70	122.58	0.32	0.81
$\omega_{16a}(e)$	3132.0	154.63	129.57	123.66	16.28	15.08	15.24	143.93	118.10	111.10	0.40	−0.30
$\omega_{16b}(e)$	3132.0	154.63	129.57	123.66	16.28	15.08	15.24	143.93	118.10	111.10	0.40	−0.30
$\omega_{17a}(e)$	1474.5	18.45	18.30	16.30	4.93	3.23	0.27	19.90	19.38	16.39	5.10	2.58
$\omega_{17b}(e)$	1474.5	18.45	18.30	16.30	4.93	3.23	0.27	19.90	19.38	16.39	5.10	2.58
$\omega_{18a}(e)$	1242.5	12.52	7.20	4.86	−3.63	−2.03	−4.18	15.05	10.34	7.76	0.15	2.01
$\omega_{18b}(e)$	1242.5	12.52	7.20	4.86	−3.63	−2.03	−4.18	15.05	10.34	7.76	0.15	2.01
$\omega_{19a}(e)$	1036.6	50.84	37.86	31.70	36.71	30.97	23.02	22.99	12.92	6.95	12.89	8.28
$\omega_{19b}(e)$	1036.6	50.84	37.86	31.70	36.71	30.97	23.02	22.99	12.92	6.95	12.89	8.28
$\omega_{20a}(e)$	964.7	30.33	22.50	33.41	15.04	11.64	9.23	15.66	9.64	20.07	5.68	2.29
$\omega_{20b}(e)$	964.7	30.33	22.50	33.41	15.04	11.64	9.23	15.66	9.64	20.07	5.68	2.29
$\omega_{21a}(e)$	94.1	9.92	8.95	2.85	0.45	1.89	−1.29	11.63	10.72	4.06	1.45	3.07
$\omega_{21b}(e)$	94.1	9.92	8.95	2.85	0.45	1.89	−1.29	11.63	10.72	4.06	1.45	3.07
$\omega_{22a}(e)$	76.6	12.56	−10.58	−15.93	4.26	−5.40	0.11	8.26	−10.28	−16.53	3.55	−5.44
$\omega_{22b}(e)$	76.6	12.56	−10.58	−15.93	4.26	−5.40	0.11	8.26	−10.28	−16.53	3.55	−5.44

Natural Internal Coordinates

Table S44: Symmetrized, unnormalized natural internal coordinates for ethylene dimer.

1	$r_{1,2} + r_{7,8}$
2	$r_{1,2} - r_{7,8}$
3	$r_{1,3} + r_{1,5} + r_{2,4} + r_{2,6} + r_{7,9} + r_{7,11} + r_{8,10} + r_{8,12}$
4	$r_{1,3} + r_{1,5} - r_{2,4} - r_{2,6} + r_{7,9} + r_{7,11} - r_{8,10} - r_{8,12}$
5	$r_{1,3} - r_{1,5} + r_{2,4} - r_{2,6} + r_{7,9} - r_{7,11} + r_{8,10} - r_{8,12}$
6	$r_{1,3} - r_{1,5} - r_{2,4} + r_{2,6} + r_{7,9} - r_{7,11} - r_{8,10} + r_{8,12}$
7	$r_{1,3} + r_{1,5} + r_{2,4} + r_{2,6} - r_{7,9} - r_{7,11} - r_{8,10} - r_{8,12}$
8	$r_{1,3} + r_{1,5} - r_{2,4} - r_{2,6} - r_{7,9} - r_{7,11} + r_{8,10} + r_{8,12}$
9	$r_{1,3} - r_{1,5} + r_{2,4} - r_{2,6} - r_{7,9} + r_{7,11} - r_{8,10} + r_{8,12}$
10	$r_{1,3} - r_{1,5} - r_{2,4} + r_{2,6} - r_{7,9} + r_{7,11} + r_{8,10} - r_{8,12}$
11	$r(1,2\|7,8)$
12	$2\phi_{3,1,5} - \phi_{3,1,2} - \phi_{5,1,2} + 2\phi_{4,2,6} - \phi_{4,2,1} - \phi_{6,2,1} + 2\phi_{9,7,11} - \phi_{9,7,8} - \phi_{11,7,8} + 2\phi_{10,8,12} - \phi_{10,8,7} - \phi_{12,8,7}$
13	$2\phi_{3,1,5} - \phi_{3,1,2} - \phi_{5,1,2} - 2\phi_{4,2,6} + \phi_{4,2,1} + \phi_{6,2,1} + 2\phi_{9,7,11} - \phi_{9,7,8} - \phi_{11,7,8} - 2\phi_{10,8,12} + \phi_{10,8,7} + \phi_{12,8,7}$
14	$\phi_{3,1,2} - \phi_{5,1,2} + \phi_{4,2,1} - \phi_{6,2,1} + \phi_{9,7,8} - \phi_{11,7,8} + \phi_{10,8,7} - \phi_{12,8,7}$
15	$\phi_{3,1,2} - \phi_{5,1,2} - \phi_{4,2,1} + \phi_{6,2,1} + \phi_{9,7,8} - \phi_{11,7,8} - \phi_{10,8,7} + \phi_{12,8,7}$
16	$2\phi_{3,1,5} - \phi_{3,1,2} - \phi_{5,1,2} + 2\phi_{4,2,6} - \phi_{4,2,1} - \phi_{6,2,1} - 2\phi_{9,7,11} + \phi_{9,7,8} + \phi_{11,7,8} - 2\phi_{10,8,12} + \phi_{10,8,7} + \phi_{12,8,7}$
17	$2\phi_{3,1,5} - \phi_{3,1,2} - \phi_{5,1,2} - 2\phi_{4,2,6} + \phi_{4,2,1} + \phi_{6,2,1} - 2\phi_{9,7,11} + \phi_{9,7,8} + \phi_{11,7,8} + 2\phi_{10,8,12} - \phi_{10,8,7} - \phi_{12,8,7}$
18	$\phi_{3,1,2} - \phi_{5,1,2} + \phi_{4,2,1} - \phi_{6,2,1} - \phi_{9,7,8} + \phi_{11,7,8} - \phi_{10,8,7} + \phi_{12,8,7}$
19	$\phi_{3,1,2} - \phi_{5,1,2} - \phi_{4,2,1} + \phi_{6,2,1} - \phi_{9,7,8} + \phi_{11,7,8} + \phi_{10,8,7} - \phi_{12,8,7}$
20	$\phi(3,1,5\|1,2\|7,8) - \phi(4,2,6\|1,2\|7,8) + \phi(11,7,9\|7,8\|1,2) - \phi(10,8,12\|7,8\|1,2)$
21	$\phi(3,1,5\|1,2\|7,8) - \phi(4,2,6\|1,2\|7,8) - \phi(11,7,9\|7,8\|1,2) + \phi(10,8,12\|7,8\|1,2)$
22	$\tau_{3,1,2,4} + \tau_{3,1,2,6} + \tau_{5,1,2,4} + \tau_{5,1,2,6} + \tau_{9,7,8,10} + \tau_{9,7,8,12} + \tau_{11,7,8,10} + \tau_{11,7,8,12}$
23	$\tau_{3,1,2,4} + \tau_{3,1,2,6} + \tau_{5,1,2,4} + \tau_{5,1,2,6} - \tau_{9,7,8,10} - \tau_{9,7,8,12} - \tau_{11,7,8,10} - \tau_{11,7,8,12}$
24	$\tau(3,1,5\|1,2\|7,8\|9,7,11) + \tau(3,1,5\|1,2\|7,8\|10,8,12) + \tau(4,2,6\|1,2\|7,8\|9,7,11) + \tau(4,2,6\|1,2\|7,8\|10,8,12)$
25	$\tau(3,4\|1,3,5\|1,2\|7,8) - \tau(5,6\|4,2,6\|1,2\|7,8) + \tau(9,10\|9,7,11\|7,8\|1,2) - \tau(11,12\|10,8,12\|7,8\|1,2)$
26	$\tau(3,4\|1,3,5\|1,2\|7,8) - \tau(5,6\|4,2,6\|1,2\|7,8) - \tau(9,10\|9,7,11\|7,8\|1,2) + \tau(11,12\|10,8,12\|7,8\|1,2)$
27	$\gamma_{2,1,3,5} + \gamma_{1,2,6,4} + \gamma_{8,7,9,11} + \gamma_{7,8,12,10}$
28	$\gamma_{2,1,3,5} + \gamma_{1,2,6,4} - \gamma_{8,7,9,11} - \gamma_{7,8,12,10}$
29	$\gamma_{2,1,3,5} - \gamma_{1,2,6,4} + \gamma_{8,7,9,11} - \gamma_{7,8,12,10}$
30	$\gamma_{2,1,3,5} - \gamma_{1,2,6,4} - \gamma_{8,7,9,11} + \gamma_{7,8,12,10}$

S3.12 methane dimer

Structure

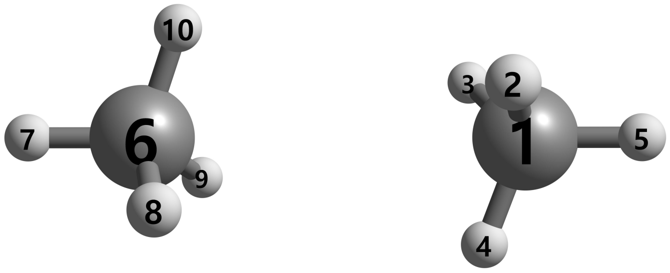

Figure S12: Methane dimer structure labeled with atomic indices.

Literature Comparison

Table S45: A comparison of key geometric features to the highest available level of theory Literature geometric structure. Units are either Å or °, depending on the coordinate type.

Geometric Parameter	This work CCSD(T)/aug-cc-pVTZ	Hobza CCSD(T)/CBS[4]	Difference
$r_{H_5C_1}$	1.0900	1.0883	0.0017
$r_{H_2C_1}$	1.0900	1.0882	0.0018
$\theta_{H_2C_1H_5}$	109.56	109.56	0.00
$r_{C_6 \cdots C_1}$	3.6214	3.6380	−0.0166

Frequencies

Table S46: CMA-0A residuals with respect to the reference harmonic frequencies (in cm^{-1}) for $(CH_4)_2$ using natural internal coordinates with cartesian force constants.

	Reference	CMA-0A										
	CCSD(T)	MP2						CCSD(T)				
	aTZ	DZ	haDZ	aDZ	TZ	haTZ	aTZ	DZ	haDZ	aDZ	TZ	haTZ
$\omega_1(a_{1g})$	3146.9	−0.11	−0.04	−0.03	−0.01	−0.01	−0.00	−0.08	−0.02	−0.02	−0.00	−0.00
$\omega_2(a_{1g})$	3026.7	0.00	0.00	0.00	0.00	0.00	−0.00	0.00	0.00	0.00	0.00	0.00
$\omega_3(a_{1g})$	1353.7	0.26	0.08	0.08	0.03	0.02	0.01	0.20	0.05	0.04	0.01	0.00
$\omega_4(a_{1g})$	68.3	0.01	0.03	0.02	0.01	0.01	0.01	0.01	0.03	0.02	0.01	0.01
$\omega_{5a}(e_g)$	3144.7	−0.10	−0.02	−0.02	−0.01	−0.00	−0.00	−0.07	−0.01	−0.01	−0.00	0.00
$\omega_{5b}(e_g)$	3144.7	−0.10	−0.02	−0.02	−0.01	−0.00	−0.00	−0.07	−0.01	−0.01	−0.00	0.00
$\omega_{6a}(e_g)$	1565.0	−0.00	−0.00	−0.03	0.00	−0.00	0.00	−0.00	−0.01	−0.03	0.00	0.00
$\omega_{6b}(e_g)$	1565.0	−0.00	−0.00	−0.03	0.00	−0.00	0.00	−0.00	−0.01	−0.03	0.00	0.00
$\omega_{7a}(e_g)$	1353.3	0.23	0.07	0.10	0.02	0.01	0.01	0.17	0.04	0.07	0.00	0.00
$\omega_{7b}(e_g)$	1353.3	0.23	0.07	0.10	0.02	0.01	0.01	0.17	0.04	0.07	0.00	0.00
$\omega_{8a}(e_g)$	64.3	0.08	0.08	0.08	0.09	0.09	0.09	0.08	0.08	0.08	0.09	0.09
$\omega_{8b}(e_g)$	64.3	0.08	0.08	0.08	0.09	0.09	0.09	0.08	0.08	0.08	0.09	0.09
$\omega_9(a_{1u})$	118.7	−0.00	−0.00	−0.00	−0.00	0.00	0.00	0.00	−0.00	0.00	−0.00	0.00
$\omega_{10}(a_{2u})$	3145.5	−0.11	−0.03	−0.03	−0.01	−0.01	−0.00	−0.08	−0.02	−0.02	−0.00	−0.00
$\omega_{11}(a_{2u})$	3027.1	0.00	0.00	−0.00	0.00	0.00	0.00	0.00	0.00	−0.00	0.00	0.00
$\omega_{12}(a_{2u})$	1344.3	0.25	0.07	0.07	0.03	0.01	0.01	0.19	0.04	0.04	0.01	0.00
$\omega_{13a}(e_u)$	3146.6	−0.09	−0.03	−0.02	−0.01	−0.01	−0.00	−0.06	−0.01	−0.01	−0.00	−0.00
$\omega_{13b}(e_u)$	3146.6	−0.09	−0.03	−0.02	−0.01	−0.01	−0.00	−0.06	−0.01	−0.01	−0.00	−0.00
$\omega_{14a}(e_u)$	1575.5	0.00	−0.00	−0.02	−0.00	−0.00	−0.00	0.00	−0.00	−0.02	−0.00	−0.00
$\omega_{14b}(e_u)$	1575.5	0.00	−0.00	−0.02	−0.00	−0.00	−0.00	0.00	−0.00	−0.02	−0.00	−0.00
$\omega_{15a}(e_u)$	1349.9	0.21	0.06	0.07	0.02	0.01	0.01	0.15	0.03	0.05	0.00	0.00
$\omega_{15b}(e_u)$	1349.9	0.21	0.06	0.07	0.02	0.01	0.01	0.15	0.03	0.05	0.00	0.00
$\omega_{16a}(e_u)$	101.1	0.00	0.00	0.00	0.01	0.01	0.01	0.00	0.00	0.00	0.01	0.01
$\omega_{16b}(e_u)$	101.1	0.00	0.00	0.00	0.01	0.01	0.01	0.00	0.00	0.00	0.01	0.01

Table S47: Pure level B residuals with respect to the reference harmonic frequencies (in cm^{-1}) for $(CH_4)_2$ using natural internal coordinates with cartesian force constants.

	Reference	Pure										
	CCSD(T)	MP2						CCSD(T)				
	aTZ	DZ	haDZ	aDZ	TZ	haTZ	aTZ	DZ	haDZ	aDZ	TZ	haTZ
$\omega_1(a_{1g})$	3146.9	174.80	147.75	132.87	23.60	23.73	23.63	155.98	129.18	112.62	−1.24	−0.47
$\omega_2(a_{1g})$	3026.7	137.50	116.83	104.38	10.20	10.02	10.06	131.99	111.47	96.78	−0.55	−0.32
$\omega_3(a_{1g})$	1353.7	4.15	9.76	−9.98	−9.71	−1.84	−1.70	7.08	14.13	−5.72	−8.64	−0.16
$\omega_4(a_{1g})$	68.3	7.42	6.88	4.98	3.03	1.10	0.23	6.92	7.40	5.06	2.66	0.90
$\omega_{5a}(e_g)$	3144.7	176.09	147.77	134.46	23.85	24.01	23.80	156.90	129.02	114.19	−1.28	−0.40
$\omega_{5b}(e_g)$	3144.7	176.09	147.77	134.46	23.85	24.01	23.80	156.90	129.02	114.19	−1.28	−0.40
$\omega_{6a}(e_g)$	1565.0	12.65	12.75	−3.00	2.14	7.46	6.57	7.69	8.60	−7.98	−5.33	1.05
$\omega_{6b}(e_g)$	1565.0	12.65	12.75	−3.00	2.14	7.46	6.57	7.69	8.60	−7.98	−5.33	1.05
$\omega_{7a}(e_g)$	1353.3	5.47	11.59	−10.09	−7.83	−0.93	−1.42	8.16	15.38	−6.23	−7.10	0.41
$\omega_{7b}(e_g)$	1353.3	5.47	11.59	−10.09	−7.83	−0.93	−1.42	8.16	15.38	−6.23	−7.10	0.41
$\omega_{8a}(e_g)$	64.3	15.37	22.64	30.11	0.07	17.04	5.13	10.48	19.23	27.87	−7.35	14.37
$\omega_{8b}(e_g)$	64.3	15.37	22.64	30.11	0.07	17.04	5.13	10.48	19.23	27.87	−7.35	14.37
$\omega_9(a_{1u})$	118.7	17.26	36.02	28.07	14.84	14.73	4.65	14.30	33.53	25.28	10.48	11.93
$\omega_{10}(a_{2u})$	3145.5	175.01	147.42	132.87	23.80	23.94	23.89	155.95	128.62	112.39	−1.32	−0.53
$\omega_{11}(a_{2u})$	3027.1	136.69	116.39	103.80	9.62	9.74	9.99	131.13	110.90	96.15	−1.27	−0.71
$\omega_{12}(a_{2u})$	1344.3	2.77	9.18	−12.54	−10.49	−2.64	−2.35	6.94	14.32	−7.38	−8.68	−0.31
$\omega_{13a}(e_u)$	3146.6	175.97	147.60	134.08	23.67	23.72	23.40	157.16	129.24	114.15	−0.99	−0.26
$\omega_{13b}(e_u)$	3146.6	175.97	147.60	134.08	23.67	23.72	23.40	157.16	129.24	114.15	−0.99	−0.26
$\omega_{14a}(e_u)$	1575.5	12.70	13.20	−2.04	4.08	8.37	7.15	6.74	8.29	−7.85	−3.90	1.40
$\omega_{14b}(e_u)$	1575.5	12.70	13.20	−2.04	4.08	8.37	7.15	6.74	8.29	−7.85	−3.90	1.40
$\omega_{15a}(e_u)$	1349.9	4.16	11.74	−11.24	−8.56	−1.84	−1.61	7.55	15.96	−6.90	−7.51	−0.18
$\omega_{15b}(e_u)$	1349.9	4.16	11.74	−11.24	−8.56	−1.84	−1.61	7.55	15.96	−6.90	−7.51	−0.18
$\omega_{16a}(e_u)$	101.1	18.63	34.56	33.37	9.00	17.98	5.01	14.76	31.63	30.83	2.95	15.08
$\omega_{16b}(e_u)$	101.1	18.63	34.56	33.37	9.00	17.98	5.01	14.76	31.63	30.83	2.95	15.08

Natural Internal Coordinates

Table S48: Symmetrized, unnormalized natural internal coordinates for methane dimer.

1	$r_{1,5} + r_{6,7}$
2	$r_{1,5} - r_{6,7}$
3	$r_{1,2} + r_{1,3} + r_{1,4} + r_{6,9} + r_{6,8} + r_{6,10}$
4	$2r_{1,2} - r_{1,3} - r_{1,4} + 2r_{6,9} - r_{6,8} - r_{6,10}$
5	$r_{1,3} - r_{1,4} + r_{6,8} - r_{6,10}$
6	$r_{1,2} + r_{1,3} + r_{1,4} - r_{6,9} - r_{6,8} - r_{6,10}$
7	$2r_{1,2} - r_{1,3} - r_{1,4} - 2r_{6,9} + r_{6,8} + r_{6,10}$
8	$r_{1,3} - r_{1,4} - r_{6,8} + r_{6,10}$
9	$r_{1,6}$
10	$\phi_{5,1,2} + \phi_{5,1,3} + \phi_{5,1,4} - \phi_{3,1,4} - \phi_{2,1,4} - \phi_{3,1,2} + \phi_{7,6,9} + \phi_{7,6,8} + \phi_{7,6,10} - \phi_{8,6,10}$ $-\phi_{9,6,10} - \phi_{8,6,9}$
11	$2\phi_{5,1,2} - \phi_{5,1,3} - \phi_{5,1,4} + 2\phi_{7,6,9} - \phi_{7,6,8} - \phi_{7,6,10}$
12	$\phi_{5,1,3} - \phi_{5,1,4} + \phi_{7,6,8} - \phi_{7,6,10}$
13	$2\phi_{3,1,4} - \phi_{2,1,4} - \phi_{3,1,2} + 2\phi_{8,6,10} - \phi_{9,6,10} - \phi_{8,6,9}$
14	$\phi_{2,1,4} - \phi_{3,1,2} + \phi_{9,6,10} - \phi_{8,6,9}$
15	$\phi_{5,1,2} + \phi_{5,1,3} + \phi_{5,1,4} - \phi_{3,1,4} - \phi_{2,1,4} - \phi_{3,1,2} - \phi_{7,6,9} - \phi_{7,6,8} - \phi_{7,6,10} + \phi_{8,6,10}$ $+\phi_{9,6,10} + \phi_{8,6,9}$
16	$2\phi_{5,1,2} - \phi_{5,1,3} - \phi_{5,1,4} - 2\phi_{7,6,9} + \phi_{7,6,8} + \phi_{7,6,10}$
17	$\phi_{5,1,3} - \phi_{5,1,4} - \phi_{7,6,8} + \phi_{7,6,10}$
18	$2\phi_{3,1,4} - \phi_{2,1,4} - \phi_{3,1,2} - 2\phi_{8,6,10} + \phi_{9,6,10} + \phi_{8,6,9}$
19	$\phi_{2,1,4} - \phi_{3,1,2} - \phi_{9,6,10} + \phi_{8,6,9}$
20	$\tau_{8,6,1,3} + \tau_{9,6,1,2} + \tau_{10,6,1,4}$
21	$2\alpha^x_{2,1,6,7} - \alpha^x_{3,1,6,7} - \alpha^x_{4,1,6,7} + 2\alpha^x_{9,6,1,5} - \alpha^x_{8,6,1,5} - \alpha^x_{10,6,1,5}$
22	$\alpha^x_{3,1,6,7} - \alpha^x_{4,1,6,7} + \alpha^x_{8,6,1,5} - \alpha^x_{10,6,1,5}$
23	$2\alpha^x_{2,1,6,7} - \alpha^x_{3,1,6,7} - \alpha^x_{4,1,6,7} - 2\alpha^x_{9,6,1,5} + \alpha^x_{8,6,1,5} + \alpha^x_{10,6,1,5}$
24	$\alpha^x_{3,1,6,7} - \alpha^x_{4,1,6,7} - \alpha^x_{8,6,1,5} + \alpha^x_{10,6,1,5}$

S3.13 benzene−methane dimer

Structure

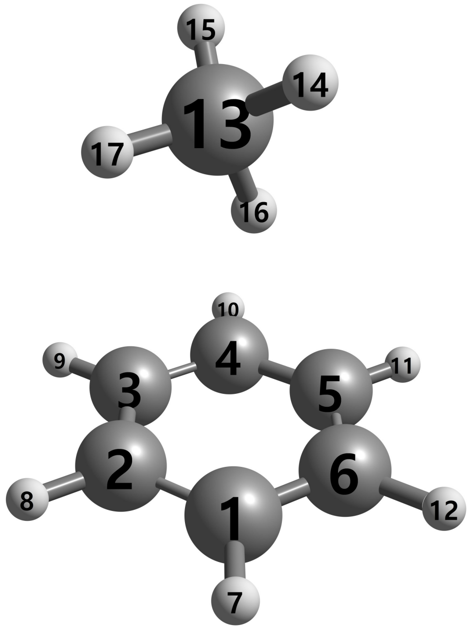

Figure S13: Benzene-methane dimer structure labeled with atomic indices. The plane of symmetry cuts vertically through C_2 and C_5, where $C_\sigma = C_2$.

Frequencies

Table S49: CMA-0A residuals with respect to the reference harmonic frequencies (in cm^{-1}) for benzene$\cdots CH_4$ using natural internal coordinates with cartesian force constants.

	Reference	CMA-0A	
	CCSD(T)	MP2	
	haTZ	TZ	haTZ
$\omega_1(a')$	3201.3	0.0	0.1
$\omega_2(a')$	3190.7	0.0	0.0
$\omega_3(a')$	3173.0	0.0	0.0
$\omega_4(a')$	3160.5	0.0	0.0
$\omega_5(a')$	3153.7	0.0	−0.1
$\omega_6(a')$	3141.5	0.0	0.0
$\omega_7(a')$	3025.0	0.0	0.0
$\omega_8(a')$	1629.7	0.0	0.0
$\omega_9(a')$	1571.1	0.0	0.0
$\omega_{10}(a')$	1502.8	0.0	0.0
$\omega_{11}(a')$	1350.1	0.0	0.0
$\omega_{12}(a')$	1343.8	0.0	0.0
$\omega_{13}(a')$	1185.8	0.0	0.1
$\omega_{14}(a')$	1051.4	0.0	0.0
$\omega_{15}(a')$	1011.1	−0.2	0.0
$\omega_{16}(a')$	1002.4	0.0	0.0
$\omega_{17}(a')$	978.0	−2.5	−0.1
$\omega_{18}(a')$	975.5	−0.1	0.0
$\omega_{19}(a')$	855.8	0.0	0.0
$\omega_{20}(a')$	683.8	3.8	0.2
$\omega_{21}(a')$	682.4	0.0	0.0
$\omega_{22}(a')$	606.4	0.0	0.0
$\omega_{23}(a')$	397.9	0.2	0.0
$\omega_{24}(a')$	89.7	0.0	−3.3
$\omega_{25}(a')$	63.7	0.1	0.1
$\omega_{26}(a')$	31.8	0.1	8.4
$\omega_{27}(a'')$	3190.9	0.0	0.0
$\omega_{28}(a'')$	3173.2	0.0	0.0
$\omega_{29}(a'')$	3139.9	0.0	0.0
$\omega_{30}(a'')$	1629.8	0.0	0.0
$\omega_{31}(a'')$	1572.2	0.0	0.0
$\omega_{32}(a'')$	1502.8	0.0	0.0
$\omega_{33}(a'')$	1368.9	0.0	0.0
$\omega_{34}(a'')$	1351.8	0.0	0.0
$\omega_{35}(a'')$	1331.1	−3.7	−4.0
$\omega_{36}(a'')$	1185.8	0.0	0.0
$\omega_{37}(a'')$	1155.0	4.2	4.6
$\omega_{38}(a'')$	1051.5	0.0	0.0
$\omega_{39}(a'')$	976.1	−0.1	0.0
$\omega_{40}(a'')$	856.1	0.0	0.0
$\omega_{41}(a'')$	606.4	0.0	0.0
$\omega_{42}(a'')$	397.6	0.2	0.0
$\omega_{43}(a'')$	76.0	−0.5	−0.5
$\omega_{44}(a'')$	18.7	−1.1	−0.3
$\omega_{45}(a'')$	13.0i	6.4	4.0

Table S50: Pure level B residuals with respect to the reference harmonic frequencies (in cm^{-1}) for benzene$\cdots CH_4$ using natural internal coordinates with cartesian force constants.

	Reference	Pure	
	CCSD(T)	MP2	
	haTZ	TZ	haTZ
$\omega_1(a')$	3201.3	14.37	15.13
$\omega_2(a')$	3190.7	15.26	16.04
$\omega_3(a')$	3173.0	16.84	17.13
$\omega_4(a')$	3160.5	18.72	16.90
$\omega_5(a')$	3153.7	20.18	19.23
$\omega_6(a')$	3141.5	24.87	24.91
$\omega_7(a')$	3025.0	10.19	9.67
$\omega_8(a')$	1629.7	−7.83	−12.98
$\omega_9(a')$	1571.1	1.69	4.57
$\omega_{10}(a')$	1502.8	−7.59	−9.70
$\omega_{11}(a')$	1350.1	−10.42	−4.55
$\omega_{12}(a')$	1343.8	−10.34	−3.10
$\omega_{13}(a')$	1185.8	2.56	−0.78
$\omega_{14}(a')$	1051.4	0.50	−1.25
$\omega_{15}(a')$	1011.1	3.40	1.43
$\omega_{16}(a')$	1002.4	−0.36	−1.58
$\omega_{17}(a')$	978.0	−7.29	3.30
$\omega_{18}(a')$	975.5	−18.94	0.04
$\omega_{19}(a')$	855.8	2.35	2.53
$\omega_{20}(a')$	683.8	3.75	5.56
$\omega_{21}(a')$	682.4	−0.74	3.19
$\omega_{22}(a')$	606.4	−3.68	−4.71
$\omega_{23}(a')$	397.9	2.77	0.58
$\omega_{24}(a')$	89.7	−8.07	−13.29
$\omega_{25}(a')$	63.7	0.03	−6.56
$\omega_{26}(a')$	31.8	−9.33	−67.84
$\omega_{27}(a'')$	3190.9	15.24	16.02
$\omega_{28}(a'')$	3173.2	16.74	17.08
$\omega_{29}(a'')$	3139.9	24.93	25.10
$\omega_{30}(a'')$	1629.8	−7.68	−12.85
$\omega_{31}(a'')$	1572.2	2.56	5.82
$\omega_{32}(a'')$	1502.8	−7.23	−9.42
$\omega_{33}(a'')$	1368.9	71.97	76.92
$\omega_{34}(a'')$	1351.8	10.68	10.74
$\omega_{35}(a'')$	1331.1	11.83	17.76
$\omega_{36}(a'')$	1185.8	3.01	−0.36
$\omega_{37}(a'')$	1155.0	7.65	4.39
$\omega_{38}(a'')$	1051.5	0.58	−1.15
$\omega_{39}(a'')$	976.1	−20.11	−0.01
$\omega_{40}(a'')$	856.1	1.93	2.43
$\omega_{41}(a'')$	606.4	−3.50	−4.51
$\omega_{42}(a'')$	397.6	3.03	0.79
$\omega_{43}(a'')$	76.0	−5.74	−6.49
$\omega_{44}(a'')$	18.7	5.98	5.71
$\omega_{45}(a'')$	13.0i	−20.32	−21.94

Natural Internal Coordinates

Table S51: Symmetrized, unnormalized natural internal coordinates for benzene−methane dimer.

1	$r_{3,4} + r_{4,5} + r_{5,6} + r_{6,1} + r_{1,2} + r_{2,3}$
2	$r_{3,4} - r_{4,5} + r_{5,6} - r_{6,1} + r_{1,2} - r_{2,3}$
3	$2r_{3,4} + r_{4,5} - r_{5,6} - 2r_{6,1} - r_{1,2} + r_{2,3}$
4	$r_{4,5} + r_{5,6} - r_{1,2} - r_{2,3}$
5	$2r_{3,4} - r_{4,5} - r_{5,6} + 2r_{6,1} - r_{1,2} - r_{2,3}$
6	$r_{4,5} - r_{5,6} + r_{1,2} - r_{2,3}$
7	$r_{2,8} + r_{3,9} + r_{4,10} + r_{5,11} + r_{6,12} + r_{1,7}$
8	$r_{2,8} - r_{3,9} + r_{4,10} - r_{5,11} + r_{6,12} - r_{1,7}$
9	$2r_{2,8} + r_{3,9} - r_{4,10} - 2r_{5,11} - r_{6,12} + r_{1,7}$
10	$r_{3,9} + r_{4,10} - r_{6,12} - r_{1,7}$
11	$2r_{2,8} - r_{3,9} - r_{4,10} + 2r_{5,11} - r_{6,12} - r_{1,7}$
12	$r_{3,9} - r_{4,10} + r_{6,12} - r_{1,7}$
13	$r_{13,16}$
14	$r_{13,17} + r_{13,15} + r_{13,14}$
15	$2r_{13,17} - r_{13,15} - r_{13,14}$
16	$r_{13,15} - r_{13,14}$
17	$r_{16,2} + r_{16,4} + r_{16,6}$
18	$2r_{16,2} - r_{16,4} - r_{16,6}$
19	$r_{16,4} - r_{16,6}$
20	$\phi_{1,2,3} - \phi_{2,3,4} + \phi_{3,4,5} - \phi_{4,5,6} + \phi_{5,6,1} - \phi_{6,1,2}$
21	$2\phi_{1,2,3} - \phi_{2,3,4} - \phi_{3,4,5} + 2\phi_{4,5,6} - \phi_{5,6,1} - \phi_{6,1,2}$
22	$\phi_{2,3,4} - \phi_{3,4,5} + \phi_{5,6,1} - \phi_{6,1,2}$
23	$\phi_{8,2,3} - \phi_{8,2,1} + \phi_{9,3,4} - \phi_{9,3,2} + \phi_{10,4,5} - \phi_{10,4,3} + \phi_{11,5,6} - \phi_{11,5,4} + \phi_{12,6,1} - \phi_{12,6,5}$ $+\phi_{7,1,2} - \phi_{7,1,6}$
24	$\phi_{8,2,3} - \phi_{8,2,1} - \phi_{9,3,4} + \phi_{9,3,2} + \phi_{10,4,5} - \phi_{10,4,3} - \phi_{11,5,6} + \phi_{11,5,4} + \phi_{12,6,1} - \phi_{12,6,5}$ $-\phi_{7,1,2} + \phi_{7,1,6}$
25	$2\phi_{8,2,3} - 2\phi_{8,2,1} + \phi_{9,3,4} - \phi_{9,3,2} - \phi_{10,4,5} + \phi_{10,4,3} - 2\phi_{11,5,6} + 2\phi_{11,5,4} - \phi_{12,6,1} + \phi_{12,6,5}$ $+\phi_{7,1,2} - \phi_{7,1,6}$
26	$\phi_{9,3,4} - \phi_{9,3,2} + \phi_{10,4,5} - \phi_{10,4,3} - \phi_{12,6,1} + \phi_{12,6,5} - \phi_{7,1,2} + \phi_{7,1,6}$
27	$2\phi_{8,2,3} - 2\phi_{8,2,1} - \phi_{9,3,4} + \phi_{9,3,2} - \phi_{10,4,5} + \phi_{10,4,3} + 2\phi_{11,5,6} - 2\phi_{11,5,4} - \phi_{12,6,1} + \phi_{12,6,5}$ $-\phi_{7,1,2} + \phi_{7,1,6}$
28	$\phi_{9,3,4} - \phi_{9,3,2} - \phi_{10,4,5} + \phi_{10,4,3} + \phi_{12,6,1} - \phi_{12,6,5} - \phi_{7,1,2} + \phi_{7,1,6}$
29	$\phi_{16,13,17} + \phi_{16,13,15} + \phi_{16,13,14} - \phi_{14,13,15} - \phi_{14,13,17} - \phi_{17,13,15}$
30	$2\phi_{16,13,17} - \phi_{16,13,15} - \phi_{16,13,14}$
31	$\phi_{16,13,15} - \phi_{16,13,14}$
32	$2\phi_{14,13,15} - \phi_{14,13,17} - \phi_{17,13,15}$
33	$\phi_{14,13,17} - \phi_{17,13,15}$
34	$2\phi_{13,16,2} - \phi_{13,16,4} - \phi_{13,16,6}$
35	$\phi_{13,16,4} - \phi_{13,16,6}$
36	$\tau_{1,2,3,4} - \tau_{2,3,4,5} + \tau_{3,4,5,6} - \tau_{4,5,6,1} + \tau_{5,6,1,2} - \tau_{6,1,2,3}$
37	$\tau_{1,2,3,4} - \tau_{3,4,5,6} + \tau_{4,5,6,1} - \tau_{6,1,2,3}$
38	$-\tau_{1,2,3,4} + 2\tau_{2,3,4,5} - \tau_{3,4,5,6} - \tau_{4,5,6,1} + 2\tau_{5,6,1,2} - \tau_{6,1,2,3}$
39	$\tau_{14,13,16,2} + \tau_{15,13,16,2} + \tau_{17,13,16,2}$
40	$\gamma_{8,2,3,1} + \gamma_{9,3,4,2} + \gamma_{10,4,5,3} + \gamma_{11,5,6,4} + \gamma_{12,6,1,5} + \gamma_{7,1,2,6}$
41	$\gamma_{8,2,3,1} - \gamma_{9,3,4,2} + \gamma_{10,4,5,3} - \gamma_{11,5,6,4} + \gamma_{12,6,1,5} - \gamma_{7,1,2,6}$
42	$2\gamma_{8,2,3,1} + \gamma_{9,3,4,2} - \gamma_{10,4,5,3} - 2\gamma_{11,5,6,4} - \gamma_{12,6,1,5} + \gamma_{7,1,2,6}$
43	$\gamma_{9,3,4,2} + \gamma_{10,4,5,3} - \gamma_{12,6,1,5} - \gamma_{7,1,2,6}$
44	$2\gamma_{8,2,3,1} - \gamma_{9,3,4,2} - \gamma_{10,4,5,3} + 2\gamma_{11,5,6,4} - \gamma_{12,6,1,5} - \gamma_{7,1,2,6}$
45	$\gamma_{9,3,4,2} - \gamma_{10,4,5,3} + \gamma_{12,6,1,5} - \gamma_{7,1,2,6}$

S3.14 benzene−hydrogen cyanide dimer

Structure

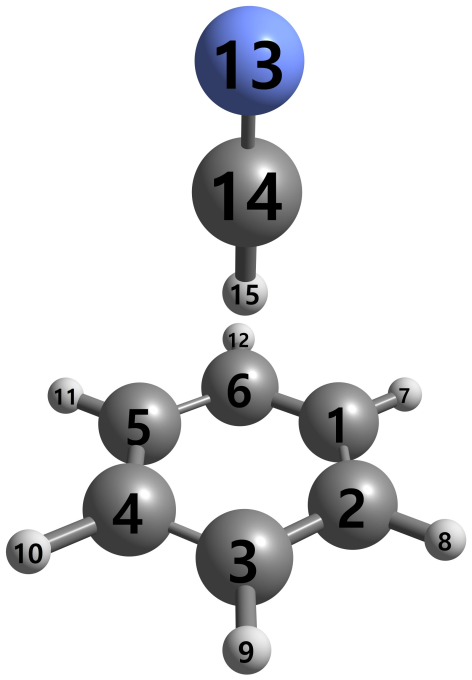

Figure S14: Benzene-hydrogen cyanide dimer structure labeled with atomic indices.

Frequencies

Table S52: CMA-0A residuals with respect to the reference harmonic frequencies (in cm^{-1}) for benzene$\cdots$HCN using natural internal coordinates with cartesian force constants.

	Reference	CMA-0A	
	CCSD(T)	MP2	
	haTZ	TZ	haTZ
$\omega_1(a_1)$	3392.1	0.0	−0.1
$\omega_2(a_1)$	3205.1	0.0	0.0
$\omega_3(a_1)$	2105.8	0.1	0.1
$\omega_4(a_1)$	1000.9	0.0	0.0
$\omega_5(a_1)$	692.8	0.0	0.0
$\omega_6(a_1)$	108.1	0.0	0.0
$\omega_7(a_2)$	1369.6	0.0	0.0
$\omega_8(b_1)$	1330.4	−3.6	−3.9
$\omega_9(b_1)$	1157.4	4.1	4.5
$\omega_{10}(b_2)$	3166.7	0.0	0.0
$\omega_{11}(b_2)$	1013.3	−0.4	0.0
$\omega_{12}(b_2)$	986.4	−1.3	−0.1
$\omega_{13}(b_2)$	686.2	2.5	0.2
$\omega_{14a}(e_1)$	3195.5	0.0	0.0
$\omega_{14b}(e_1)$	3195.5	0.0	0.0
$\omega_{15a}(e_1)$	1502.3	0.0	0.0
$\omega_{15b}(e_1)$	1502.3	0.0	0.0
$\omega_{16a}(e_1)$	1051.2	0.0	0.0
$\omega_{16b}(e_1)$	1051.2	0.0	0.0
$\omega_{17a}(e_1)$	865.2	0.0	0.0
$\omega_{17b}(e_1)$	865.2	0.0	0.0
$\omega_{18a}(e_1)$	721.7	0.0	−0.1
$\omega_{18b}(e_1)$	721.7	0.0	−0.1
$\omega_{19a}(e_1)$	111.5	−0.2	−0.3
$\omega_{19b}(e_1)$	111.5	−0.2	−0.3
$\omega_{20a}(e_1)$	14.6	1.7	4.2
$\omega_{20b}(e_1)$	14.6	1.7	4.2
$\omega_{21a}(e_2)$	3178.8	0.0	0.0
$\omega_{21b}(e_2)$	3178.8	0.0	0.0
$\omega_{22a}(e_2)$	1627.0	0.0	0.0
$\omega_{22b}(e_2)$	1627.0	0.0	0.0
$\omega_{23a}(e_2)$	1187.1	0.0	0.0
$\omega_{23b}(e_2)$	1187.1	0.0	0.0
$\omega_{24a}(e_2)$	982.7	0.0	0.0
$\omega_{24b}(e_2)$	982.7	0.0	0.0
$\omega_{25a}(e_2)$	606.8	0.0	0.0
$\omega_{25b}(e_2)$	606.8	0.0	0.0
$\omega_{26a}(e_2)$	403.5	0.1	0.0
$\omega_{26b}(e_2)$	403.5	0.1	0.0

Table S53: Pure level B residuals with respect to the reference harmonic frequencies (in cm^{-1}) for benzene$\cdots$HCN using natural internal coordinates with cartesian force constants.

	Reference	Pure	
	CCSD(T)	MP2	
	haTZ	TZ	haTZ
$\omega_1(a_1)$	3392.1	8.63	5.40
$\omega_2(a_1)$	3205.1	14.16	14.78
$\omega_3(a_1)$	2105.8	−34.85	−38.06
$\omega_4(a_1)$	1000.9	−0.40	−1.59
$\omega_5(a_1)$	692.8	4.67	3.19
$\omega_6(a_1)$	108.1	−6.44	−7.04
$\omega_7(a_2)$	1369.6	−6.34	−6.24
$\omega_8(b_1)$	1330.4	107.51	112.08
$\omega_9(b_1)$	1157.4	7.55	4.31
$\omega_{10}(b_2)$	3166.7	18.73	16.57
$\omega_{11}(b_2)$	1013.3	3.29	0.30
$\omega_{12}(b_2)$	986.4	−7.32	3.73
$\omega_{13}(b_2)$	686.2	1.71	5.27
$\omega_{14a}(e_1)$	3195.5	14.98	15.67
$\omega_{14b}(e_1)$	3195.5	14.98	15.67
$\omega_{15a}(e_1)$	1502.3	−7.46	−9.45
$\omega_{15b}(e_1)$	1502.3	−7.46	−9.45
$\omega_{16a}(e_1)$	1051.2	0.47	−1.16
$\omega_{16b}(e_1)$	1051.2	0.47	−1.16
$\omega_{17a}(e_1)$	865.2	2.14	2.24
$\omega_{17b}(e_1)$	865.2	2.14	2.24
$\omega_{18a}(e_1)$	721.7	4.41	−6.43
$\omega_{18b}(e_1)$	721.7	4.41	−6.43
$\omega_{19a}(e_1)$	111.5	−7.91	−8.48
$\omega_{19b}(e_1)$	111.5	−7.91	−8.48
$\omega_{20a}(e_1)$	14.6	−1.03	−45.53
$\omega_{20b}(e_1)$	14.6	−1.03	−45.53
$\omega_{21a}(e_2)$	3178.8	16.47	16.74
$\omega_{21b}(e_2)$	3178.8	16.47	16.74
$\omega_{22a}(e_2)$	1627.0	−7.73	−12.84
$\omega_{22b}(e_2)$	1627.0	−7.73	−12.84
$\omega_{23a}(e_2)$	1187.1	2.97	−0.47
$\omega_{23b}(e_2)$	1187.1	2.97	−0.47
$\omega_{24a}(e_2)$	982.7	−15.08	−0.09
$\omega_{24b}(e_2)$	982.7	−15.08	−0.09
$\omega_{25a}(e_2)$	606.8	−3.37	−4.67
$\omega_{25b}(e_2)$	606.8	−3.37	−4.67
$\omega_{26a}(e_2)$	403.5	2.62	−0.04
$\omega_{26b}(e_2)$	403.5	2.62	−0.04

Natural Internal Coordinates

Table S54: Symmetrized, unnormalized natural internal coordinates for benzene−hcn dimer.

1	$r_{1,2} + r_{2,3} + r_{3,4} + r_{4,5} + r_{5,6} + r_{6,1}$
2	$r_{1,2} - r_{2,3} + r_{3,4} - r_{4,5} + r_{5,6} - r_{6,1}$
3	$2r_{1,2} + r_{2,3} - r_{3,4} - 2r_{4,5} - r_{5,6} + r_{6,1}$
4	$r_{2,3} + r_{3,4} - r_{5,6} - r_{6,1}$
5	$2r_{1,2} - r_{2,3} - r_{3,4} + 2r_{4,5} - r_{5,6} - r_{6,1}$
6	$r_{2,3} - r_{3,4} + r_{5,6} - r_{6,1}$
7	$r_{3,9} + r_{4,10} + r_{5,11} + r_{6,12} + r_{1,7} + r_{2,8}$
8	$r_{3,9} - r_{4,10} + r_{5,11} - r_{6,12} + r_{1,7} - r_{2,8}$
9	$-2r_{3,9} - r_{4,10} + r_{5,11} + 2r_{6,12} + r_{1,7} - r_{2,8}$
10	$r_{4,10} + r_{5,11} - r_{1,7} - r_{2,8}$
11	$2r_{3,9} - r_{4,10} - r_{5,11} + 2r_{6,12} - r_{1,7} - r_{2,8}$
12	$r_{4,10} - r_{5,11} + r_{1,7} - r_{2,8}$
13	$r_{13,14}$
14	$r_{14,15}$
15	$r_{15,6} + r_{15,2} + r_{15,4}$
16	$-2r_{15,6} + r_{15,2} + r_{15,4}$
17	$r_{15,2} - r_{15,4}$
18	$\phi_{5,6,1} - \phi_{6,1,2} + \phi_{1,2,3} - \phi_{2,3,4} + \phi_{3,4,5} - \phi_{4,5,6}$
19	$2\phi_{5,6,1} - \phi_{6,1,2} - \phi_{1,2,3} + 2\phi_{2,3,4} - \phi_{3,4,5} - \phi_{4,5,6}$
20	$\phi_{6,1,2} - \phi_{1,2,3} + \phi_{3,4,5} - \phi_{4,5,6}$
21	$\phi_{12,6,1} - \phi_{12,6,5} + \phi_{7,1,2} - \phi_{7,1,6} + \phi_{8,2,3} - \phi_{8,2,1} + \phi_{9,3,4} - \phi_{9,3,2} + \phi_{10,4,5} - \phi_{10,4,3} + \phi_{11,5,6} - \phi_{11,5,4}$
22	$\phi_{12,6,1} - \phi_{12,6,5} - \phi_{7,1,2} + \phi_{7,1,6} + \phi_{8,2,3} - \phi_{8,2,1} - \phi_{9,3,4} + \phi_{9,3,2} + \phi_{10,4,5} - \phi_{10,4,3} - \phi_{11,5,6} + \phi_{11,5,4}$
23	$2\phi_{12,6,1} - 2\phi_{12,6,5} + \phi_{7,1,2} - \phi_{7,1,6} - \phi_{8,2,3} + \phi_{8,2,1} - 2\phi_{9,3,4} + 2\phi_{9,3,2} - \phi_{10,4,5} + \phi_{10,4,3} + \phi_{11,5,6} - \phi_{11,5,4}$
24	$\phi_{7,1,2} - \phi_{7,1,6} + \phi_{8,2,3} - \phi_{8,2,1} - \phi_{10,4,5} + \phi_{10,4,3} - \phi_{11,5,6} + \phi_{11,5,4}$
25	$-2\phi_{12,6,1} + 2\phi_{12,6,5} + \phi_{7,1,2} - \phi_{7,1,6} + \phi_{8,2,3} - \phi_{8,2,1} - 2\phi_{9,3,4} + 2\phi_{9,3,2} + \phi_{10,4,5} - \phi_{10,4,3} + \phi_{11,5,6} - \phi_{11,5,4}$
26	$\phi_{7,1,2} - \phi_{7,1,6} - \phi_{8,2,3} + \phi_{8,2,1} + \phi_{10,4,5} - \phi_{10,4,3} - \phi_{11,5,6} + \phi_{11,5,4}$
27	$-2\phi_{14,15,6} + \phi_{14,15,2} + \phi_{14,15,4}$
28	$\phi_{14,15,2} - \phi_{14,15,4}$
29	$\tau_{3,4,5,6} - \tau_{4,5,6,1} + \tau_{5,6,1,2} - \tau_{6,1,2,3} + \tau_{1,2,3,4} - \tau_{2,3,4,5}$
30	$\tau_{4,5,6,1} - \tau_{5,6,1,2} + \tau_{1,2,3,4} - \tau_{2,3,4,5}$
31	$-2\tau_{3,4,5,6} + \tau_{4,5,6,1} + \tau_{5,6,1,2} - 2\tau_{6,1,2,3} + \tau_{1,2,3,4} + \tau_{2,3,4,5}$
32	$\gamma_{12,6,1,5} + \gamma_{7,1,2,6} + \gamma_{8,2,3,1} + \gamma_{9,3,4,2} + \gamma_{10,4,5,3} + \gamma_{11,5,6,4}$
33	$\gamma_{12,6,1,5} - \gamma_{7,1,2,6} + \gamma_{8,2,3,1} - \gamma_{9,3,4,2} + \gamma_{10,4,5,3} - \gamma_{11,5,6,4}$
34	$-2\gamma_{12,6,1,5} - \gamma_{7,1,2,6} + \gamma_{8,2,3,1} + 2\gamma_{9,3,4,2} + \gamma_{10,4,5,3} - \gamma_{11,5,6,4}$
35	$\gamma_{7,1,2,6} + \gamma_{8,2,3,1} - \gamma_{10,4,5,3} - \gamma_{11,5,6,4}$
36	$2\gamma_{12,6,1,5} - \gamma_{7,1,2,6} - \gamma_{8,2,3,1} + 2\gamma_{9,3,4,2} - \gamma_{10,4,5,3} - \gamma_{11,5,6,4}$
37	$\gamma_{7,1,2,6} - \gamma_{8,2,3,1} + \gamma_{10,4,5,3} - \gamma_{11,5,6,4}$
38	$\alpha^{x}_{6,15,14,13}$
39	$\alpha^{y}_{6,15,14,13}$

S3.15 benzene–water dimer

Structure

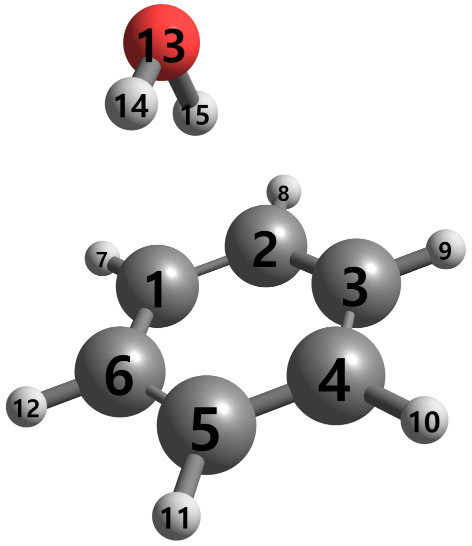

Figure S15: Benzene-water dimer structure labeled with atomic indices. The pseudo plane of symmetry cuts vertically through C_2 and C_5, where $C_\sigma = C_2$.

Frequencies

Table S55: CMA-0A residuals with respect to the reference harmonic frequencies (in cm^{-1}) for benzene$\cdots H_2O$ using natural internal coordinates with cartesian force constants.

	Reference	CMA-0A	
	CCSD(T)	MP2	
	haTZ	TZ	haTZ
$\omega_1(a)$	3899.0	0.1	0.1
$\omega_2(a)$	3789.4	−0.1	0.0
$\omega_3(a)$	3204.0	0.0	0.0
$\omega_4(a)$	3194.8	0.1	0.0
$\omega_5(a)$	3193.0	0.0	0.0
$\omega_6(a)$	3177.5	0.0	0.1
$\omega_7(a)$	3175.8	−0.1	0.0
$\omega_8(a)$	3163.9	0.0	−0.1
$\omega_9(a)$	1652.2	0.2	0.1
$\omega_{10}(a)$	1628.5	0.0	0.0
$\omega_{11}(a)$	1627.5	0.0	0.0
$\omega_{12}(a)$	1502.4	0.0	0.0
$\omega_{13}(a)$	1502.0	0.0	0.0
$\omega_{14}(a)$	1369.5	0.0	0.0
$\omega_{15}(a)$	1328.8	−3.8	−4.1
$\omega_{16}(a)$	1187.1	0.0	0.0
$\omega_{17}(a)$	1186.4	0.0	0.1
$\omega_{18}(a)$	1156.3	4.3	4.7
$\omega_{19}(a)$	1051.3	0.0	0.0
$\omega_{20}(a)$	1050.7	0.0	0.0
$\omega_{21}(a)$	1010.7	0.0	0.0
$\omega_{22}(a)$	1001.3	0.0	0.0
$\omega_{23}(a)$	979.2	−1.1	−0.2
$\omega_{24}(a)$	978.4	−1.0	0.0
$\omega_{25}(a)$	975.9	−0.1	0.0
$\omega_{26}(a)$	861.2	0.0	0.0
$\omega_{27}(a)$	859.4	0.0	0.0
$\omega_{28}(a)$	688.1	0.0	0.0
$\omega_{29}(a)$	677.6	3.0	0.2
$\omega_{30}(a)$	606.3	0.0	0.0
$\omega_{31}(a)$	606.0	0.0	0.0
$\omega_{32}(a)$	398.9	0.1	0.0
$\omega_{33}(a)$	396.1	0.1	0.0
$\omega_{34}(a)$	225.9	0.3	0.3
$\omega_{35}(a)$	129.6	−0.6	0.5
$\omega_{36}(a)$	96.4	0.8	0.3
$\omega_{37}(a)$	50.6	0.0	0.1
$\omega_{38}(a)$	43.3	1.9	0.2
$\omega_{39}(a)$	20.3	2.0	0.6

Table S56: Pure level B residuals with respect to the reference harmonic frequencies (in cm^{-1}) for benzene$\cdots H_2O$ using natural internal coordinates with cartesian force constants.

	Reference	Pure	
	CCSD(T)	MP2	
	haTZ	TZ	haTZ
$\omega_{1}(a)$	3899.0	20.47	21.95
$\omega_{2}(a)$	3789.4	5.12	2.61
$\omega_{3}(a)$	3204.0	14.03	14.64
$\omega_{4}(a)$	3194.8	14.94	15.57
$\omega_{5}(a)$	3193.0	14.59	15.53
$\omega_{6}(a)$	3177.5	15.51	16.63
$\omega_{7}(a)$	3175.8	16.08	16.55
$\omega_{8}(a)$	3163.9	17.23	16.40
$\omega_{9}(a)$	1652.2	−10.60	−19.67
$\omega_{10}(a)$	1628.5	−8.16	−13.05
$\omega_{11}(a)$	1627.5	−8.02	−12.85
$\omega_{12}(a)$	1502.4	−7.42	−9.35
$\omega_{13}(a)$	1502.0	−8.25	−9.37
$\omega_{14}(a)$	1369.5	68.34	73.29
$\omega_{15}(a)$	1328.8	33.62	34.51
$\omega_{16}(a)$	1187.1	2.55	−0.45
$\omega_{17}(a)$	1186.4	2.80	−0.32
$\omega_{18}(a)$	1156.3	7.62	4.76
$\omega_{19}(a)$	1051.3	0.61	−1.06
$\omega_{20}(a)$	1050.7	0.41	−1.26
$\omega_{21}(a)$	1010.7	1.77	0.92
$\omega_{22}(a)$	1001.3	−0.55	−1.66
$\omega_{23}(a)$	979.2	−4.63	3.18
$\omega_{24}(a)$	978.4	−16.71	−0.09
$\omega_{25}(a)$	975.9	−14.59	0.29
$\omega_{26}(a)$	861.2	2.52	2.33
$\omega_{27}(a)$	859.4	2.87	2.53
$\omega_{28}(a)$	688.1	4.75	2.75
$\omega_{29}(a)$	677.6	4.42	5.56
$\omega_{30}(a)$	606.3	−4.28	−4.47
$\omega_{31}(a)$	606.0	−4.54	−4.50
$\omega_{32}(a)$	398.9	3.27	0.82
$\omega_{33}(a)$	396.1	3.08	0.86
$\omega_{34}(a)$	225.9	−38.18	16.28
$\omega_{35}(a)$	129.6	21.63	1.97
$\omega_{36}(a)$	96.4	−4.06	−3.78
$\omega_{37}(a)$	50.6	−6.81	1.17
$\omega_{38}(a)$	43.3	−4.31	−3.84
$\omega_{39}(a)$	20.3	−43.88	−36.45

Natural Internal Coordinates

Table S57: Symmetrized, unnormalized natural internal coordinates for benzene−water dimer.

1	$r_{6,1} + r_{1,2} + r_{2,3} + r_{3,4} + r_{4,5} + r_{5,6}$
2	$r_{6,1} - r_{1,2} + r_{2,3} - r_{3,4} + r_{4,5} - r_{5,6}$
3	$2r_{6,1} + r_{1,2} - r_{2,3} - 2r_{3,4} - r_{4,5} + r_{5,6}$
4	$r_{1,2} + r_{2,3} - r_{4,5} - r_{5,6}$
5	$2r_{6,1} - r_{1,2} - r_{2,3} + 2r_{3,4} - r_{4,5} - r_{5,6}$
6	$r_{1,2} - r_{2,3} + r_{4,5} - r_{5,6}$
7	$r_{2,8} + r_{3,9} + r_{4,10} + r_{5,11} + r_{6,12} + r_{1,7}$
8	$r_{2,8} - r_{3,9} + r_{4,10} - r_{5,11} + r_{6,12} - r_{1,7}$
9	$2r_{2,8} + r_{3,9} - r_{4,10} - 2r_{5,11} - r_{6,12} + r_{1,7}$
10	$r_{3,9} + r_{4,10} - r_{6,12} - r_{1,7}$
11	$2r_{2,8} - r_{3,9} - r_{4,10} + 2r_{5,11} - r_{6,12} - r_{1,7}$
12	$r_{3,9} - r_{4,10} + r_{6,12} - r_{1,7}$
13	$r_{13,14} + r_{13,15}$
14	$r_{13,14} - r_{13,15}$
15	$r(13\|1,2,3,4,5,6)$
16	$\phi_{1,2,3} - \phi_{2,3,4} + \phi_{3,4,5} - \phi_{4,5,6} + \phi_{5,6,1} - \phi_{6,1,2}$
17	$2\phi_{1,2,3} - \phi_{2,3,4} - \phi_{3,4,5} + 2\phi_{4,5,6} - \phi_{5,6,1} - \phi_{6,1,2}$
18	$\phi_{2,3,4} - \phi_{3,4,5} + \phi_{5,6,1} - \phi_{6,1,2}$
19	$\phi_{8,2,3} - \phi_{8,2,1} + \phi_{9,3,4} - \phi_{9,3,2} + \phi_{10,4,5} - \phi_{10,4,3} + \phi_{11,5,6} - \phi_{11,5,4} + \phi_{12,6,1} - \phi_{12,6,5} + \phi_{7,1,2} - \phi_{7,1,6}$
20	$\phi_{8,2,3} - \phi_{8,2,1} - \phi_{9,3,4} + \phi_{9,3,2} + \phi_{10,4,5} - \phi_{10,4,3} - \phi_{11,5,6} + \phi_{11,5,4} + \phi_{12,6,1} - \phi_{12,6,5} - \phi_{7,1,2} + \phi_{7,1,6}$
21	$2\phi_{8,2,3} - 2\phi_{8,2,1} + \phi_{9,3,4} - \phi_{9,3,2} - \phi_{10,4,5} + \phi_{10,4,3} - 2\phi_{11,5,6} + 2\phi_{11,5,4} - \phi_{12,6,1} + \phi_{12,6,5} + \phi_{7,1,2} - \phi_{7,1,6}$
22	$\phi_{9,3,4} - \phi_{9,3,2} + \phi_{10,4,5} - \phi_{10,4,3} - \phi_{12,6,1} + \phi_{12,6,5} - \phi_{7,1,2} + \phi_{7,1,6}$
23	$2\phi_{8,2,3} - 2\phi_{8,2,1} - \phi_{9,3,4} + \phi_{9,3,2} - \phi_{10,4,5} + \phi_{10,4,3} + 2\phi_{11,5,6} - 2\phi_{11,5,4} - \phi_{12,6,1} + \phi_{12,6,5} - \phi_{7,1,2} + \phi_{7,1,6}$
24	$\phi_{9,3,4} - \phi_{9,3,2} - \phi_{10,4,5} + \phi_{10,4,3} + \phi_{12,6,1} - \phi_{12,6,5} - \phi_{7,1,2} + \phi_{7,1,6}$
25	$\phi_{14,13,15}$
26	$\phi(14,15\|13\|1,2,3,4,5,6)$
27	$\phi(13\|1,2,3,4,5,6\|1,2,3) - \phi(13\|1,2,3,4,5,6\|4,5,6)$
28	$\phi(13\|1,2,3,4,5,6\|3,4) - \phi(13\|1,2,3,4,5,6\|1,6)$
29	$\tau_{1,2,3,4} - \tau_{2,3,4,5} + \tau_{3,4,5,6} - \tau_{4,5,6,1} + \tau_{5,6,1,2} - \tau_{6,1,2,3}$
30	$\tau_{1,2,3,4} - \tau_{3,4,5,6} + \tau_{4,5,6,1} - \tau_{6,1,2,3}$
31	$-\tau_{1,2,3,4} + 2\tau_{2,3,4,5} - \tau_{3,4,5,6} - \tau_{4,5,6,1} + 2\tau_{5,6,1,2} - \tau_{6,1,2,3}$
32	$\tau(14,15\|13\|1,2,3,4,5,6\|1,2,3) + \tau(14,15\|13\|1,2,3,4,5,6\|4,5,6)$
33	$\tau(14\|14,15\|13\|1,2,3,4,5,6) + \tau(15\|14,15\|13\|1,2,3,4,5,6)$
34	$\gamma_{8,2,3,1} + \gamma_{9,3,4,2} + \gamma_{10,4,5,3} + \gamma_{11,5,6,4} + \gamma_{12,6,1,5} + \gamma_{7,1,2,6}$
35	$\gamma_{8,2,3,1} - \gamma_{9,3,4,2} + \gamma_{10,4,5,3} - \gamma_{11,5,6,4} + \gamma_{12,6,1,5} - \gamma_{7,1,2,6}$
36	$2\gamma_{8,2,3,1} + \gamma_{9,3,4,2} - \gamma_{10,4,5,3} - 2\gamma_{11,5,6,4} - \gamma_{12,6,1,5} + \gamma_{7,1,2,6}$
37	$\gamma_{9,3,4,2} + \gamma_{10,4,5,3} - \gamma_{12,6,1,5} - \gamma_{7,1,2,6}$
38	$2\gamma_{8,2,3,1} - \gamma_{9,3,4,2} - \gamma_{10,4,5,3} + 2\gamma_{11,5,6,4} - \gamma_{12,6,1,5} - \gamma_{7,1,2,6}$
39	$\gamma_{9,3,4,2} - \gamma_{10,4,5,3} + \gamma_{12,6,1,5} - \gamma_{7,1,2,6}$

S3.16 benzene−ammonia dimer

Structure

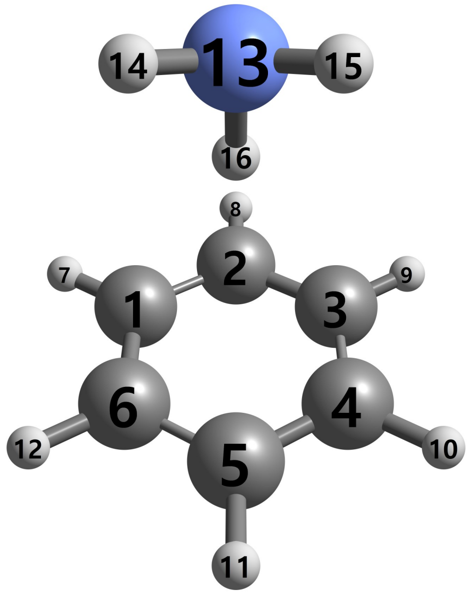

Figure S16: Benzene-ammonia dimer structure labeled with atomic indices. The plane of symmetry cuts vertically through C_2 and C_5, where $C_\sigma = C_2$.

Frequencies

Table S58: CMA-0A residuals with respect to the reference harmonic frequencies (in cm^{-1}) for benzene$\cdots NH_3$ using natural internal coordinates with cartesian force constants.

	Reference	CMA-0A	
	CCSD(T)	MP2	
	haTZ	TZ	haTZ
$\omega_1(a')$	3583.9	0.0	−0.1
$\omega_2(a')$	3457.3	0.0	0.1
$\omega_3(a')$	3202.9	0.0	0.0
$\omega_4(a')$	3192.1	0.0	0.0
$\omega_5(a')$	3175.7	−0.1	0.0
$\omega_6(a')$	3162.3	0.1	0.0
$\omega_7(a')$	1671.3	0.1	0.0
$\omega_8(a')$	1628.8	0.0	0.0
$\omega_9(a')$	1502.6	0.0	0.0
$\omega_{10}(a')$	1186.0	0.0	0.0
$\omega_{11}(a')$	1078.6	0.0	−0.2
$\omega_{12}(a')$	1051.1	0.0	0.2
$\omega_{13}(a')$	1010.7	0.0	0.0
$\omega_{14}(a')$	1001.8	0.0	0.0
$\omega_{15}(a')$	979.7	−2.2	−0.2
$\omega_{16}(a')$	976.0	−0.2	0.0
$\omega_{17}(a')$	857.8	0.0	0.0
$\omega_{18}(a')$	685.3	0.0	0.0
$\omega_{19}(a')$	681.0	3.3	0.3
$\omega_{20}(a')$	606.2	0.0	0.0
$\omega_{21}(a')$	397.1	0.1	0.0
$\omega_{22}(a')$	122.2	−0.4	0.0
$\omega_{23}(a')$	86.5	0.9	0.0
$\omega_{24}(a')$	43.3	2.8	0.3
$\omega_{25}(a'')$	3588.0	0.0	0.0
$\omega_{26}(a'')$	3192.9	0.0	0.0
$\omega_{27}(a'')$	3174.5	0.0	0.0
$\omega_{28}(a'')$	1671.3	0.1	0.1
$\omega_{29}(a'')$	1629.0	0.0	0.0
$\omega_{30}(a'')$	1502.4	0.0	0.0
$\omega_{31}(a'')$	1369.2	0.0	0.0
$\omega_{32}(a'')$	1329.8	−3.7	−4.0
$\omega_{33}(a'')$	1186.5	0.0	0.0
$\omega_{34}(a'')$	1155.6	4.3	4.6
$\omega_{35}(a'')$	1051.1	0.1	0.0
$\omega_{36}(a'')$	977.2	−0.1	0.0
$\omega_{37}(a'')$	858.5	0.0	0.0
$\omega_{38}(a'')$	606.4	0.0	0.0
$\omega_{39}(a'')$	397.8	0.1	0.0
$\omega_{40}(a'')$	66.3	−13.2	−0.2
$\omega_{41}(a'')$	18.5	25.0	1.5
$\omega_{42}(a'')$	5.6	9.8	0.6

Table S59: Pure level B residuals with respect to the reference harmonic frequencies (in cm^{-1}) for benzene$\cdots NH_3$ using natural internal coordinates with cartesian force constants.

	Reference	Pure	
	CCSD(T)	MP2	
	haTZ	TZ	haTZ
$\omega_{1}(a')$	3583.9	19.36	19.59
$\omega_{2}(a')$	3457.3	7.41	7.68
$\omega_{3}(a')$	3202.9	14.43	14.74
$\omega_{4}(a')$	3192.1	15.30	15.64
$\omega_{5}(a')$	3175.7	16.61	16.70
$\omega_{6}(a')$	3162.3	18.35	16.50
$\omega_{7}(a')$	1671.3	-7.49	-8.19
$\omega_{8}(a')$	1628.8	-7.89	-12.94
$\omega_{9}(a')$	1502.6	-7.72	-9.42
$\omega_{10}(a')$	1186.0	2.51	-0.44
$\omega_{11}(a')$	1078.6	-8.60	-12.99
$\omega_{12}(a')$	1051.1	0.22	-1.44
$\omega_{13}(a')$	1010.7	3.49	1.07
$\omega_{14}(a')$	1001.8	-0.36	-1.62
$\omega_{15}(a')$	979.7	-6.62	2.80
$\omega_{16}(a')$	976.0	16.90	-0.14
$\omega_{17}(a')$	857.8	2.55	2.36
$\omega_{18}(a')$	685.3	4.96	2.61
$\omega_{19}(a')$	681.0	2.20	5.31
$\omega_{20}(a')$	606.2	-3.93	-4.53
$\omega_{21}(a')$	397.1	3.31	0.80
$\omega_{22}(a')$	122.2	9.12	10.02
$\omega_{23}(a')$	86.5	-8.75	-5.86
$\omega_{24}(a')$	43.3	-4.76	-1.91
$\omega_{25}(a'')$	3588.0	24.08	26.10
$\omega_{26}(a'')$	3192.9	15.18	15.66
$\omega_{27}(a'')$	3174.5	16.59	16.72
$\omega_{28}(a'')$	1671.3	3.84	-7.35
$\omega_{29}(a'')$	1629.0	-7.76	-12.92
$\omega_{30}(a'')$	1502.4	-7.65	-9.37
$\omega_{31}(a'')$	1369.2	70.07	75.00
$\omega_{32}(a'')$	1329.8	32.66	33.23
$\omega_{33}(a'')$	1186.5	2.92	-0.36
$\omega_{34}(a'')$	1155.6	7.54	4.67
$\omega_{35}(a'')$	1051.1	0.60	-1.14
$\omega_{36}(a'')$	977.2	17.66	-0.23
$\omega_{37}(a'')$	858.5	2.15	2.30
$\omega_{38}(a'')$	606.4	-3.60	-4.47
$\omega_{39}(a'')$	397.8	3.60	0.84
$\omega_{40}(a'')$	66.3	76.03	-2.03
$\omega_{41}(a'')$	18.5	22.97	8.22
$\omega_{42}(a'')$	5.6	51.19	-30.21

Natural Internal Coordinates

Table S60: Symmetrized, unnormalized natural internal coordinates for benzene−ammonia dimer.

1	$r_{6,1} + r_{1,2} + r_{2,3} + r_{3,4} + r_{4,5} + r_{5,6}$
2	$r_{6,1} - r_{1,2} + r_{2,3} - r_{3,4} + r_{4,5} - r_{5,6}$
3	$2r_{6,1} + r_{1,2} - r_{2,3} - 2r_{3,4} - r_{4,5} + r_{5,6}$
4	$r_{1,2} + r_{2,3} - r_{4,5} - r_{5,6}$
5	$2r_{6,1} - r_{1,2} - r_{2,3} + 2r_{3,4} - r_{4,5} - r_{5,6}$
6	$r_{1,2} - r_{2,3} + r_{4,5} - r_{5,6}$
7	$r_{2,8} + r_{3,9} + r_{4,10} + r_{5,11} + r_{6,12} + r_{1,7}$
8	$r_{2,8} - r_{3,9} + r_{4,10} - r_{5,11} + r_{6,12} - r_{1,7}$
9	$2r_{2,8} + r_{3,9} - r_{4,10} - 2r_{5,11} - r_{6,12} + r_{1,7}$
10	$r_{3,9} + r_{4,10} - r_{6,12} - r_{1,7}$
11	$2r_{2,8} - r_{3,9} - r_{4,10} + 2r_{5,11} - r_{6,12} - r_{1,7}$
12	$r_{3,9} - r_{4,10} + r_{6,12} - r_{1,7}$
13	$r_{13,16} + r_{13,14} + r_{13,15}$
14	$2r_{13,16} - r_{13,14} - r_{13,15}$
15	$r_{13,14} - r_{13,15}$
16	$r(13\|1,2,3,4,5,6)$
17	$\phi_{1,2,3} - \phi_{2,3,4} + \phi_{3,4,5} - \phi_{4,5,6} + \phi_{5,6,1} - \phi_{6,1,2}$
18	$2\phi_{1,2,3} - \phi_{2,3,4} - \phi_{3,4,5} + 2\phi_{4,5,6} - \phi_{5,6,1} - \phi_{6,1,2}$
19	$\phi_{2,3,4} - \phi_{3,4,5} + \phi_{5,6,1} - \phi_{6,1,2}$
20	$\phi_{8,2,3} - \phi_{8,2,1} + \phi_{9,3,4} - \phi_{9,3,2} + \phi_{10,4,5} - \phi_{10,4,3} + \phi_{11,5,6} - \phi_{11,5,4} + \phi_{12,6,1} - \phi_{12,6,5}$ $+\phi_{7,1,2} - \phi_{7,1,6}$
21	$\phi_{8,2,3} - \phi_{8,2,1} - \phi_{9,3,4} + \phi_{9,3,2} + \phi_{10,4,5} - \phi_{10,4,3} - \phi_{11,5,6} + \phi_{11,5,4} + \phi_{12,6,1} - \phi_{12,6,5}$ $-\phi_{7,1,2} + \phi_{7,1,6}$
22	$2\phi_{8,2,3} - 2\phi_{8,2,1} + \phi_{9,3,4} - \phi_{9,3,2} - \phi_{10,4,5} + \phi_{10,4,3} - 2\phi_{11,5,6} + 2\phi_{11,5,4} - \phi_{12,6,1} + \phi_{12,6,5}$ $+\phi_{7,1,2} - \phi_{7,1,6}$
23	$\phi_{9,3,4} - \phi_{9,3,2} + \phi_{10,4,5} - \phi_{10,4,3} - \phi_{12,6,1} + \phi_{12,6,5} - \phi_{7,1,2} + \phi_{7,1,6}$
24	$2\phi_{8,2,3} - 2\phi_{8,2,1} - \phi_{9,3,4} + \phi_{9,3,2} - \phi_{10,4,5} + \phi_{10,4,3} + 2\phi_{11,5,6} - 2\phi_{11,5,4} - \phi_{12,6,1} + \phi_{12,6,5}$ $-\phi_{7,1,2} + \phi_{7,1,6}$
25	$\phi_{9,3,4} - \phi_{9,3,2} - \phi_{10,4,5} + \phi_{10,4,3} + \phi_{12,6,1} - \phi_{12,6,5} - \phi_{7,1,2} + \phi_{7,1,6}$
26	$2\phi_{14,13,15} - \phi_{14,13,16} - \phi_{15,13,16}$
27	$\phi_{14,13,16} - \phi_{15,13,16}$
28	$\phi(2\|1,2,3,4,5,6\|13) - \phi(5\|1,2,3,4,5,6\|13)$
29	$\phi(1,6\|1,2,3,4,5,6\|13) - \phi(3,4\|1,2,3,4,5,6\|13)$
30	$2\phi(1,2,3,4,5,6\|13\|16) - \phi(1,2,3,4,5,6\|13\|14) - \phi(1,2,3,4,5,6\|13\|15)$
31	$\phi(1,2,3,4,5,6\|13\|14) - \phi(1,2,3,4,5,6\|13\|15)$
32	$\tau_{1,2,3,4} - \tau_{2,3,4,5} + \tau_{3,4,5,6} - \tau_{4,5,6,1} + \tau_{5,6,1,2} - \tau_{6,1,2,3}$
33	$\tau_{1,2,3,4} - \tau_{3,4,5,6} + \tau_{4,5,6,1} - \tau_{6,1,2,3}$
34	$-\tau_{1,2,3,4} + 2\tau_{2,3,4,5} - \tau_{3,4,5,6} - \tau_{4,5,6,1} + 2\tau_{5,6,1,2} - \tau_{6,1,2,3}$
35	$\tau(2\|1,2,3,4,5,6\|13\|16) + \tau(2\|1,2,3,4,5,6\|13\|14) + \tau(2\|1,2,3,4,5,6\|13\|15)$
36	$\gamma_{8,2,3,1} + \gamma_{9,3,4,2} + \gamma_{10,4,5,3} + \gamma_{11,5,6,4} + \gamma_{12,6,1,5} + \gamma_{7,1,2,6}$
37	$\gamma_{8,2,3,1} - \gamma_{9,3,4,2} + \gamma_{10,4,5,3} - \gamma_{11,5,6,4} + \gamma_{12,6,1,5} - \gamma_{7,1,2,6}$
38	$2\gamma_{8,2,3,1} + \gamma_{9,3,4,2} - \gamma_{10,4,5,3} - 2\gamma_{11,5,6,4} - \gamma_{12,6,1,5} + \gamma_{7,1,2,6}$
39	$\gamma_{9,3,4,2} + \gamma_{10,4,5,3} - \gamma_{12,6,1,5} - \gamma_{7,1,2,6}$
40	$2\gamma_{8,2,3,1} - \gamma_{9,3,4,2} - \gamma_{10,4,5,3} + 2\gamma_{11,5,6,4} - \gamma_{12,6,1,5} - \gamma_{7,1,2,6}$
41	$\gamma_{9,3,4,2} - \gamma_{10,4,5,3} + \gamma_{12,6,1,5} - \gamma_{7,1,2,6}$
42	$\gamma_{16,13,14,15} + \gamma_{14,13,15,16} + \gamma_{15,13,16,14}$

S3.17 ethylene−acetylene dimer

Structure

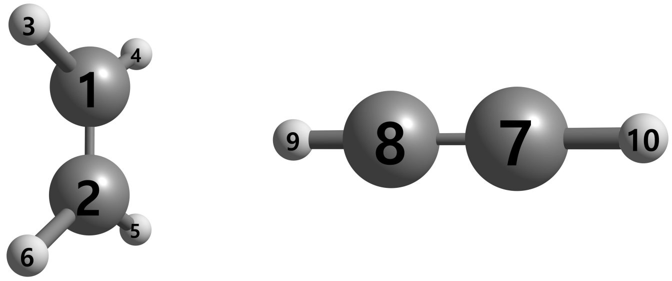

Figure S17: Ethylene-Acetylene dimer structure labeled with atomic indices.

Frequencies

Table S61: CMA-0A residuals with respect to the reference harmonic frequencies (in cm^{-1}) for $C_2H_4 \cdots C_2H_2$ using natural internal coordinates with cartesian force constants.

	Reference	CMA-0A							
	CCSD(T)	MP2						CCSD(T)	
	aTZ	DZ	haDZ	aDZ	TZ	haTZ	aTZ	DZ	haDZ
$\omega_1(a_1)$	3486.8	−0.4	−0.4	0.0	−0.3	−0.4	−0.1	−0.1	−0.1
$\omega_2(a_1)$	3375.3	0.3	0.4	0.0	0.3	0.4	0.1	0.1	0.1
$\omega_3(a_1)$	3149.3	0.0	−0.1	0.0	0.0	0.0	0.0	0.0	−0.1
$\omega_4(a_1)$	1989.5	0.0	0.0	0.0	0.0	0.0	0.0	0.0	0.0
$\omega_5(a_1)$	1663.8	−1.3	−1.4	−1.3	0.0	0.0	0.0	−1.1	−1.2
$\omega_6(a_1)$	1364.4	1.7	1.8	1.7	0.0	0.0	0.0	1.4	1.6
$\omega_7(a_1)$	967.2	0.0	0.0	0.0	0.0	0.0	0.0	0.0	0.0
$\omega_8(a_1)$	78.4	0.1	0.9	0.7	0.2	0.2	0.1	0.1	1.7
$\omega_9(a_2)$	3213.5	0.0	0.0	0.0	0.0	0.0	0.0	0.0	0.0
$\omega_{10}(a_2)$	1240.8	0.0	0.0	0.0	0.0	0.0	0.0	0.0	0.0
$\omega_{11}(a_2)$	1042.9	0.0	0.0	0.0	0.0	0.0	0.0	0.0	0.0
$\omega_{12}(b_1)$	3131.7	0.0	0.0	0.0	0.0	0.0	0.0	0.0	0.0
$\omega_{13}(b_1)$	1472.8	0.0	0.0	0.0	0.0	0.0	0.0	0.0	0.0
$\omega_{14}(b_1)$	936.4	0.0	0.0	0.0	0.0	0.0	0.0	0.0	0.0
$\omega_{15}(b_1)$	775.4	−0.7	−0.5	−0.1	0.0	0.0	0.0	−0.4	−0.5
$\omega_{16}(b_1)$	611.9	1.5	0.6	−0.3	0.0	0.1	0.0	0.6	0.6
$\omega_{17}(b_1)$	82.3	0.0	−0.8	2.0	−0.3	−0.2	0.0	0.2	−1.5
$\omega_{18}(b_1)$	33.6	0.2	0.1	0.3	0.5	0.3	0.0	0.3	0.1
$\omega_{19}(b_2)$	3239.9	0.0	0.0	0.0	0.0	0.0	0.0	0.0	0.0
$\omega_{20}(b_2)$	817.6	0.1	0.0	0.0	0.0	0.0	0.0	0.0	0.0
$\omega_{21}(b_2)$	780.0	−0.5	−0.4	−0.3	−0.3	−0.1	0.0	−0.3	−0.4
$\omega_{22}(b_2)$	613.4	0.1	0.5	0.0	0.4	0.1	0.0	0.4	0.5
$\omega_{23}(b_2)$	99.4	−0.3	−0.3	0.9	−0.6	−0.2	−0.1	−0.7	0.0
$\omega_{24}(b_2)$	35.4	1.2	1.3	3.8	1.8	0.6	0.4	2.3	0.9

Table S62: Pure level B residuals with respect to the reference harmonic frequencies (in cm^{-1}) for $C_2H_4 \cdots C_2H_2$ using natural internal coordinates with cartesian force constants.

	Reference	Pure							
	CCSD(T)	MP2						CCSD(T)	
	aTZ	DZ	haDZ	aDZ	TZ	haTZ	aTZ	DZ	haDZ
$\omega_1(a_1)$	3486.8	156.36	139.11	130.37	15.98	9.58	59.07	59.07	59.07
$\omega_2(a_1)$	3375.3	166.96	145.21	139.83	22.61	22.25	65.21	65.21	65.21
$\omega_3(a_1)$	3149.3	155.88	132.72	126.30	16.12	15.16	52.31	52.31	52.31
$\omega_4(a_1)$	1989.5	84.12	79.32	72.95	−14.85	−15.88	47.66	47.66	47.66
$\omega_5(a_1)$	1663.8	55.66	49.63	46.10	−2.48	−4.37	71.85	71.85	71.85
$\omega_6(a_1)$	1364.4	35.08	32.34	34.20	6.98	5.47	58.33	58.33	58.33
$\omega_7(a_1)$	967.2	29.23	22.69	37.67	16.31	11.89	105.51	105.51	105.51
$\omega_8(a_1)$	78.4	7.39	2.18	4.14	−3.24	−2.35	20.68	20.68	20.68
$\omega_9(a_2)$	3213.5	175.21	148.85	141.67	22.49	21.99	44.07	44.07	44.07
$\omega_{10}(a_2)$	1240.8	13.45	7.78	5.95	−1.51	−0.94	78.71	78.71	78.71
$\omega_{11}(a_2)$	1042.9	40.87	34.67	29.16	29.48	25.75	75.33	75.33	75.33
$\omega_{12}(b_1)$	3131.7	154.86	130.60	125.49	17.34	15.81	52.46	52.46	52.46
$\omega_{13}(b_1)$	1472.8	18.94	16.04	18.92	6.23	3.33	88.94	88.94	88.94
$\omega_{14}(b_1)$	936.4	26.53	25.36	43.79	18.56	15.61	138.89	138.89	138.89
$\omega_{15}(b_1)$	775.4	40.22	−12.91	−9.61	5.18	1.43	108.80	108.80	108.80
$\omega_{16}(b_1)$	611.9	36.83	−22.21	−98.53	−4.26	13.83	148.17	148.17	148.17
$\omega_{17}(b_1)$	82.3	9.74	−1.04	−7.70	5.84	4.73	29.67	29.67	29.67
$\omega_{18}(b_1)$	33.6	10.29	1.79	2.22	0.64	−2.09	18.44	18.44	18.44
$\omega_{19}(b_2)$	3239.9	173.32	149.85	141.78	22.12	22.36	45.62	45.62	45.62
$\omega_{20}(b_2)$	817.6	21.49	15.37	17.02	2.65	1.18	67.55	67.55	67.55
$\omega_{21}(b_2)$	780.0	39.61	−12.25	−12.07	−2.20	−1.34	89.44	89.44	89.44
$\omega_{22}(b_2)$	613.4	35.39	−20.18	−104.10	−16.05	13.35	146.58	146.58	146.58
$\omega_{23}(b_2)$	99.4	1.61	12.66	22.21	2.13	3.37	18.93	18.93	18.93
$\omega_{24}(b_2)$	35.4	7.78	16.90	23.79	1.32	1.53	18.43	18.43	18.43

Natural Internal Coordinates

Table S63: Symmetrized, unnormalized natural internal coordinates for ethylene−acetylene dimer.

1	$r_{1,2}$
2	$r_{1,3} + r_{1,4} + r_{2,6} + r_{2,5}$
3	$r_{1,3} + r_{1,4} - r_{2,6} - r_{2,5}$
4	$r_{1,3} - r_{1,4} + r_{2,6} - r_{2,5}$
5	$r_{1,3} - r_{1,4} - r_{2,6} + r_{2,5}$
6	$r_{7,8}$
7	$r_{7,10} + r_{8,9}$
8	$r_{7,10} - r_{8,9}$
9	$r(9\|2,1)$
10	$2\phi_{3,1,4} - \phi_{3,1,2} - \phi_{4,1,2} + 2\phi_{6,2,5} - \phi_{6,2,1} - \phi_{5,2,1}$
11	$2\phi_{3,1,4} - \phi_{3,1,2} - \phi_{4,1,2} - 2\phi_{6,2,5} + \phi_{6,2,1} + \phi_{5,2,1}$
12	$\phi_{3,1,2} - \phi_{4,1,2} + \phi_{6,2,1} - \phi_{5,2,1}$
13	$\phi_{3,1,2} - \phi_{4,1,2} - \phi_{6,2,1} + \phi_{5,2,1}$
14	$\phi(9\|2,1\|1,3,4) - \phi(9\|2,1\|2,5,6)$
15	$\tau_{3,1,2,5} + \tau_{3,1,2,6} + \tau_{4,1,2,5} + \tau_{4,1,2,6}$
16	$\tau(9\|2,1\|2,5,6\|3,6) - \tau(9\|2,1\|1,3,4\|4,5)$
17	$\gamma_{1,2,5,6} + \gamma_{2,1,3,4}$
18	$\gamma_{1,2,5,6} - \gamma_{2,1,3,4}$
19	$\gamma(2,1\|9\|13\|8)$
20	$\gamma(2,1\|9\|14\|8)$
21	$\theta_{8,7,10,11} + \theta_{9,8,7,13}$
22	$\theta_{8,7,10,11} - \theta_{9,8,7,13}$
23	$\theta_{8,7,10,12} + \theta_{9,8,7,14}$
24	$\theta_{8,7,10,12} - \theta_{9,8,7,14}$

References

(1) Handy, N. C.; Maslen, P. E.; Amos, R. D.; Andrews, J. S.; Murray, C. W.; Laming, G. J. The Harmonic Frequencies of Benzene. *Chem. Phys. Lett.* **1992**, *197*, 506–515.

(2) Lane, J. R. CCSDTQ Optimized Geometry of Water Dimer. *J. Chem. Theory Comput.* **2013**, *9*, 316–323.

(3) Janeiro-Barral, P. E.; Mella, M. Study of the Structure, Energetics, and Vibrational Properties of Small Ammonia Clusters $(NH_3)_n$ (n = 2–5) Using Correlated ab Initio Methods. *J. Phys. Chem. A* **2006**, *110*, 11244–11251.

(4) Řezáč, J.; Hobza, P. Describing Noncovalent Interactions beyond the Common Approximations: How Accurate Is the "Gold Standard," CCSD(T) at the Complete Basis Set Limit? *J. Chem. Theory Comput.* **2013**, *9*, 2151–2155.

(5) Miliordos, E.; Xantheas, S. S. On the Validity of the Basis Set Superposition Error and Complete Basis Set Limit Extrapolations for the Binding Energy of the Formic Acid Dimer. *J. Chem. Phys.* **2015**, *142*, 094311.

(6) Alessandrini, S.; Puzzarini, C. Structural and Energetic Characterization of Prebiotic Molecules: The Case Study of Formamide and Its Dimer. *J. Phys. Chem. A* **2016**, *120*, 5257–5263.

(7) Tzeli, D.; Mavridis, A.; Xantheas, S. S. A First Principles Study of the Acetylene–Water Interaction. *J. Chem. Phys.* **2000**, *112*, 6178–6189.

(8) Howard, J. C.; Gray, J. L.; Hardwick, A. J.; Nguyen, L. T.; Tschumper, G. S. Getting down to the Fundamentals of Hydrogen Bonding: Anharmonic Vibrational Frequencies of $(HF)_2$ and $(H_2O)_2$ from Ab Initio Electronic Structure Computations. *J. Chem. Theory Comput.* **2014**, *10*, 5426–5435.

(9) Howard, J. C.; Tschumper, G. S. Benchmark Structures and Harmonic Vibrational Frequencies Near the CCSD(T) Complete Basis Set Limit for Small Water Clusters: $(H_2O)_{n=2,3,4,5,6}$. *J. Chem. Theory Comput.* **2015**, *11*, 2126–2136.
